# Test-retest reliability of EEG microstate metrics for evaluating noise reductions in simultaneous EEG-fMRI

**DOI:** 10.1162/imag_a_00272

**Published:** 2024-08-29

**Authors:** Toshikazu Kuroda, Reinmar J. Kobler, Takeshi Ogawa, Mizuki Tsutsumi, Tomohiko Kishi, Motoaki Kawanabe

**Affiliations:** Cognitive Mechanisms Laboratories, Advanced Telecommunications Research Institute International, Hikaridai, Seika-cho, Soraku-gun, Kyoto, Japan; RIKEN Center for Advanced Intelligence Project, Hikaridai, Seika-cho, Soraku-gun, Kyoto, Japan

**Keywords:** simultaneous EEG-fMRI, multimodal, noise reduction, microstate, reliability

## Abstract

Simultaneous electroencephalography-functional magnetic resonance imaging (EEG-fMRI) has potential for elucidating brain activities but suffers from severe noise/artifacts in EEG. While several countermeasures have been developed, it remains difficult to evaluate noise reductions in the absence of ground truth in EEG. We introduce a new evaluation method which takes advantage of high test-retest reliability of EEG microstate metrics. We assumed, if the reliability is high for a pair of EEG recorded outside an MR scanner on two different days, then it should also be high for a pair of EEG recorded inside and outside the scanner on the same day if MR-induced noise is absent. Thus, noise should be removed in a way that the reliability increases. Accordingly, we obtained EEG both inside and outside the scanner on two different days. Using ICC as an index, we examined test-retest reliability for 1) a pair of EEG outside the scanner across the days, 2) a pair of EEG inside and outside the scanner on the same day, and 3) a pair of EEG inside the scanner across the days. MR-induced noise, BCG artifact in particular, was reduced with joint decorrelation with varying thresholds. We obtained moderately high reliability in all the three pairs (ICCs > 0.5), suggesting sufficient noise reductions. Taking these steps, the quality of EEG improved as assessed with its traces, power spectra density, and microstate templates in resting state as well as event-related potentials in a visual oddball task. We discuss advantages and limitations of this new evaluation method.

## 1 Introduction

Electroencephalography (EEG) and functional magnetic resonance imaging (fMRI) are two major noninvasive approaches for measuring brain activity. EEG and fMRI provide fundamentally different views to neural activity. EEG directly measures electrophysiological signals with a high temporal resolution in a millisecond order but is low in spatial resolution. This limits its sensitivity to large-scale brain network activity primarily originating from coordinated postsynaptic potentials at large pyramidal cells in the cortex. fMRI indirectly measures neuronal activity via blood-oxygenation-level-dependent (BOLD) signals, which reflect blood flow, blood volume, or oxygen metabolism (Buxton, 2002). Compared to EEG, fMRI has a high spatial resolution in a millimeter order but is low in temporal resolution, with BOLD signals being 3–6 s delayed from the actual neural activity (i.e., hemodynamic response function). Given these properties, EEG and fMRI data have been considered complementary to each other. Measuring the two separately in time, for instance, fMRI has been used as priors for localizing the EEG cortical current source (e.g.,Baillet & Garnero, 1997;Henson et al., 2011;Sato et al., 2004;van Graan et al., 2015). Their simultaneous recording has a further advantage in examining how EEG is correlated with fMRI (e.g.,Britz et al., 2010;Mantini et al., 2007; seeJorge et al., 2014for a review). As an extension of the latter, some researchers have attempted to predict (delayed) BOLD signals from EEG (Abreu et al., 2021;Cury et al., 2020;Meir-Hasson et al., 2014), which has potential for neurofeedback with both high spatial and temporal resolutions (Caetano et al., 2023;Fleury et al., 2023;Levitt et al., 2023).

Though promising, simultaneous EEG-fMRI suffers from serious contamination of noise in EEG. In addition to the absence of a shield room commonly used for recording EEG, MR scanners themselves are a harsh environment for EEG recording, providing multiple sources of noise. Gradient artifact (GA), typically the biggest noise in amplitude, results from switching in the gradient system that changes the magnetic field to accommodate phase/frequency encoding (Allen et al., 2000). A helium pump, often embedded in the scanner to cool down the system for generating a high magnetic field, produces vibration which, in turn, produces large artifacts in EEG (Rothlübbers et al., 2015). Besides these sources of noise, human body motions also produce artifacts. When a head moves, for example, EEG electrodes and their associated wires move along, generating current flows in the magnetic field. Hereafter, motion artifact (MA) will be referred to these types of motion-related noise. Ballistocardiogram (BCG) artifact has been one of the most challenging MAs due to the similarity in amplitude between BCG and EEG and to its spectral profile (approximately 1 Hz and its harmonics) overlapping with the delta, theta, and possibly alpha frequency bands as well (i.e., an overall range of 1-12 Hz;Abreu et al., 2022). Moreover, the mechanism generating BCG is complex involving some hypothesized factors such as bulk head motion at the arrival of blood from the heart, pulsatile scalp dilation, and electric potentials given the flow of ions in blood in a magnetic field.

Researchers have developed countermeasures for artifacts associated with fMRI scanning (seeAbreu et al., 2022for a review). GA is precisely repetitive so that it can effectively be attenuated by applying average artifact subtraction (AAS;Allen et al., 2000) based on recorded trigger signals (e.g., every volume/slice). In this method, some volumes around the current volume are averaged for creating a template waveform used for subtraction. AAS has also been used for reducing BCG (Allen et al., 1998). Its variant called optimal basis set (OBS) reduces BCG by first decomposing EEG during heartbeats into principal components (Niazy et al., 2005). Independent component analysis (ICA) capturing spatiotemporal patterns is another type of method for BCG reduction (e.g.,Abreu et al., 2016;Debener et al., 2007). Reference layer artifact subtraction (RLAS;Chowdhury et al., 2014;Levitt et al., 2023) and carbon-wire loop (CWL;van der Meer et al., 2016) are sensor-based methods for removing MAs including BCG by measuring those artifacts in isolation from EEG (see alsoJorge et al., 2015).

Despite these efforts, it remains difficult to evaluate noise reductions due to the absence of ground truth in EEG. Its absence may be less of an issue when recording EEG alone. This is because GA and most MAs are physically absent in a shield room, and other types of artifacts such as eye movements can effectively be removed using other techniques (e.g., ICLabel pre-trained classifier;Kobler et al., 2019,^2020^;Pion-Tonachini et al., 2019). Thus, the resultant EEG can be considered “noise free” though not perfect. In simultaneous EEG-fMRI, in contrast, it is impossible to obtain noise-contaminated EEG with “noise-free” EEG signals at the same time (Lin et al., 2022) although the latter is necessary for evaluating whether noise reduction is sufficient, insufficient, or excessive.

There are several possible remedies for evaluating noise reductions in the absence of ground truth in EEG. One remedy is to check the presence of physiologically meaningful signals such as alpha power using an eye open/closed procedure (van der Meer et al., 2016), steady-state visual-evoked potential (Levitt et al., 2023), and event-related potential (ERP;Abreu et al., 2016). The presence of such signals, however, would only indicate that noise reduction is not excessive but not that it is sufficient. Another remedy is the use of EEG recorded outside the MR scanner as the ground truth by proxy. For instance,van der Meer et al. (2016)computed the normalized Euclidean distance of power spectra density (PSD;Aggarwal et al., 2001) between inside and outside MR scanners, with values approaching zero supposedly indicating better noise reductions. Nonetheless, PSD is a summary of EEG signals lacking temporal information, which is crucial for analyzing EEG in its relation to fMRI. Also, the use of EEG outside the scanner as the ground truth by proxy is based on an implicit assumption of test-retest reliability: EEG should be similar between inside and outside the scanner if MR-induced noise is absent.

EEG microstates could be a useful alternative for the evaluation of noise reductions. Briefly, microstates are patterns of scalp potential topography maps, each lasting for a certain duration before transitioning to a different map. Microstate templates are extracted by clustering the EEG topography maps, typically resulting in four to five templates (seeMichel & Koenig, 2018for a review). Backfitting the templates to raw EEG provides several metrics such as duration, occurrence, coverage, and transition for each microstate which capture the temporal dynamics of EEG. Moreover, test-retest reliability of those metrics has been shown to be high at least for EEG recorded outside MR scanners (Antonova et al., 2022;Khanna et al., 2014;Kleinert et al., 2023;Liu et al., 2020; but seePopov et al., 2023). For instance,Kleinert et al. (2023)used intraclass correlation coefficients (ICC) as an index of test-retest reliability. With a group of more than 500 participants, the ICC was around 0.9 (high reliability) for duration, occurrence, and coverage but 0.4 (low reliability) for transition when the first and second EEG recordings approximately were 2 hours apart. When the recordings were 6 months apart, the ICC was around 0.7 (moderately high reliability) for the first three metrics and 0.3 for transition.

Here, we introduce a novel evaluation method for noise reduction in simultaneous EEG-fMRI taking advantage of the test-retest reliability of EEG microstate metrics. Specifically, we assumed that, if the reliability is high for a pair of EEG recorded outside an MR scanner on*two different days*, then it should also be high for a pair of EEG recorded inside and outside the scanner on*the same day*if MR-induced noise is absent. This led to the notion that noise should be removed in a way that the reliability increases (seeFig. 1). Taking this approach, we recorded EEG both inside and outside the scanner on two different days per participant. Using ICC as an index, test-retest reliability of microstate metrics was assessed 1) for a pair of EEG outside the scanner across the two days, 2) for a pair of EEG inside and outside the scanner on the same day, 3) and for a pair of EEG inside the scanner across the two days. For inside-EEG, we focused on reducing BCG artifacts for them being the most challenging one (Abreu et al., 2022). We obtained moderately high test-retest reliability in all the three pairs after an identical set of noise reductions between inside and outside the scanner except for some additional steps for reducing scanning-related noise.

**Fig. 1.**
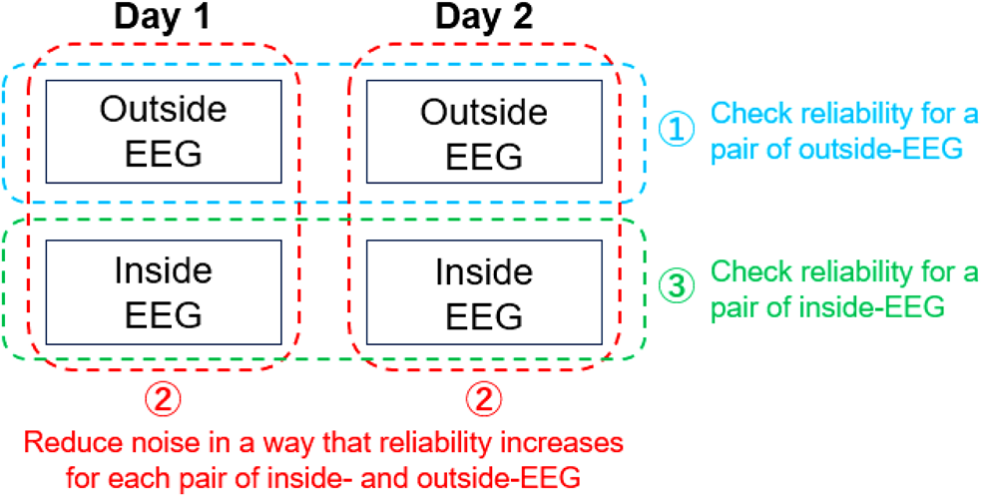
A diagram for the proposed evaluation method for noise reduction in simultaneous EEG-fMRI. Step 1: Confirm test-retest reliability for a pair of EEG recorded outside an MR scanner (*Outside-EEG*) on two different days, serving as a prerequisite for further steps. Step 2: Reduce MR-induced noise in a way that the reliability increases for a pair of EEG recorded inside the scanner (*Inside-EEG*) within a day. Step 3: Check the reliability for a pair of inside-EEG across the two days.

## 2 Materials and Methods

### 2.1 Experimental settings

Experimental tasks were conducted both inside and outside an MR scanner, where the latter refers to a soundproof shield room. The wall of the shield room was made with a copper mesh for blocking electromagnetic noise from outside. The interior size of the shield room was 145 cm wide, 185 cm long, and 210 cm high. The room had a table, a comfortable chair, two ceiling lights, and an air conditioner. On the table were a conventional keyboard and a Dell® computer monitor (34 cm by 60 cm) with its center approximately being the eye level when participants sat on the chair. A photo sensor was attached to a corner of the monitor, connected to a StimTracker (Cedrus® Model ST-100), for recording time at which stimuli actually were presented. The ceiling lights and the air conditioner each were covered with a metal-mesh box for minimizing noise. A pilot study indicated that EEG recorded inside the shield room was quite clean except for the presence of 60-Hz line noise from a power outlet (see the top panel inSupplementary Fig. S1).

Simultaneous EEG-fMRI was recorded inside 3T MAGNETOM Prisma fit (Siemens, Germany) with its software of version syngo MR VE11C/E. Visual stimuli were presented on an opaque screen through a projector (Cannon Power Projector, WUX6000 with 60 Hz frame rate) that participants could see on a small mirror attached to a head coil. As in the shield room, a photo sensor was attached to a corner of the screen. An MRI-compatible response device (HHSC-2 × 2, Current Designs, Inc., PA, USA) was used for measuring button presses.

### 2.2 Experimental procedures

The protocol of this study was approved by the ATR Review Board Ethics Committee (application number: 167) and followed the Declaration of Helsinki. All instructions including those during experimental tasks were given in Japanese. Twenty-seven participants (10 men, 17 women) were recruited with the following criteria: 1) ages ranging from 20 to 49; and 2) no health issue reported. All the participants provided written informed consent prior to their participation. EEG recording started in the shield room and, shortly after (approximately in 30 min), inside the MR scanner. In both settings, EEG was recorded under resting state first and then in a visual oddball task (In addition, an N-back task consisting of 0-back and 2-back conditions using numbers 0–9 as stimuli was conducted following the oddball task, which was not analyzed in the present study). This was repeated for two days. Intervals between the two recording days varied from 1 to 23 days (mean: 7.46; median: 4.50) across participants.

EEG in resting state with eyes open was measured for 8 min in each recording. Participants were instructed to keep staring blankly at a fixation cross (“+”) presented at the center of the screen without pondering anything specific and without falling asleep. A visual oddball task (hereafter, just*oddball*) consisted of 250 trials, each lasting for 1300 ms. Each trial consisted of the following components: a pre-stimulus period for 650 ms (plus random jitters ranging from -100 to 100 ms in steps of 2 ms); a stimulus presentation for 200 ms; and a post-stimulus period for 450 ms (minus the pre-stimulus jitters to keep the trial duration constant). A circle image was presented as a normal stimulus at*p = *.8 on average across participants, and a star image as a target stimulus at*p = *.2 on average. Each shape image was 125 px by 125 px in size. The actual number of trials with the target stimulus randomly varied across participants from 47 to 53 in steps of 1. After the task, they were asked to verbally report how many times the target stimulus was presented for checking to see if they were attending to the task (but without penalty for incorrect answers). The order of stimuli was quasi-random in a way that the target stimulus was never presented twice in a row and never presented in the first three trials as well as in the last trial. During the pre- and post-stimulus periods, the fixation cross was presented at the center of the screen. All the visual stimuli described here were dark bluish/greenish (i.e., 47, 79, 79 in RGB colors), presented on a black background.

There was an approximately 2-min break between the tasks, during which the instructions about the upcoming task were given. In addition, when the tasks were conducted inside the MR scanner, there were a 40-s pre-task period and a 30-s post-task period during which the fixation cross remained on. These additional periods were for stabilizing the magnetic field in the scanner and also for removing GA later. A Panasonic® laptop computer with Python 3.9 controlled all the experimental tasks.

Among 27 participants, data for 2 and 3 participants were excluded from analyses respectively for resting state and oddball tasks due to some issues with their data. For resting state, one participant fell asleep during recordings. For another participant, EEG was quite noisy even for those recorded outside the scanner. We excluded the latter participant’s oddball data for the same reason. Two other participants in the oddball task showed very strong eye artifacts that could not be removed with the method described below. These led us to having 25 participants (8 men, 17 women) and 24 participants (9 men, 15 women) for the resting-state and oddball tasks, respectively.

### 2.3 Data acquisitions of EEG and fMRI

EEG signals were recorded with MR-compatible EEG caps and amplifiers (BrainAmp MR plus, BrainAmp ExG MR, and BrainCap MR; Brain Products GmbH, Germany), with each cap having 63 EEG channels, one electrocardiogram (ECG) channel, and four carbon-wire loop (CWL) channels. An appropriate size of the cap (54, 56, and 58 cm) was selected for each participant.

The EEG electrodes were placed on the scalp according to a modified International 10-20 system. The ground and reference electrodes were placed on AFz and FCz, respectively. The ECG electrode was placed on the back of the participant. Conductive gel (Abralyt HiCl, EASYCAP; Brain Products GmbH, Germany) was used to keep impedance of all electrodes below 10 kΩ throughout the experiment. Raw EEG data were sampled at 5 kHz. EEG and fMRI data were recorded simultaneously via hardware clock synchronization (SyncBox, BrainProducts, Germany). The scanner also delivered trigger signals that marked the onset time of fMRI volume acquisitions on EEG data.

MRI data were acquired with 3T MAGNETOM Prisma fit with 64-channel head coils (Siemens, Erlangen, Germany). Our scanning protocol followed the Multimodel Harmonized MRI Imaging Protocol (HARP; seehttps://hbm.brainminds-beyond.jp/documents/protocol.html). We obtained T1 (repetition time [TR] = 2500 ms, echo time [TE] = 2.18 ms, flip angle = 8 deg, inversion time = 1000 ms, matrix = 300 x 320, 224 sagittal slices, 0.8 mm isotropic) and T2 structural images (TR = 3200 ms, TE = 564 ms, flip angle = 120 deg, matrix = 300 x 320, 224 sagittal slices, 0.8 mm isotropic) as well as fieldmaps (both posterior to anterior [PA] and anterior to posterior [AP] sequences; TR = 6100 ms, TE = 60 ms, flip angle = 90 deg, matrix = 86 x 86, 60 sagittal slices, 2.4 mm isotropic) for each participant. Multi-band echo planar images (EPIs; both PA and AP sequences) were acquired with the following parameters: TR = 800 ms; TE = 34.4 ms; flip angle = 52 deg; matrix = 86 × 86; field of view = 205.9 mm; slice thickness = 2.4 mm; 60 axial slices with a multi-band factor of 6. The scanning durations were 9 min 15 s (694 volumes) for resting state and 6 min 38 s (498 volumes) for the oddball task. The helium pump remained on throughout the recording.

### 2.4 EEG preprocessing

EEG data were preprocessed individually for each recording, taking steps described inTable 1. All data (i.e., EEG and fMRI) were organized to conform with the Brain Imaging Data Structure (BIDS) format (Gorgolewski et al., 2016). EEG files were converted to EEGLAB format (Delorme & Makeig, 2004).

**Table 1.** A summary of EEG preprocessing steps.

Outside-EEG	Inside-EEG
BIDS format	BIDS format
1. High-pass filter (0.5 Hz)	1. High-pass filter (0.5 Hz)
	2. GA correction with AAS
3. Low-pass filter (125 Hz)	3. Low-pass filter (125 Hz)
	4. Resampling (500 Hz)
	5. CWL regression for reducing motion-related artifacts
6. Resampling (250 Hz)	6. Resampling (250 Hz)
EEGLAB format	EEGLAB format
	7. Joint decorrelation with varying thresholds for reducing residual BCG
8. Band-pass filter (2-20 Hz) with *pop_eegfiltnew*	
9. Artifact removal based on spectrum thresholding with *pop_rejcont* (15-30 Hz)	
10. Re-reference data to the average with *pop_reref* and *pop_select*	
11. Remove noisy channel with *clean_rawdata* followed by interpolation with *pop_interp*	
12. ICA with f *astica*	
13. ICLabel with an identical set of thresholds for artifact components between inside- and outside-EEG	

Notes: Brain Imaging Data Structure (BIDS), average artifact subtraction (AAS), gradient artifact (GA), carbon-wire loop (CWL), ballistocardiogram (BCG), independent component analysis (ICA). Texts in italics are EEGLAB function names.

For EEG recorded outside the MR scanner (hereafter,*outside-EEG*), we first applied a band-pass filter (0.5–125 Hz; Butterworth filter; 2nd order; applied bi-directionally) and resampled (250 Hz). Subsequent steps were similar to those described inKleinert et al. (2023). Briefly, we applied a band-pass filter (2–20 Hz; FIR filter; hamming window; filter order of 2000; zero-phase non-causal), removed regions larger than a specified size by a spectrum thresholding (using the EEGLAB function*pop_rejcont*with default parameters except for the frequency range of 15–30 Hz), re-referenced data to the common average reference, and removed noisy channels (with default parameters except for omitting a burst criterion). Finally, we rejected artifact components identified using ICLabel (Pion-Tonachini et al., 2019) following ICA with FastICA (Hyvärinen, 1999).

One important judgment to make here was what thresholds to use for ICLabel (hereafter,*ICLabel threshold*). After ICA, ICLabel gives each component the probability of being Brain (brain activity), Muscle (muscle contractions), Eye (eye activity), Heart (ECG artifact), Line noise, Channel noise, or Other. Researchers set thresholds for the seven types for determining whether to keep or reject components. To determine what thresholds to use, we checked all 709 articles citing the original study of ICLabel (Pion-Tonachini et al., 2019) on Google Scholar, identified on June 8, 2023. Of those articles, we could not obtain 114 articles and information about the threshold was missing or unclear in 425 articles (seeSupplementary Table S1). The remaining 170 articles indicated that researchers have taken three general approaches: They set thresholds for 1) artifact components only (i.e., all types but Brain; 136 articles); 2) Brain only (31 articles); or 3) both Brain and artifacts (3 articles). Here, we took the first approach for being a majority. Of the 136 articles, thresholds varied from 0.05 to 1.00 (seeSupplementary Fig. S2), where 0.05 almost always rejected an artifact-type component and 1.00 did not reject it at all. Nonetheless, both means and medians of thresholds for each artifact type fell within a range from 0.60 to 0.80. Also, the thresholds tended to be the same across the artifact-type components within a study (e.g., when 0.60 was set for Muscle, 0.60 was likely to be set for other artifacts as well). Based on these observations, we varied the threshold values from 0.60 to 0.80 in steps of 0.05 while keeping the threshold the same for the six artifact types, thereby resulting in five subsets of data. An exception was made to the Eye component for the oddball data. Possibly due to presenting visual stimuli repeatedly during the task, eye artifacts remained in many participants even at the threshold of 0.60. For this reason, the threshold for the Eye component was kept at 0.30 for the oddball task, which led to a considerable decrease in the number of participants showing eye artifacts.

For EEG recorded inside the MR scanner (hereafter,*inside-EEG*), only those recorded during a PA sequence of fMRI were analyzed in the present study. For such EEG, we first applied a high-pass filter (0.5 Hz; Butterworth filter; 2nd order; applied bi-directionally), AAS for removing GA (Allen et al., 2000), and a low-pass filter (125 Hz; Butterworth filter; 2nd order; applied bi-directionally). Subsequently, we resampled (500 Hz), applied CWL regression for reducing MAs (van der Meer et al., 2016), and again resampled (250 Hz).

We attempted to further attenuate residual BCG artifact using our custom method. Conventional BCG artefact reduction techniques can be broadly categorized into three approaches: temporal (e.g., AAS family), spatial (e.g., ICA family), and hardware (e.g., CWLs; seeAbreu et al., 2022for a review). Our proposed approach falls within the spatial techniques, which are applied to correct residual BCG after temporal/hardware reduction techniques (Abreu et al., 2022). Unlike prior work, which compute ICA and then use heuristics to identify BCG ICs, we propose to use a framework based on joint decorrelation (de Cheveigné & Parra, 2014) to directly estimate spatial components that are specific to residual BCG. Here, the goal of joint decorrelation (JD) is to identify subspaces (i.e., spatial components) that explain most of the BCG artifact variance. To do so, we first extracted ECG R-peak triggers using the fMRIB EEGLAB toolbox (Niazy et al., 2005), and segmented the data into short epochs (offset: -0.2 s, duration: 0.7 s) around the R-peaks forming a tensor$X_{\text{art}}\in {\mathbb{R}}^{C\times E\times T}$withC,E,Treferring to the EEG channels, epochs, and samples within each epoch. Next, N = 25 consecutive epochs were averaged to attenuate ongoing brain activity relative to the residual BCG artifacts. These averages were stacked to form a matrix${\bar{\text{X}}}_{\text{art}}\in {\mathbb{R}}^{C\;\times \;T\;\cdot \;\left\lfloor E/N\right\rfloor }$. We repeated these steps for randomly generated epochs (random triggers) to form another stacked ERP matrix${\bar{\text{X}}}_{\text{ref}}\in {\mathbb{R}}^{C\;\times \;T\;\cdot \;\left\lfloor E/N\right\rfloor }$. To estimate the JD components, spatial covariance matrices${\bar{\text{C}}}_{\text{art}},{\bar{\text{C}}}_{\text{ref}}\in {\mathbb{R}}^{C\times C}$were computed and submitted to generalized eigendecomposition. The generalized eigenvalues$\lambda_{i}$reflect the ratio between the variance explained in${\bar{\text{C}}}_{\text{art}}$and${\bar{\text{C}}}_{\text{ref}}$. To determine a generalizable rejection threshold, we empirically estimated the noise reduction ratio due to averaging across epochs. To do so, we computed the spatial covariance matrix of the reference epochs without averaging (${\text{C}}_{\text{ref}}$) and applied the previously estimated generalized eigenvectors. The average of the elements along the main diagonal was then defined as noise reduction ratio$\bar{\gamma }$. Note that${\bar{\text{C}}}_{\text{ref}}$is transformed to the identity matrix when the generalized eigenvectors are applied. The noise reduction ratio was then used to re-scale the eigenvalues${\tilde{\lambda }}_{i}=(\lambda_{i}-1)\text{​}/\bar{\gamma }+1$. The re-scaled eigenvalues${\tilde{\lambda }}_{i}$reflect the signal-to-noise ratio improvement beyond the noise level reduction due to random averaging.

A threshold (hereafter,*BCG threshold*) was then applied to${\tilde{\lambda }}_{i}$to reject JD components. For interpretations, a threshold of 1.00 could not distinguish variations in EEG due to BCG from other random variations, thereby improperly rejecting a large number of components (approximately 50% of the components by expectation). A threshold of 2.00 meant that the EEG power time-locked to heartbeats had to be twice greater than the average power of other periods (which rarely occurred). We found this method for reducing BCG being an important step for increasing test-retest reliability of microstate metrics. Thus, we varied the BCG threshold from 1.05 (loose) to 1.20 (strict) in steps of 0.01. It should be noted that, even with the same threshold here, results of this BCG reduction method were not deterministic because the reference periods were randomly selected. For this reason, we applied this method multiple time, resulting in 10 replicates for each threshold value. After that, all subsequent steps were identical to those for outside-EEG.

### 2.5 Microstates and test-retest reliability of their metrics

Using outside-EEG on Days 1 and 2, preprocessed as described above, we conducted microstate analyses using the microstate toolbox byKoenig (2017; version 1.2) as described inKleinert et al. (2023). All topography maps, extracted from peaks of global field power (GFP) while ignoring polarity, were sent to a first-level clustering for the identification of 4–6 mean maps for each participant. Here, we used the atomize and agglomerate hierarchical clustering (AAHC;Murray et al., 2008) method for its deterministic property (Poulsen et al., 2018), thereby making the subsequent evaluation simple. The mean individual maps were sent to a second-level clustering to identify a set of grand-mean maps, separately for Days 1 and 2 (These maps will hereafter be referred to as*microstate templates*). We allowed the number of microstate templates to vary from four to six at this stage. Lastly, for automated ordering, the resultant templates were assigned to previously identified templates embedded in the toolbox.

For backfitting, the topography maps were assigned to a microstate template based on their spatial correlation, separately on Days 1 and 2. Only GFP peaks were fit while interpolating those peaks. Temporal smoothing was done with a window size of 20 ms and with non-smoothness penalty of 1. The backfitting resulted in a sequence of microstates for each EEG recording, and four microstate metrics were derived from this sequence for each microstate type (e.g., ms-A).*Duration*is the mean duration of a microstate type in milliseconds.*Occurrence*is the mean occurrences of a microstate type per second.*Coverage*is the percentage of the duration of a microstate type relative to the total duration of all types.*Transition*is the probability of transition from one microstate type of a different type (e.g., ms-A to ms-B). In the case of four templates, for example, each microstate type has three different transition probabilities.

As mentioned above, we varied ICLabel thresholds from 0.60 to 0.80 and also varied the number of microstate templates from four to six. Specifically, we determined parameters of these variables based on the following criteria: 1) Overall similarity of the obtained templates to those reported in previous research; 2) the absence of obvious artifacts (e.g., Eye) at an individual-subject level; and 3) spatial correlation coefficients between a pair of microstate templates of the same type (e.g., ms-A) across Days 1 and 2. The third criterion was included because, in subsequent analyses, we used the same number of microstate templates and the same ICLabel thresholds for comparing a pair of inside- and outside-EEG on each day and, subsequently, for a pair of inside-EEG across Days 1 and 2. Especially for the latter pair, it was considered important to have similar microstate templates for a pair of outside-EEG in the first place; otherwise, templates for inside-EEG would likely be different across Days 1 and 2, thereby decreasing test-retest reliability for that pair.

For inside-EEG, as mentioned above, we varied the BCG threshold from 1.05 and 1.20 (plus the absence of the threshold as a control condition), resulting in 17 subsets of data. For each subset, we computed ICC for test-retest reliability for a pair of inside- and outside-EEG on each day. Then, using the same BCG threshold selected for each day, we computed ICC for a pair of inside-EEG across Days 1 and 2. For additional assessments, we also computed ICC for a pair of outside-EEG on Day 1 and inside-EEG on Day 2 and for a pair of inside-EEG on Day 1 and outside-EEG on Day 2. Consistent with theKleinert et al. (2023)study (alsoAntonova et al., 2022), we considered ICCs smaller than .50, between .50 and .75, between .75 and .90, and larger than .90 poor, moderate, good, and excellent test-retest reliability, respectively. To check the reliability in absolute values of microstate metrics, we selected*absolute agreement*as the definition of ICC, which is more stringent than*consistency*(seeKoo & Li, 2016for a discussion).

It should be noted that, when computing mean coefficients of ICC and of spatial correlation of microstate templates, individual coefficients were z-transformed before averaging. Then, those averages were back-transformed for reporting, being consistent with theKleinert et al. (2023)study. These researchers also reported confidence intervals of the ICC. Yet those intervals were wide (see alsoAntonova et al., 2022) and could never reach a statistical significance given a relatively small sample size in the present study (and our simultaneous EEG-fMRI with CWL likely was one of the biggest datasets available given the high cost). As a remedy, a permutation test with 1000 repetitions, in which a list of participants on one side remained constant while randomly shuffling the order of participants on the other side, was conducted for each mean ICC.

### 2.6 Evaluations of EEG data quality after noise reductions

We examined how the quality of inside-EEG changed across each step of noise reductions using conventional methods for EEG analysis. This consisted of a comparison of EEG traces and PSD across the steps in both resting-state and oddball tasks, and ERP in the oddball task.

## 3 Results

### 3.1 PSD comparison before applying the proposed evaluation method

**Fig. 2.**
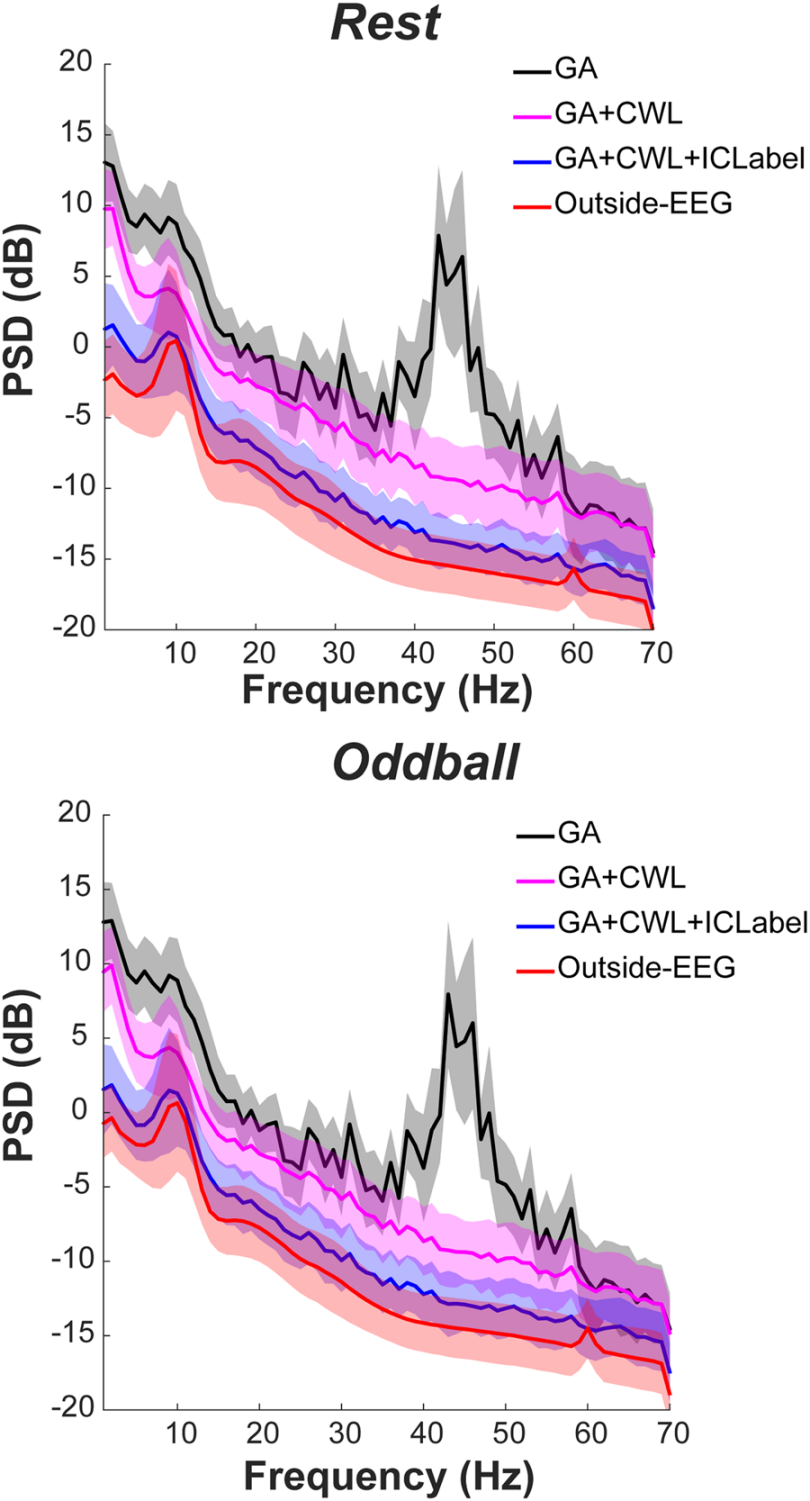
PSD of EEG before applying the proposed evaluation method. PSD across subjects and across recording days was combined separately for resting-state (top) and oddball tasks (bottom). The solid line represents the mean. The band with a lighter color represents +/– 1 standard deviation. GA stands for gradient artifact correction (Step 1-4, 6, 8 inTable 1). GA+CWL stands for a combination of GA correction and CWL regression (Steps 1-6, 8). GA+CWL+ICLabel stands for a combination of GA correction, CWL regression, artifact-component rejections with ICLabel, and other noise-reduction methods (all but Step 7). Steps 1, 3, 6, 8**–**13 were applied to outside-EEG. For these data presentations, a 1**–**70 Hz (instead of 2**–**20 Hz) bandpass filter was applied at Step 8. The slight increase in PSD at 60 Hz for outside-EEG reflects AC current in western Japan where the present experiment was conducted. AC: alternating current; CWL: carbon wire loop; EEG: electroencephalogram; GA: gradient artifact; PSD: power spectrum density

We fist compared the PSD of EEG after GA correction, after CWL regression, and after artifact-component rejections with ICLabel, with outside-EEG as a reference (Fig. 2). We used the ICLabel thresholds determined as described in the next section, and those thresholds were the same between inside- and outside-EEG within each task. The results indicate reductions in noise at each step, being quite similar between resting-state and oddball tasks. For instance, CWL regression reduced noise at a 20–60 Hz range compared to GA correction only. This frequency range likely reflects the helium pump vibration according to our pilot test with a phantom (see the bottom panel inSupplementary Fig. S1). In addition, the CWL regression also reduced power in lower frequencies. Then, artifact-component rejections with ICLabel reduced the overall power, resulting in greater similarity between inside- and outside-EEG in each task, although the power was greater for inside-EEG.

### 3.2 Microstate analysis for a pair of outside-EEG on Days 1 and 2

We applied microstate analyses to outside-EEG on Days 1 and 2 to determine ICLabel thresholds for subsequent analyses. The left panel ofFigure 3shows microstate templates in resting state when the number of templates was four (seeSupplementary Fig. S3for five templates; Data for six templates are omitted for being worse results than the other two). Among these candidates, we selected four microstate templates with ICLabel thresholds of .60 for the following reasons. First, the templates overall were more similar to those reported in previous research (e.g.,Michel & Koenig, 2018). In particular, ms-C and ms-D tended to be more symmetric with four templates (like those reported in the previous research) than ms-C, ms-C’, and ms-D with five templates. This might have resulted from a relatively small number of participants (25 participants) in the present study, compared toKleinert et al. (2023)who extracted five templates from a group of more than 500 participants. Second, among the sets of four templates, the one we selected (ICLabel thresholds of .60) had the highest mean spatial correlation coefficient (.990) between Days 1 and 2. The right panel ofFigure 3shows microstate templates for the oddball task. We selected the .80 threshold with four templates for subsequent analyses for the same reason as the resting-state data.

**Fig. 3.**
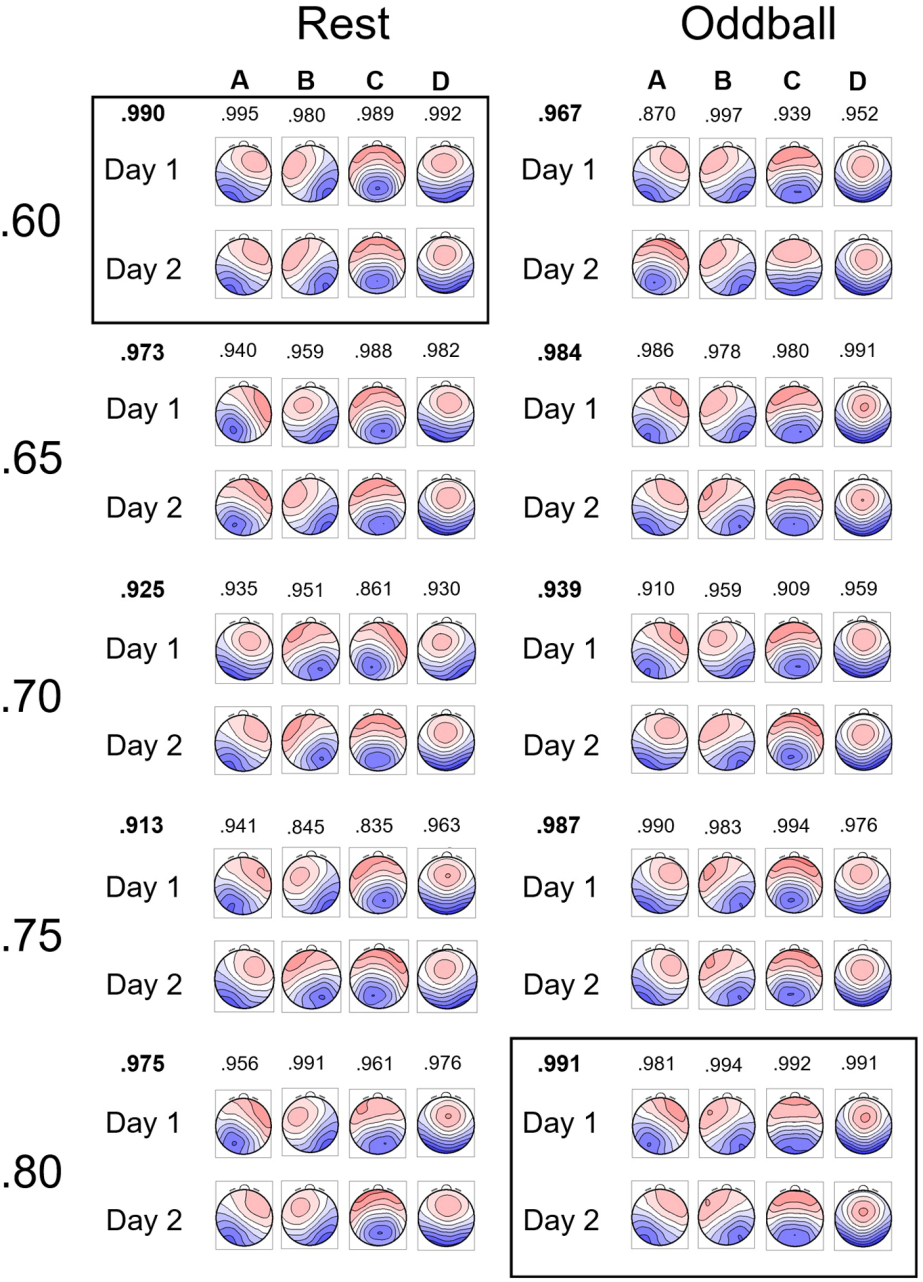
Each set of four microstate templates for outside-EEG in resting-state (left) and oddball tasks (right). The values from .60 to .80 represent ICLabel thresholds. The value above a pair of the same type of template (e.g., A) indicates spatial correlation coefficient, with their mean in bold texts above Day 1. The rectangle indicates the parameters selected for subsequent analyses. EEG: electroencephalogram

Using four templates with ICLabel thresholds determined above,Table 2shows ICCs for each microstate metric. Mean ICCs were .782, .730, .677, and .580 respectively for duration, occurrence, coverage, and transition in resting state. Likewise, respective means were .785, .796, .710, and .506 in oddball. All the means were above the .5 level of ICC (moderately high), also reaching a statistical significance level (*p < *.001) in permutation tests. Moreover, obtaining higher values for duration, occurrence, and coverage relative to transition was consistent with previous studies byAntonova et al. (2022)andKleinert et al. (2023). We also examined ICCs for other ICLabel thresholds unselected above and found that the first three metrics reached the .5 level in many cases, especially, when the mean of spatial correlation coefficients between each pair of microstate template was relatively high (seeSupplementary Table S2). Thus, the finding of moderately high test-retest reliability of duration, occurrence, and coverage was fairly robust.

**Table 2.** Intraclass correlation coefficients of microstate metrics for outside-EEG in resting-state (top) and oddball tasks (bottom).

Resting state				
Microstate	Duration	Occurrence	Coverage	Transition **
ms-A	.665	.789	.587	.767/.367/.560
ms-B	.764	.820	.759	.670/.715/.309
ms-C	.856	.504	.683	.648/.391/.654
ms-D	.805	.732	.659	.478/.615/.593
Mean *	.782	.730	.677	.580

Oddball				
Microstate	Duration	Occurrence	Coverage	Transition **
ms-A	.846	.800	.665	.428/.618/.391
ms-B	.693	.764	.526	.658/.068/.257
ms-C	.864	.760	.889	.763/.765/.615
ms-D	.680	.849	.633	.534/.426/.197
Mean *	.785	.796	.710	.506

* Means were computed by z-transforming the value for each microstate type, averaging them, and then back-transforming the average.*p*< .001 for all the means using a permutation test with 1000 repetitions.

** Each microstate template had three transitions (e.g., ms-A to either ms-B, ms-C, or ms-D), and their ICCs are presented in respective orders.

### 3.3 Microstate analysis for a pair of inside- and outside-EEG on each day

Next, we conducted microstate analyses for a pair of inside- and outside-EEG recorded on the same day. Prior to the analysis, as mentioned above, we reduced BCG artifacts remaining in inside-EEG by varying the threshold for the ratio of EEG power during heartbeats and the power during randomly selected periods as a reference. Given the presence of a random property in this method, we had 10 replicates of inside-EEG after the BCG reduction for each recording day to examine the general effectiveness of this new custom method.

**Fig. 4.**
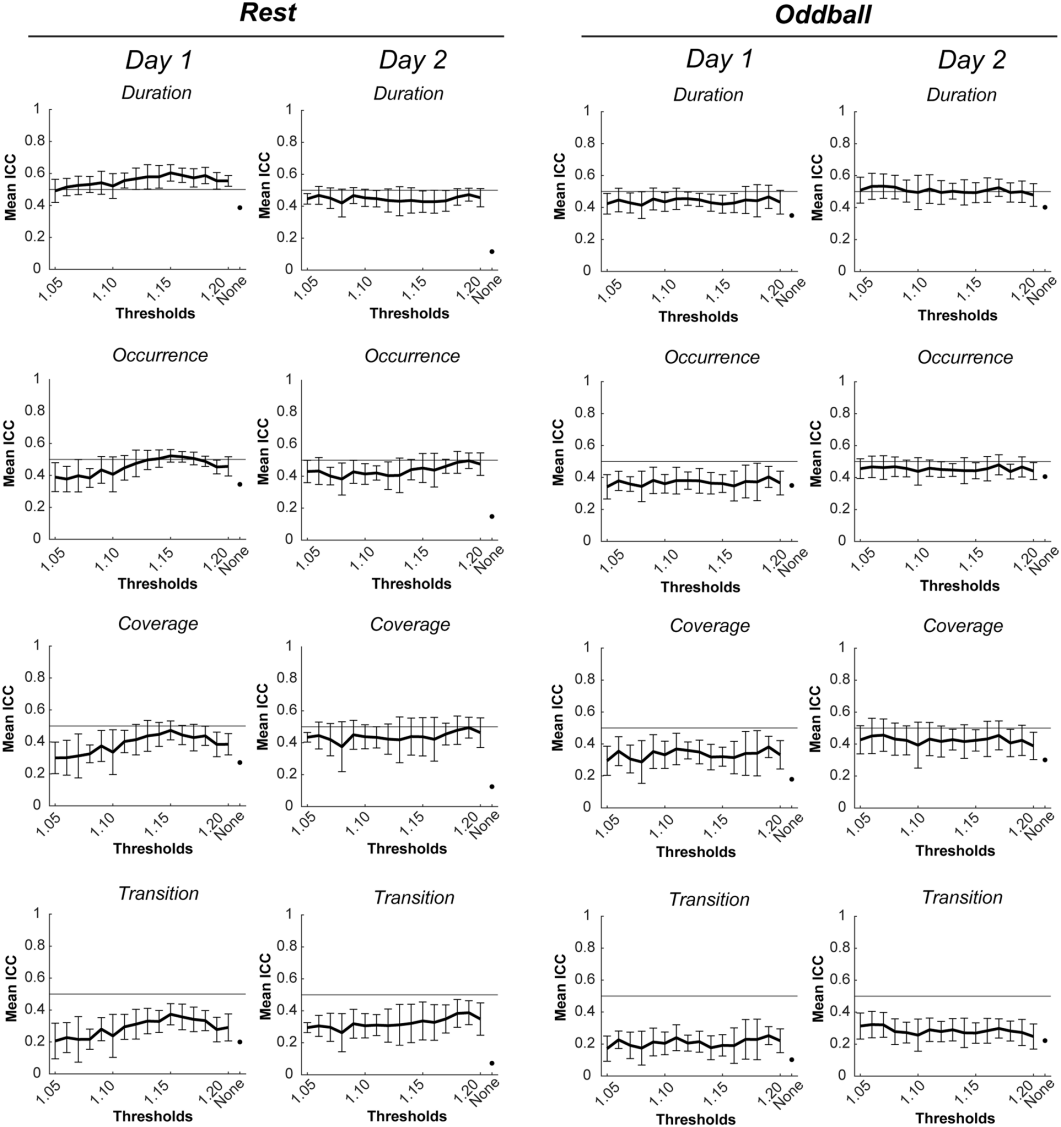
Mean ICCs for each microstate metric for a pair of inside- and outside-EEG, with the inside-EEG being averaged across 10 replicates of the reduction in residual BCG artifacts, as a function of BCG thresholds in resting-state (left) and oddball (right) tasks. Error bars represent +/– 1 standard deviation. “None” corresponds to the absence of the residual BCG artifact reduction step (i.e., Step 7 inTable 1) and it has no error bar for the results being deterministic. A solid horizontal line shows ICC = .5, corresponding to the lower limit of moderately high test-retest reliability. BCG: ballistocardiogram; EEG: electroencephalogram; ICC: interclass correlation coefficient

Figure 4shows mean ICC of each microstate metric for a pair of inside- and outside-EEG, averaged over the 10 replicates, as a function of BCG thresholds in resting-state and oddball tasks. There were a few notable findings. First, regardless of the BCG threshold value, the presence of residual BCG reduction generally increased the mean ICC relative to its absence. Second, the ICCs, at least on average, did not reach the .5 level corresponding to moderately high test-retest reliability in many cases. This, however, does not necessarily mean that the ICC could never reach the .5 level. Indeed, in some of the 10 replicates, the duration, occurrence, and coverage (but not transition) simultaneously reached the .5 level with some BCG threshold values.

**Fig. 5.**
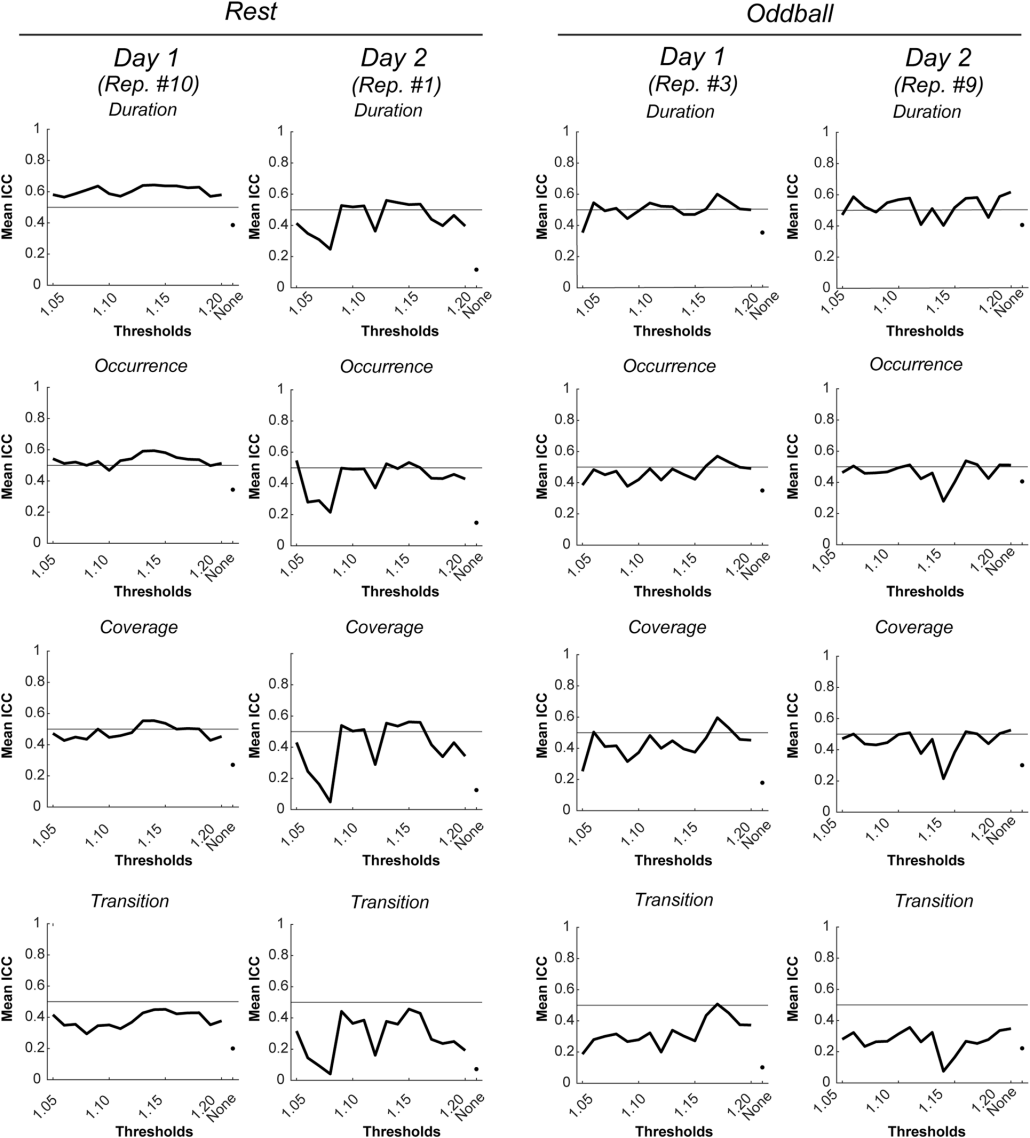
Mean ICCs for each microstate metric for a pair of inside- and outside-EEG, with the inside-EEG being a selected replicate of the reduction in residual BCG artifacts, as a function of BCG thresholds in resting-state (left) and oddball (right) tasks. See the top panel inSupplementary Figure S4showing spatial correlation coefficients for microstate templates extracted from the respective pair of inside- and outside-EEG. BCG: ballistcardiogram; EEG: electroencephalogram; ICC: interclass correlation coefficient.

Figure 5shows examples of this. Selections of the replicates shown in these figures (e.g., replicate #10 for resting state on Day 1) were based on the observation that those three microstate metrics reached the .5 ICC level for a wider and more continuous range of BCG threshold values than other replicates. Those BCG threshold values in these replicates reaching the .5 level served as candidates for subsequent analyses.

It is important to note that such selections of parameters might be considered an example of cherry-picking. Unlike in other contexts, however, this practice was justifiable in the present context. Specifically, we assumed that, if a pair of outside-EEG recorded on two different days has high test-retest reliability in their microstate metrics (which was the case as shown inSection 3.2), a pair of inside- and outside-EEG recorded on the same day should also have high test-retest reliability if MR-induced noise was absent. Thus, it was rather required for us to select parameters that resulted in high test-retest reliability (seeSection 4for more details of the rationale).

### 3.4 Microstate analysis for a pair of inside-EEG on Days 1 and 2

**Fig. 6.**
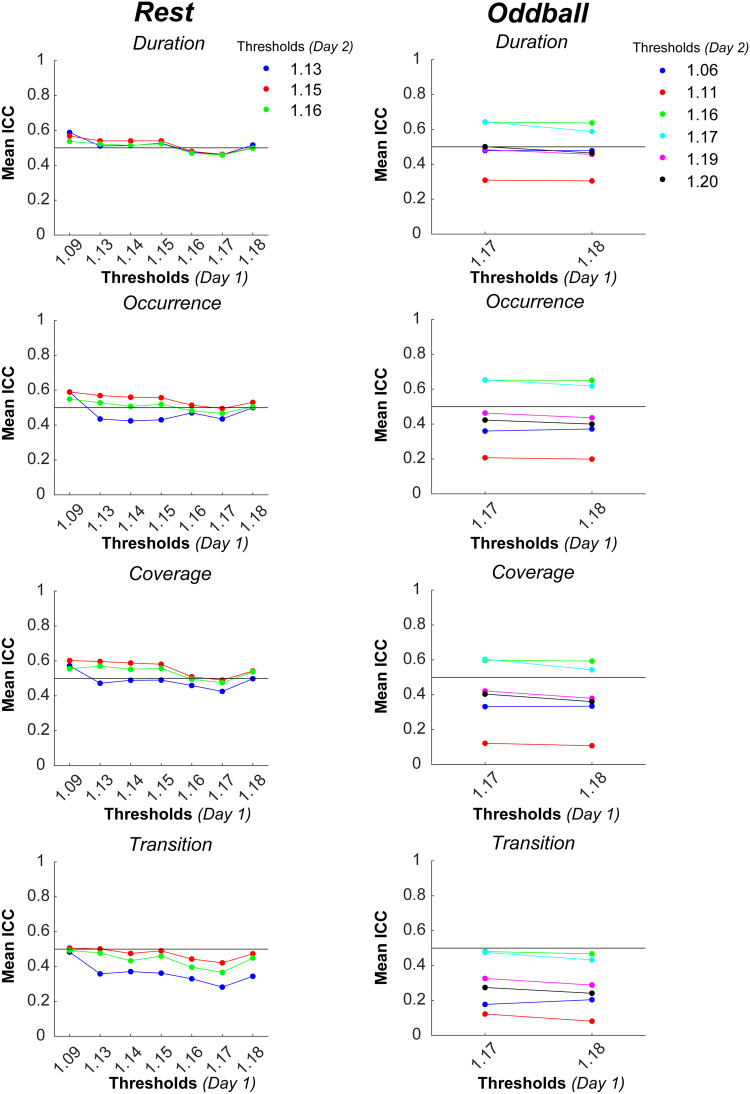
Mean ICCs for each microstate metric for a pair of inside-EEG on Days 1 and 2, with both inside-EEG being a selected replicate of the reduction in residual BCG artifacts, as a function of BCG thresholds on Day 1 in resting-state (left) and oddball (right) tasks. Line with different colors represent BCG thresholds on Day 2. See the bottom panel inSupplementary Figure S4showing spatial correlation coefficients for microstate templates extracted from the respective pair of inside-EEG. BCG: ballistocardiogram; EEG: electroencephalogram

Among the candidates of BCG threshold values identified inSection 3.3, we examined which ones would lead to high test-retest reliability for a pair of inside-EEG on Days 1 and 2.Figure 6shows ICCs of each microstate metric for all possible combinations of the candidate BCG thresholds in each task. For resting state, threshold values of 1.09 on Day 1 with a combination of 1.13 on Day 2 reached the .5 ICC level for duration, occurrence, and coverage but not transition. Likewise, 1.09, 1.13, 1.14, and 1.15 on Day 1 with a combination of 1.15 and also of 1.16 on Day 2 reached the .5 ICC level for the first three metrics. For oddball, 1.17 and 1.18 on Day 1 with a combination of 1.16 and also of 1.17 on Day 2 reached the .5 level for those three metrics. These results indicate that, with some combinations of the candidate BCG thresholds, a pair of inside-EEG recorded on two different days can show moderately high test-retest reliability of microstate metrics except for transition.

**Fig. 7.**
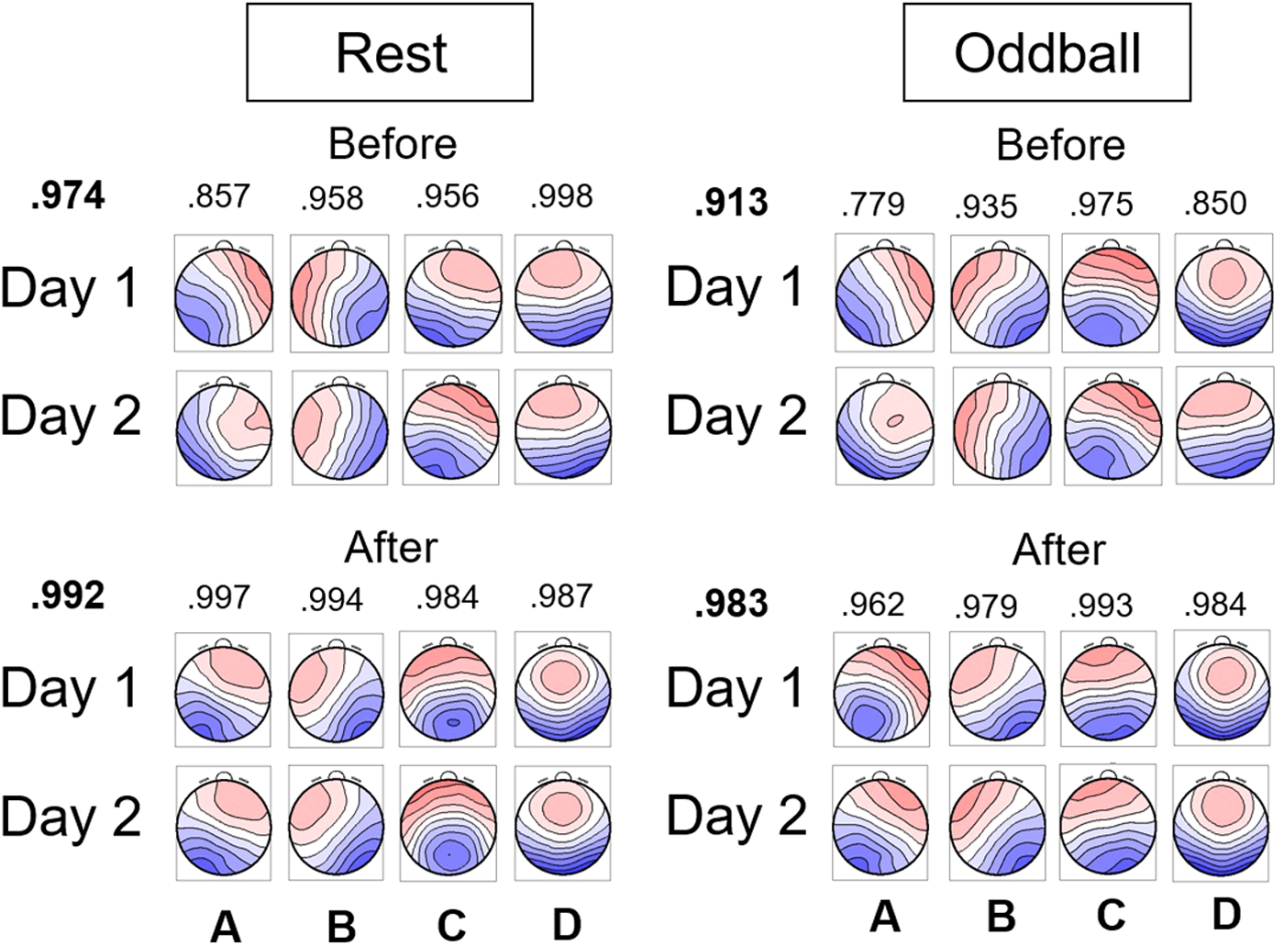
Microstate templates extracted from inside-EEG in resting-state (left) and oddball (right) tasks before and after reducing residual BCG with the final thresholds described in the main text. The value above a pair of the same type of template (e.g., A) indicates spatial correlation coefficient, with their mean in bold texts above Day 1. BCG: ballisotcardiogram; EEG: electroencephalogram

Among the final set of candidates, we selected 1.15 and 1.17 as the final BCG thresholds for resting state and oddball, respectively. Other candidates also were viable but these selections were based on the following considerations. First, it would be ideal to have similar thresholds across different tasks. Second, it would be ideal to have an identical threshold across recording days within a task. This may not occur in other situations, however, because heartbeats change both within and across participants. In that case, similar threshold values should be selected within the task.Figure 7shows microstate templates extracted from inside-EEG in each task before and after reducing residual BCG with the final thresholds. Before the BCG reduction, some templates (B in particular) seemed to be affected by BCG, indicated by vertical contours in the middle. After the reduction, all four types of the template looked more like those for outside-EEG (cf.Fig. 3).

### 3.5 EEG data quality checks after a thorough noise reduction

**Fig. 8.**
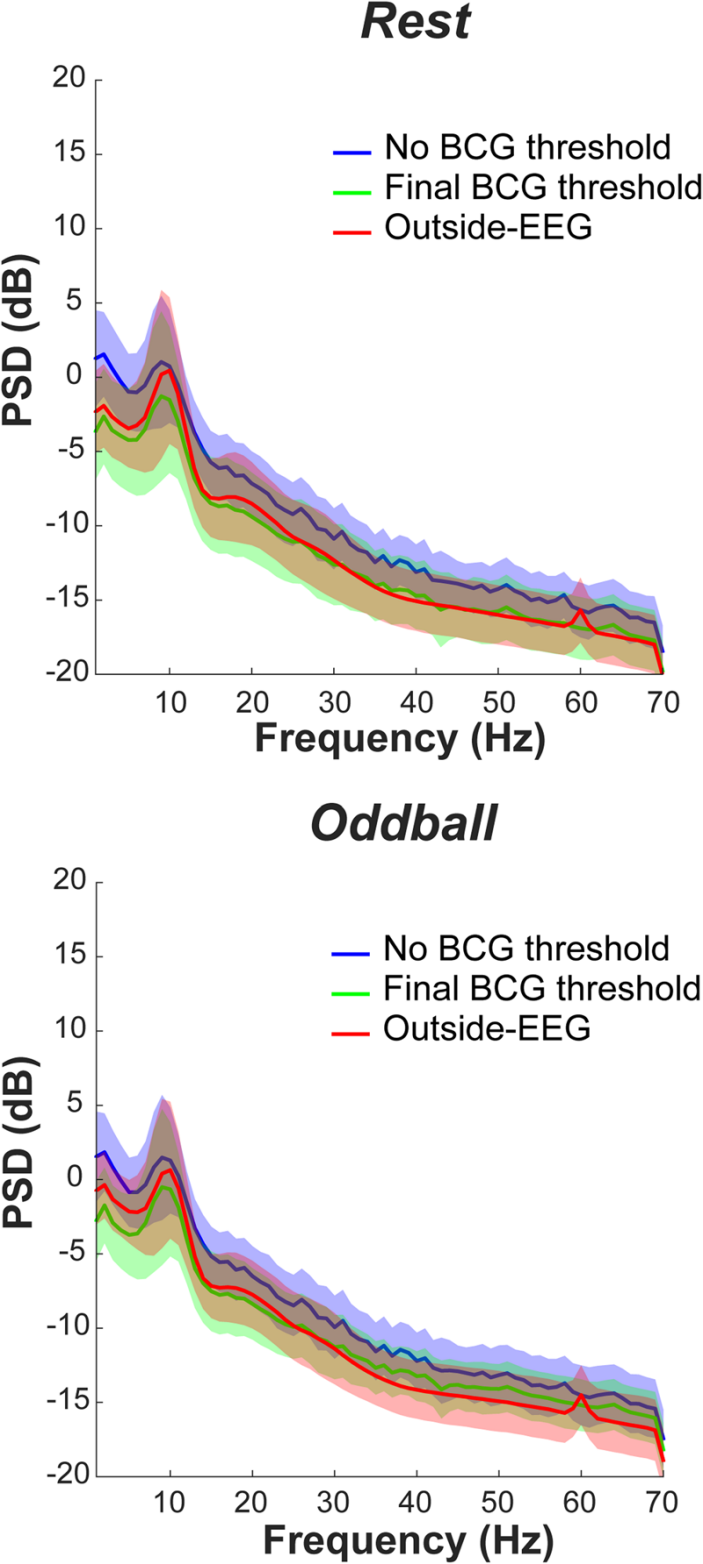
PSD of EEG after reductions in BCG artifacts. PSD across subjects and across recording days was combined separately for resting-state (top) and oddball tasks (bottom). SeeFigure 2for details. BCG: ballistocardiogram; EEG: electroencephalogram; PSD: power spectrum density.

Having determined the BCG threshold in each task, we next examined how reductions in residual BCG changed the quality of inside-EEG.Figure 8shows PSD of inside-EEG with and without the final BCG threshold and outside-EEG as a reference. In both resting-state and oddball tasks, the presence of BCG threshold resulted in greater similarity between inside- and outside-EEG than its absence, in terms of the shape and power. Inside-EEG, however, had slightly lower power than outside-EEG in the 1–25 Hz range.

**Fig. 9.**
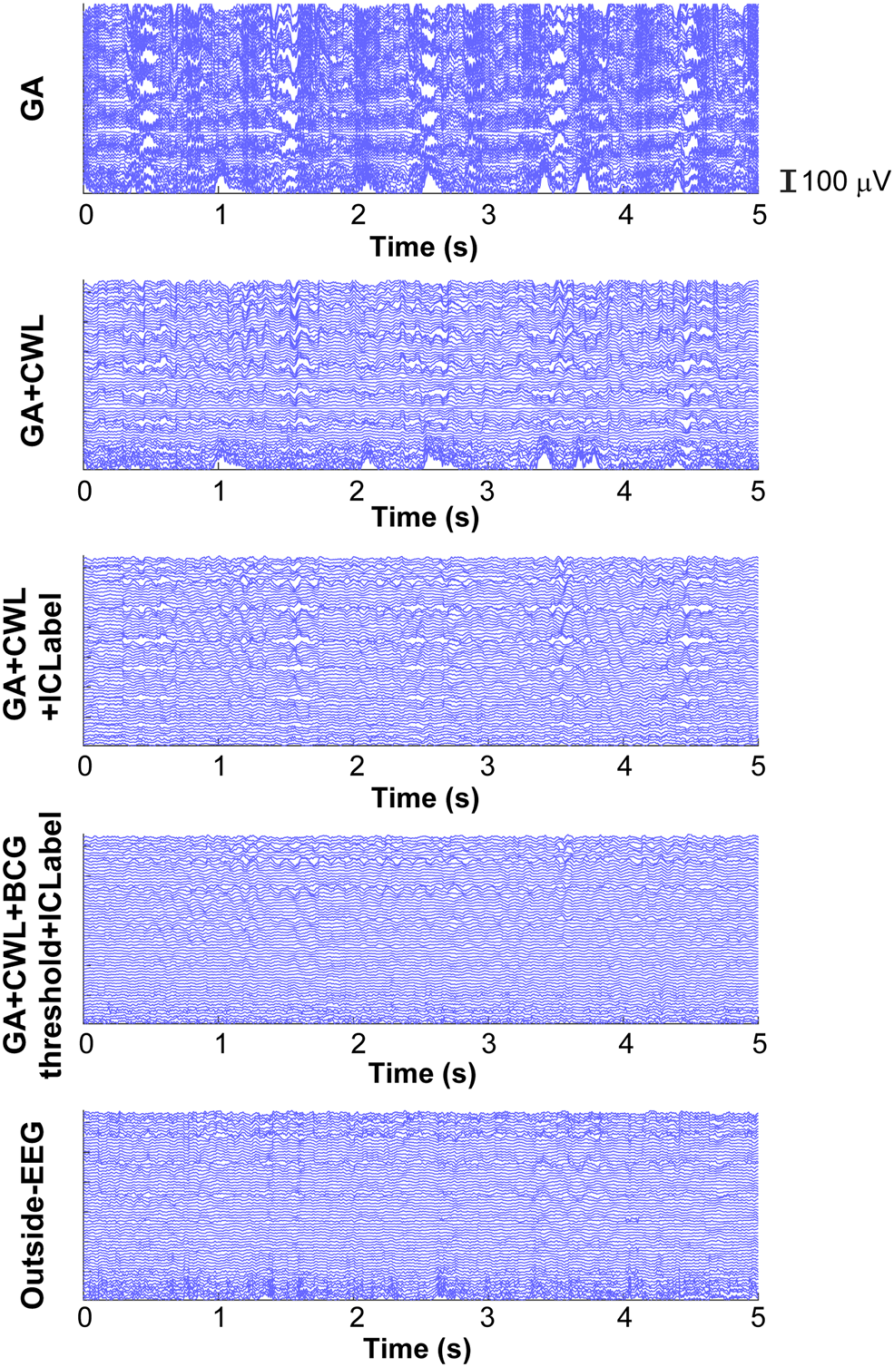
Traces of all EEG channels for a representative participant during the first 5 s period of resting-state task on Day 1. GA, CWL, and ICLabel respectively stand for GA correction, CWL regression, and artifact-component rejection with ICLabel. BCG threshold represents the final BCG threshold identified inSection 3.4. Noise was reduced for outside-EEG as described inTable 1. BCG: ballistocardiogram; CWL: carbon wire loop; EEG: electroencephalogram; GA: gradient artifact.

Figure 9shows representative traces of all EEG channels for a single participant across different noise-reduction methods. The top four panels were generated from an identical raw inside-EEG data, whereas the bottom panel corresponds to outside-EEG of the same participant. Compared to GA correction only, its combination with CWL regression considerably reduced noise. Artifact-component rejections with ICLabel also reduced noise. Moreover, our custom BCG correction made the traces look more like outside-EEG.

**Fig. 10.**
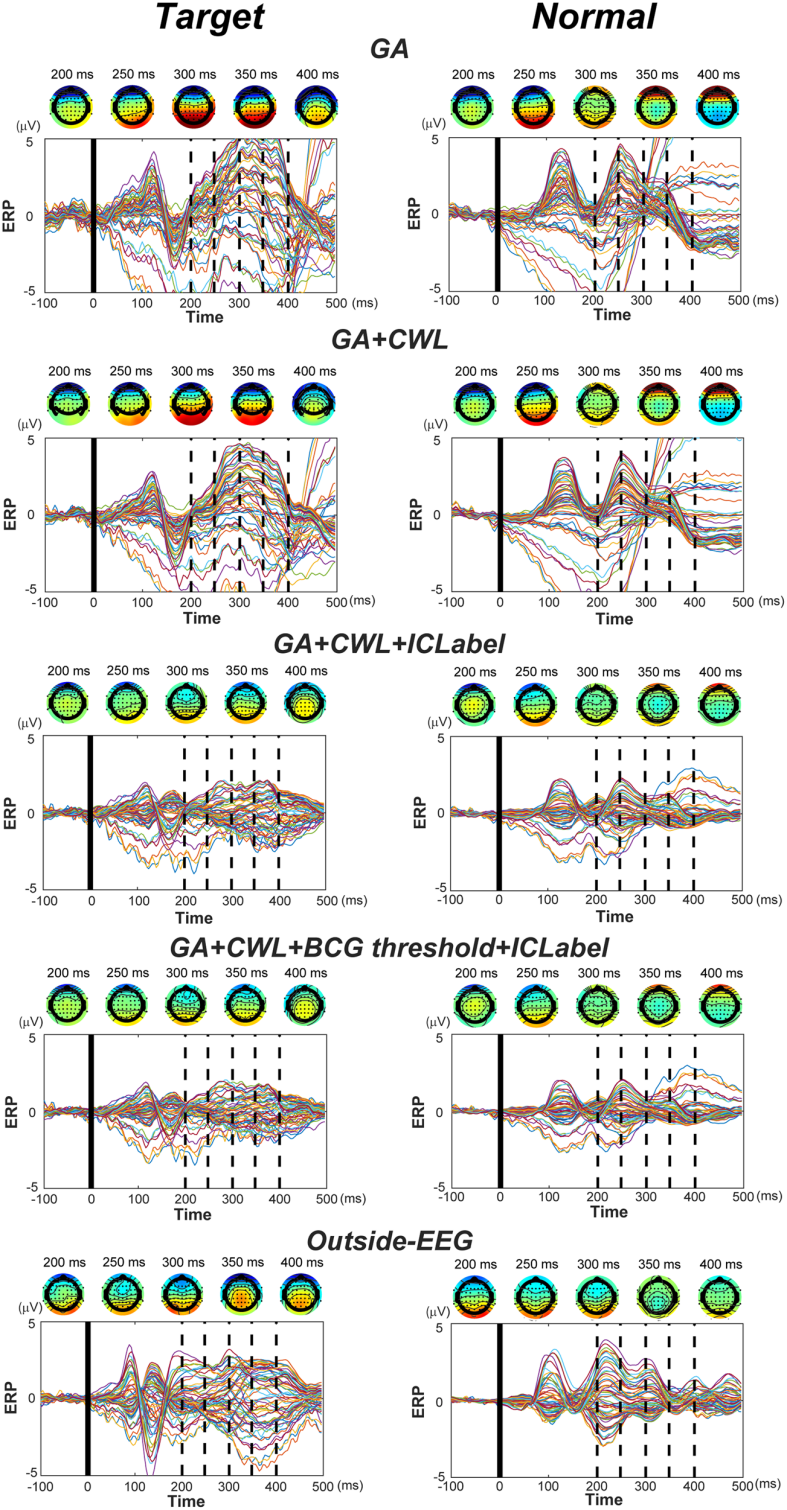
ERPs in the oddball task. Thick vertical lines represent the onset of target (left) or normal (right) stimuli, detected by photo sensors. Dashed vertical lines indicate 200–400 ms in steps of 50 ms after the stimulus onset, corresponding to topography maps above each graph. Lines with different colors represent electrodes, each referenced to the mean microvolt between -100 and 0 ms. GA, CWL, and ICLabel respectively stand for GA correction, CWL regression, and artifact-component rejection with ICLabel. BCG threshold represents the final BCG threshold identified inSection 3.4. Noise was reduced for outside-EEG as described in the text. BCG: ballistocardiogram; CWL: carbon wire loop; ERP: event-related potential; GA: gradient artifact.

We next checked ERPs in the oddball task.Figure 10shows mean ERPs of inside-EEG, averaged over subjects, across each step of noise reduction (seeSupplementary Fig. S5for a more detailed time-frequency analysis of ERPs). For outside-EEG (bottom panels), a comparison between trials with target and normal stimuli clearly indicates physiologically meaningful signals such as P300. Moreover, P300 was associated with posterior electrodes as indicated by topography maps between 350 and 400 ms after the stimulus onset. For inside-EEG compared to outside-EEG, the amplitude was bigger after GA correction only, and in its combination with CWL regression as well, indicating the presence of noise. The noise considerably disappeared after artifact-component rejections with ICLabel while keeping the physiological signals. The amplitude of these signals already was smaller for inside-EEG in this step compared to outside-EEG. This was a noteworthy finding because the two were supposed to be equivalent except for the presence of residual BCG. Thus, these results suggest the possibility of excessive reduction in the amplitude as a consequence of GA correction, CWL regression, or both. This, in turn, may have contributed to lower PSD for inside-EEG relative to outside-EEG (seeFig. 8). In addition, a close observation of ERPs indicates that the onset of physiological signals was slightly delayed compared to outside-EEG (seeSection 4for details). Finally, the physiological signals remained, though somewhat attenuated, after reducing residual BCG. The last result indicates that reduction in the BCG itself was not excessive.

## 4 Discussion

We introduced a new evaluation method for noise reduction in simultaneous EEG-fMRI settings. Given the absence of ground truth in EEG, our method relied on high test-retest reliability of microstate metrics (duration, occurrence, and coverage in particular) commonly reported in the literature (Antonova et al., 2022;Khanna et al., 2014;Kleinert et al., 2023;Liu et al., 2020; cf.Popov et al., 2023). Specifically, our first step was to confirm the high reliability in a pair of EEG recorded outside an MR scanner across two different days. Subsequently, we assumed that the reliability also was supposed to be high as well for a pair of EEG recorded inside and outside the scanner on the same day if MR-induced noise was absent. Accordingly, we attempted to identify noise-reduction parameters (e.g., the threshold for residual BCG in the present case) in a way that the reliability would increase for that pair of EEG. Next, we attempted to identify the BCG threshold that would also lead to high reliability for a pair of EEG recorded inside the scanner across the two different days. We found that BCG thresholds of 1.15 and 1.17 (for resting-state and oddball tasks respectively) resulted in moderately high reliability for the latter two pairs of EEG in addition to the first pair. Achieving moderately high reliability in EEG recorded inside the scanner suggests that this EEG approached the quality of EEG recorded outside the scanner. This, in turn, leads to a better analysis of EEG in relation to fMRI.

Previous research on noise reduction in simultaneous EEG-fMRI has made considerable success in developing hardware- or software-based methods for reducing scanning noise. This includes GA correction with AAS (Allen et al., 2000), BCG reduction with OBS (Debener et al., 2007), and BCG plus MA reductions either with RLAS (Chowdhury et al., 2014) or CWL (van der Meer et al., 2016). In contrast, evaluation methods for the noise reduction had remained rather limited. Methods such as eye open/closed (van der Meer et al., 2016), steady-state visual evoked potential (Levitt et al., 2023), and ERP (Abreu et al., 2016) are useful for examining the presence of physiologically meaningful signals after noise reduction. Nonetheless, this approach would only indicate that the reduction is not excessive but not that it is sufficient. Moreover, its applicability is limited in some way (e.g., steady-state visual evoked potential cannot be used for resting-state EEG). The use of outside-EEG as the ground truth by proxy appears to be the only way for evaluating reductions in noise associated with MR scanners. For instance,van der Meer et al. (2016)compared PSD between inside- and outside-EEG and measured the normalized Euclidean distance between the two. Such a measure was sensitive enough for detecting changes in big noise like GA and MA but PSD does not capture dynamics of brain activity for the temporal information being collapsed. Moreover, the use of outside-EEG as the ground truth by proxy is based on an implicit assumption of high test-retest reliability but it has not been a common practice to test this assumption in the context of simultaneous EEG-fMRI.

Given these backgrounds, we sought a new evaluation method to overcome the above issues. In the present study, we selected EEG microstate as a basis for the evaluation method for several reasons. First, obtaining microstates does not require any stimulus change or demand on participants (e.g., resting state), thereby having high applicability. Second, microstates can cover a different range of frequency. We used a 2–20 Hz range in the present study but wider ranges are viable options as well (Michel & Koenig, 2018). Third, microstate metrics capture dynamics of brain activity. And lastly, their test-retest reliability has been reported high for outside-EEG across several different studies (Antonova et al., 2022;Khanna et al., 2014;Kleinert et al., 2023;Liu et al., 2020) and this was corroborated in the present study as well. Yet EEG microstate may not be the only viable option and other EEG features might be found useful in future. Thus, our evaluation method should rather be considered a general approach without the necessity of relying on EEG microstate.

Another contribution of the present study was that we extended previous findings of the high test-retest reliability of microstate metrics (Antonova et al., 2022;Khanna et al., 2014;Kleinert et al., 2023;Liu et al., 2020) to a visual oddball task. It has been common to use resting-state EEG data (either eyes open, eyes closed, or both) in these previous studies. The Antonova et al. study was an exception in that they also used tasks including mind-wandering (which was similar to resting state), silent vocalization, and visual imagination. In the present study, we used the oddball task in which visual stimuli were presented repeatedly and participants were instructed to report how many target stimuli were presented. Despite the manipulation of environmental stimuli and the demanding task, microstate metrics achieved moderately high test-retest reliability for a pair of outside-EEG, a pair of inside- and outside-EEG, and a pair of inside-EEG, indicating the robustness of the previous findings. The robustness, in turn, supports the applicability of the present evaluation method for noise reduction across different experimental tasks.

The present study has several limitations. First, we have not yet established a series of noise-reduction methods that makes the quality of inside-EEG equivalent to outside-EEG. Compared to a pair of outside-EEG across two days, ICCs consistently were lower in a pair of inside- and outside-EEG on the same day. One potential factor lowering the ICC was the difference in recording environments (i.e., shield room vs. MR scanner). Nonetheless, the lower ICC in a pair of inside-EEG, where the recording environment was identical (perhaps, with an exception of magnetic field, which could have been affected by the location of objects placed in the MR scanner bore), suggests the presence of other contributing factors. Thus, there appears to be a room for further improvement in noise-reduction methods. We speculate the presence of such a room in GA correction, CWL regression, and/or residual BCG reduction because all the subsequent steps for noise reduction were identical for inside- and outside-EEG within an experimental task.

Another limitation was that our selection of BCG thresholds was based on their resultant ICCs. This could be an example of cherry-picking but it was a justifiable practice in the present context for two reasons. First, our goal was not to identify BCG thresholds that were generalizable to other datasets. Rather, the goal was to obtain inside-EEG with a quality equivalent to outside-EEG in a specific dataset. Achieving the latter goal would be a prerequisite for an analysis of inside-EEG in relation to fMRI. Second, our approach was based on an assumption that test-retest reliability was supposed to be high for a pair of inside- and outside-EEG recorded on the same day if MR-induced noise was absent, because it was high for a pair of outside-EEG recorded on two different days. Thus, we had to select BCG thresholds in a way ICCs would increase. This was a sound assumption because of the temporal proximity between two recordings in the former case compared to the latter case. To empirically support the assumption,Kleinert et al. (2023)found little difference between short- and long-term test-retest reliability of microstate metrics, where average intervals between two recordings were 99–138 min and 63 days for the short and long terms, respectively. To reiterate the second point in a more general language, we started with confirming that test-retest reliability was high for a pair of outside-EEG recorded on two different days. This naturally led to the notion that reliability likely was high for a pair of inside- and outside-EEG on the same day if MR-induced noise was absent despite that it was not actually measured. This was the assumption underlying the present evaluation method backing up our cherry-picking practice, and we believe it was a reasonable assumption. For these reasons, our practice of selecting BCG thresholds based on their resultant ICCs was justifiable although it would be more ideal if this step can be skipped. Another related limitation was the presence of large variability in ICCs across replicates of residual BCG reduction. This was due to having a random property in our custom method for reducing residual BCG. The method might be improved by adjusting some parameters (e.g., the size of intervals randomly selected as a reference EEG power).

Other limitations are rather practical. One disadvantage of the present evaluation method is the necessity of having at least two EEG recordings inside and outside an MR scanner each (i.e., four recordings total). This is an inevitable consequence of relying on test-retest reliability. As a tradeoff, however, there is little space for making subjective judgments when selecting noise-reduction parameters, thereby promoting a good practice in science. A potential variant of the present evaluation method without the necessity of having participants come to the study twice is to obtain outside-EEG, inside-EEG, another inside-EEG, and another outside-EEG in that order on the same day. If test-retest reliability is high for a pair of the first and the second outside-EEG, researchers can reasonably assume that reliability also be high for a pair of inside-EEG as well as for a pair of inside- and outside-EEG. There could be a tradeoff, however, with other potential factors such as fatigue. The other practical issue is that the applicability of the present evaluation method is limited to offline data analyses. It takes a considerable amount of time to extract microstate templates and compute their metrics, making online data analyses difficult. Yet, once relevant parameters (e.g., BCG thresholds) are found generalizable across different datasets with the accumulation of simultaneous EEG-fMRI data in future, it will not take much time to apply a series of noise reductions. Thus, online noise reductions may become possible.

In addition to the above limitations, caution is advised in the use of outside-EEG as it could also be polluted in some shield rooms. In our case, 60-Hz line noise from a power outlet was the only apparent artifact when “EEG” was measured with a phantom in the shield room (see the top panel inSupplementary Fig. S1). This was not an issue, however, for the line noise being outside the target frequency range of microstate (i.e., 2–20 Hz). It is important to check EEG quality in the shield room before taking the present evaluation approach due to its heavy reliance on the cleanness of outside-EEG.

Aside from the topic of noise reduction, one peculiar finding of the present study was the delayed onset of ERP for inside-EEG compared to outside-EEG in the oddball task. We checked all the devices and found that the difference in delay did not result from any factors related to the device. Thus, it most likely resulted from the difference in situation between inside and outside the MR scanner apart from MR-induced noise (body position, looking visual stimuli through a small mirror, etc.). We identified few studies in the literature relevant to this issue. A more thorough analysis is underway and we will report the result in future. For now, the delayed onset of ERP raises the importance of controlling recording environments/settings as much as possible for a more direct comparison of inside- and outside-EEG.

Lastly, we checked the quality of fMRI recorded during resting-state and oddball tasks. Brain networks identified from the resting-state data (Supplementary Figs. S6 and S7) and results of a GLM analysis of BOLD signals in oddball (Supplementary Fig. S8) were similar to those reported in previous research (Huang et al., 2005;Smith et al., 2009). The consistency between the literature and our dataset indicates that BOLD signals in our simultaneous EEG-fMRI dataset were not idiosyncratic. This indirectly supports the validity of our EEG data for being recorded with fMRI simultaneously. Supports for the data validity are relevant here given that our EEG analyses primarily relied on test-retest reliability.

## 5 Conclusion

We introduced a novel evaluation method for noise reduction in simultaneous EEG-fMRI taking advantage of high test-retest reliability of EEG microstate metrics. After applying a series of noise reductions, the reliability was moderately high for a pair of outside-EEG recorded on two different days, for a pair of inside- and outside-EEG on the same day, and for a pair of inside-EEG across the two different days. However, the reliability was lower for the last two pairs than the first pair, suggesting a room for further improvement in noise reduction. Improving the quality of inside-EEG would promote not only better understanding of how a common brain activity manifests in two different modalities (EEG and fMRI) but also the technology of neurofeedback having a high temporal resolution of EEG together with a high spatial resolution of fMRI.

## Data and Code Availability

We are planning to make the code and dataset publicly available after the end of the project (March 2026) funded as described below. Python and MATLAB versions of the joint-decorrelation function for reducing residual BCG artifacts described in the main text are publicly available from the following link:https://github.com/atr-iine/joint_decorrelation_for_phase-locked_artifact_attenuation.

## Author Contributions

Toshikazu Kuroda, Reinmar J. Kobler, and Takeshi Ogawa. contributed to the study conception and design under the supervision of Motoaki Kawanabe. Material preparation and data collection were performed by Toshikazu Kuroda,Takeshi Ogawa, Mizuki Tsutsumi, and Tomohiko Kishi. Toshikazu Kuroda conducted data analyses, and Reinmar J. Kobler developed the joint decorrelation method. Mizuki Tsutsumi and Tomohiko Kishi conducted a literature review for the ICLabel pre-trained classifier. The draft of the manuscript was written by Toshikazu Kuroda, Reinmar J. Kobler, and Takeshi Ogawa. All authors read and approved the final manuscript.

## Funding

This work was supported by Innovative Science and Technology Initiative for Security Grant Number JPJ004596, ATLA, Japan.

## Declaration of Competing Interest

We have no conflict of interest.

## Supplementary Material

Supplementary Material
