# Supplementary material for: Test-retest reliability of EEG microstate metrics for evaluating noise reductions in simultaneous EEG-fMRI
Source: Imaging Neurosci (Camb). 2024 Aug 29;2:imag-2-00272. doi: 10.1162/imag_a_00272 (PMC12290594; doi:10.1162/imag_a_00272)
Supplement: Supplementary Material [file imag_a_00272-supp.pdf]

Supplementary Materials

Supplementary Table S1. A list of published papers referencing to the original paper on the ICLabel pre-trained classifier (Pion-Tonachini et al., 2019).

2019

Index	Author	Year	Journal information	muscle	eye	heart	line noise	channel noise	other	brain	brain removed
1	Chang, C. Y., Hsu, S. H., Pion-Tonachini, L., & Jung, T. P.	2019	Evaluation of artifact subspace reconstruction for automatic artifact components removal in multi-channel EEG recordings. IEEE Transactions on Biomedical Engineering, 67(4), 1114-1121. https://doi.org/10.1109/TBME.2019.2930186								
2	Ladouce, S., Donaldson, D. I., Dudchenko, P. A., & Ietswaart, M.	2019	Mobile EEG identifies the re-allocation of attention during real-world activity. Scientific reports, 9(1), 1-10. https://doi.org/10.1038/s41598-019-51996-y								
3	Pion-Tonachini, L., Kreutz-Delgado, K., & Makeig, S.	2019	The ICLabel dataset of electroencephalographic (EEG) independent component (IC) features. Data in brief, 25, 104101. https://doi.org/10.1016/j.dib.2019.104101								
4	Plechawska-Wójcik, M., Tokovarov, M., Kaczorowska, M., & Zapała, D.	2019	A three-class classification of cognitive workload based on EEG spectral data. Applied Sciences, 9(24), 5340. https://doi.org/10.3390/app9245340								
5	Marini, F., Lee, C., Wagner, J., Makeig, S., & Gola, M.	2019	A comparative evaluation of signal quality between a research-grade and a wireless dry-electrode mobile EEG system. Journal of neural engineering, 16(5), 054001. https://doi.org/10.1088/1741-2552/ab21f2								
6	Sasaki, M., Iversen, J., & Callan, D. E.	2019	Music improvisation is characterized by increase EEG spectral power in prefrontal and perceptual motor cortical sources and can be reliably classified from non-improvisatory performance. Frontiers in Human Neuroscience, 13, 435. https://doi.org/10.3389/fnhum.2019.00435							0.6	
7	Jończyk, R., Korolczuk, I., Balatsou, E., & Thierry, G.	2019	Keep calm and carry on: electrophysiological evaluation of emotional anticipation in the second language. Social Cognitive and Affective Neuroscience, 14(8), 885-898. https://doi.org/10.1093/scan/nsz066								

8	Mazurek, K. A., Richardson, D., Abraham, N., Foxe, J. J., & Freedman, E. G.	2019	Utilizing high-density electroencephalography and motion capture technology to characterize sensorimotor integration while performing complex actions. IEEE Transactions on Neural Systems and Rehabilitation Engineering, 28(1), 287-296. https://doi.org/10.1109/TNSRE.2019.2941574								
9	Brown, T., McConnell, M., Rupp, G., Meghdadi, A., Richard, C., Schmitt, R., ... & Berka, C.	2019	Correlation of EEG biomarkers of cannabis with measured driving impairment. Traffic injury prevention, 20(sup2), S148-S151. https://doi.org/10.1080/15389588.2019.1662256								
10	Oxner, M., Rosentreter, E. T., Hayward, W. G., & Corballis, P. M.	2019	Prediction errors in surface segmentation are reflected in the visual mismatch negativity, independently of task and surface features. Journal of Vision, 19(6), 9-9. https://doi.org/10.1167/19.6.9								
11	Allexandre, D., Hoxha, A., Handiru, V. S., Saleh, S., Selvan, S. E., & Yue, G. H.	2019	Altered Cortical and Postural Response to Balance Perturbation in Traumatic Brain Injury–An EEG Pilot Study. In 2019 41st Annual International Conference of the IEEE Engineering in Medicine and Biology Society (EMBC) (pp. 1543-1546). IEEE. https://doi.org/10.1109/EMBC.2019.8856645							0.85	
12	Marini, F., Breeding, K. A., & Snow, J. C.	2019	Dataset of 24-subject EEG recordings during viewing of real-world objects and planar images of the same items. Data in brief, 24, 103857. https://doi.org/10.1016/j.dib.2019.103857								
13	Nikolaev, A. R., Ehinger, B. V., Meghanathan, R. N., & van Leeuwen, C.	2019	At a second glance: cognitive and oculomotor neural activity of refixation planning. bioRxiv, 660308. https://doi.org/10.1101/660308	0.4	0.9	0.5	0.4	0.4	0.4		
14	Tran, X. A.	2019	Neural Connectivity in Infants at Familial Risk for Autism Spectrum Disorder. University of California, Los Angeles.								
15	Krol, L. R., Pawlitzki, J., Mousavi, M., Andreessen, L. M., & Zander, T. O.	2019	Salience versus Valence in Implicit Cursor Control: First Indications of Separate Cortical Processes. In 2019 IEEE International Conference on Systems, Man and Cybernetics (SMC) (pp. 3913-3918). IEEE. https://doi.org/10.1109/SMC.2019.8913936								
16	Arnau, S., Löffler, C., Rummel, J., Hagemann, D., Wascher, E., & Schubert, A. L.	2019	The Electrophysiological Signature of Mind Wandering. bioRxiv, 819805. https://doi.org/10.1101/819805							0.5	

17	Mariola, A., Baykova, R., Chang, A. Y., Seth, A. K., & Roseboom, W.	2019	Clear Evidence for Electrophysiological Signatures of Duration and Rhythm Prediction, but not across Sensory Modalities. In 2019 Conference on Cognitive Computational Neuroscience. Berlin, Germany: Cognitive Computational Neuroscience.								
18	Mayeli, A.	2019	Advancing Multimodal Approaches to Study Human Brain: Improvements in Simultaneous EEG-fMRI Acquisition. https://hdl.handle.net/11244/323236								
19	Pion-Tonachini, L., Kreutz-Delgado, K., & Makeig, S.	2019	Furthering the Automation of Electroencephalographic Source Analysis. University of California, San Diego.								

2020

Index	Author	Year	Journal information	muscle	eye	heart	line noise	channel noise	other	brain	brain removed
1	Ismail, L. E., & Karwowski, W.	2020	A graph theory-based modeling of functional brain connectivity based on eeg: A systematic review in the context of neuroergonomics. IEEE Access, 8, 155103-155135. https://doi.org/10.1109/ACCESS.2020.3018995								
2	Doma, V., & Pirouz, M.	2020	A comparative analysis of machine learning methods for emotion recognition using EEG and peripheral physiological signals. Journal of Big Data, 7(1), 1-21. https://doi.org/10.1186/s40537-020-00289-7								
3	Arnau, S., Löffler, C., Rummel, J., Hagemann, D., Wascher, E., & Schubert, A. L.	2020	Inter-trial alpha power indicates mind wandering. Psychophysiology, 57(6), e13581. https://doi.org/10.1111/psyp.13581							0.5	
4	Shah, S. A. A., Zhang, L., & Bais, A.	2020	Dynamical system based compact deep hybrid network for classification of Parkinson disease related EEG signals. Neural Networks, 130, 75-84. https://doi.org/10.1016/j.neunet.2020.06.018								
5	Koshiyama, D., Miyakoshi, M., Joshi, Y. B., Molina, J. L., Tanaka-Koshiyama, K., Sprock, J., ... & Light, G. A.	2020	A distributed frontotemporal network underlies gamma-band synchronization impairments in schizophrenia patients. Neuropsychopharmacology, 45(13), 2198-2206. https://doi.org/10.1038/s41386-020-00806-5							0.7	
6	Leach, S. C., Morales, S., Bowers, M. E.,	2020	Adjusting ADJUST: Optimizing the ADJUST algorithm for pediatric data using geodesic nets. Psychophysiology, 57(8), e13566. https://doi.org/10.1111/psyp.13566								

	Buzzell, G. A., Debnath, R., Beall, D., & Fox, N. A.										
7	Tanaka-Koshiyama, K., Koshiyama, D., Miyakoshi, M., Joshi, Y. B., Molina, J. L., Sprock, J., ... & Light, G. A.	2020	Abnormal spontaneous gamma power is associated with verbal learning and memory dysfunction in schizophrenia. Frontiers in Psychiatry, 11, 832. https://doi.org/10.3389/fpsyt.2020.00832							0.7	
8	Kobler, R. J., Sburlea, A. I., Lopes-Dias, C., Schwarz, A., Hirata, M., & Müller-Putz, G. R.	2020	Corneo-retinal-dipole and eyelid-related eye artifacts can be corrected offline and online in electroencephalographic and magnetoencephalographic signals. NeuroImage, 218, 117000. https://doi.org/10.1016/j.neuroimage.2020.117000								
9	Laureanti, R., Bilucaglia, M., Zito, M., Circi, R., Fici, A., Rivetti, F., ... & Russo, V.	2020	Emotion assessment using Machine Learning and low-cost wearable devices. In 2020 42nd Annual International Conference of the IEEE Engineering in Medicine & Biology Society (EMBC) (pp. 576-579). IEEE. https://doi.org/10.1109/EMBC44109.2020.9175221								0.7
10	Bonmassar, C., Widmann, A., & Wetzel, N.	2020	The impact of novelty and emotion on attention-related neuronal and pupil responses in children. Developmental Cognitive Neuroscience, 42, 100766. https://doi.org/10.1016/j.dcn.2020.100766								
11	Chatzichristos, C., Dan, J., Narayanan, A. M., Seeuws, N., Vandecasteele, K., De Vos, M., ... & Van Huffel, S.	2020	Epileptic seizure detection in EEG via fusion of multi-view attention-gated U-net deep neural networks. In 2020 IEEE Signal Processing in Medicine and Biology Symposium (SPMB) (pp. 1-7). IEEE. https://doi.org/10.1109/SPMB50085.2020.9353630								
12	Dercksen, T. T., Widmann, A., Schröger, E., & Wetzel, N.	2020	Omission related brain responses reflect specific and unspecific action-effect couplings. NeuroImage, 215, 116840. https://doi.org/10.1016/j.neuroimage.2020.116840								
13	Kanoga, S., Hoshino, T., & Asoh, H.	2020	Independent low-rank matrix analysis-based automatic artifact reduction technique applied to three BCI paradigms. Frontiers in Human Neuroscience, 14, 173. https://doi.org/10.3389/fnhum.2020.00173								
14	Gennaro, F., & de Bruin, E. D.	2020	A pilot study assessing reliability and age-related differences in corticomuscular and intramuscular coherence in ankle dorsiflexors during walking.								

			Physiological reports, 8(4), e14378. https://doi.org/10.14814/phy2.14378								
15	Frömer, R., Lin, H., Dean Wolf, C. K., Inzlicht, M., & Shenhav, A.	2020	When effort matters: Expectations of reward and efficacy guide cognitive control allocation. BioRxiv, 2020-05. https://doi.org/10.1101/2020.05.14.095935	0.95	0.85						
16	Singh, F., Shu, I. W., Hsu, S. H., Link, P., Pineda, J. A., & Granholm, E.	2020	Modulation of frontal gamma oscillations improves working memory in schizophrenia. NeuroImage: Clinical, 27, 102339. https://doi.org/10.1016/j.nicl.2020.102339	0.5	0.5	0.5	0.5	0.5			
17	Koshiyama, D., Miyakoshi, M., Tanaka-Koshiyama, K., Joshi, Y. B., Molina, J. L., Sprock, J., ... & Light, G. A.	2020	Neurophysiologic characterization of resting state connectivity abnormalities in schizophrenia patients. Frontiers in Psychiatry, 11, 608154. https://doi.org/10.3389/fpsyt.2020.608154							0.7	
18	Stehwien, S., Henke, L., Hale, J., Brennan, J., & Meyer, L.	2020	The Little Prince in 26 languages: Towards a multilingual neuro-cognitive corpus. In Proceedings of the Second Workshop on Linguistic and Neurocognitive Resources (pp. 43-49). https://aclanthology.org/2020.lincr-1.6								
19	Wang, W. E., Ho, R. L., Gatto, B., Van Der Veen, S. M., Underation, M. K., Thomas, J. S., ... & Coombes, S. A.	2020	A novel method to understand neural oscillations during full-body reaching: a combined EEG and 3D virtual reality study. IEEE transactions on neural systems and rehabilitation engineering, 28(12), 3074-3082. https://doi.org/10.1109/TNSRE.2020.3039829								
20	El Kerdawy, M., El Halaby, M., Hassan, A., Maher, M., Fayed, H., Shawky, D., & Badawi, A.	2020	The automatic detection of cognition using eeg and facial expressions. Sensors, 20(12), 3516. https://doi.org/10.3390/s20123516	0.8	0.8		0.8	0.8	0.8		
21	Thammasan, N., & Miyakoshi, M.	2020	Cross-Frequency Power-Power Coupling Analysis: A useful cross-frequency measure to classify ICA-decomposed EEG. Sensors, 20(24), 7040. https://doi.org/10.3390/s20247040								
22	Anders, M., Anders, B., Kreuzer, M., Zinn, S., & Walter, C.	2020	Application of referencing techniques in EEG-based recordings of contact heat evoked potentials (CHEPS). Frontiers in human neuroscience, 14, 559969. https://doi.org/10.3389/fnhum.2020.559969								
23	Anders, P., Müller, H., Skjæret-Maroni,	2020	The influence of motor tasks and cut-off parameter selection on artifact subspace reconstruction in EEG								

	N., Vereijken, B., & Baumeister, J.		recordings. Medical & Biological Engineering & Computing, 58, 2673-2683. https://doi.org/10.1007/s11517-020-02252-3									
24	Menicucci, D., Di Gruttola, F., Cesari, V., Gemignani, A., Manzoni, D., & Sebastiani, L.	2020	Task-independent electrophysiological correlates of motor imagery ability from kinaesthetic and visual perspectives. Neuroscience, 443, 176-187. https://doi.org/10.1016/j.neuroscience.2020.07.038									
25	Clark, G. M., McNeel, C., Bigelow, F. J., & Enticott, P. G.	2020	The effect of empathy and context on face-processing ERPs. Neuropsychologia, 147, 107612. https://doi.org/10.1016/j.neuropsychologia.2020.107612									
26	Gabrielli, G., Bilucaglia, M., Zito, M., Laureanti, R., Caponetto, A., Circi, R., ... & Russo, V.	2020	Neurocoaching: exploring the relationship between coach and coachee by means of bioelectrical signal similarities. In 2020 42nd Annual International Conference of the IEEE Engineering in Medicine & Biology Society (EMBC) (pp. 3184-3187). IEEE. https://doi.org/10.1109/EMBC44109.2020.9176497									
27	Zhang, H., Zhao, M., Wei, C., Mantini, D., Li, Z., & Liu, Q.	2020	EEGdenoiseNet: A benchmark dataset for end-to-end deep learning solutions of EEG denoising. arXiv preprint arXiv:2009.11662. https://doi.org/10.48550/arXiv.2009.11662									
28	Braquet, A., Bayot, M., Tard, C., Defebvre, L., Derambure, P., Dujardin, K., & Delval, A.	2020	A new paradigm to study the influence of attentional load on cortical activity for motor preparation of step initiation. Experimental Brain Research, 238(3), 643-656. https://doi.org/10.1007/s00221-020-05739-5									
29	Gennaro, F., Maino, P., Kaelin-Lang, A., De Bock, K., & de Bruin, E. D.	2020	Corticospinal control of human locomotion as a new determinant of age-related sarcopenia: an exploratory study. Journal of clinical medicine, 9(3), 720. https://doi.org/10.3390/jcm9030720									
30	Arslan, S., Palasis, K., & Meunier, F.	2020	Electrophysiological differences in older and younger adults' anaphoric but not cataphoric pronoun processing in the absence of age-related behavioural slowdown. Scientific Reports, 10(1), 1-13. | https://doi.org/10.1038/s41598-020-75550-3									
31	Miyakoshi, M., Jurgiel, J., Dillon, A., Chang, S., Piacentini, J., Makeig, S., & Loo, S. K.	2020	Modulation of frontal oscillatory power during blink suppression in children: Effects of premonitory urge and reward. Cerebral cortex communications, 1(1), tgaa046. https://doi.org/10.1093/texcom/tgaa046									

32	Echtioui, A., Zouch, W., Ghorbel, M., Slima, M. B., Hamida, A. B., & Mhiri, C.	2020	Automated EEG artifact detection using independent component analysis. In 2020 5th International Conference on Advanced Technologies for Signal and Image Processing (ATSIP) (pp. 1-5). IEEE. https://doi.org/10.1109/ATSIP49331.2020.9231574								
33	Civai, C., Teodorini, R., & Carrus, E.	2020	Does unfairness sound wrong? A cross-domain investigation of expectations in music and social decision-making. Royal Society Open Science, 7(9), 190048. https://doi.org/10.1098/rsos.190048								
34	Hasan, S. S., Siddiquee, M. R., Marquez, J. S., & Bai, O.	2020	Enhancement of movement intention detection using EEG signals responsive to emotional music stimulus. IEEE Transactions on Affective Computing, 13(3), 1637-1650. https://doi.org/10.1109/TAFFC.2020.3025004	1.0?	1.0?	1.0?	1.0?	1.0?	1.0?		
35	Alipour, A., Mozhdehfarahbakhsh, A., Nouri, S., Petramfar, P., Tahamtan, M., Kamali, A. M., ... & Nami, M.	2020	Studies on the bottom-up and top-down neural information flow alterations in neurodegeneration. Journal of Alzheimer's Disease, 78(1), 169-183. https://doi.org/10.3233/JAD-200590								
36	Wunderlich, A., & Gramann, K.	2020	Eye-movement related brain potentials during assisted navigation in real-world. bioRxiv, 2020-06. https://doi.org/10.1101/2020.06.08.139469								0.3
37	Placidi, G., Cinque, L., & Polsinelli, M.	2020	Convolutional neural networks for automatic detection of artifacts from independent components represented in scalp topographies of EEG signals. arXiv preprint arXiv:2009.03696. https://doi.org/10.48550/arXiv.2009.03696								
38	Wu, X., Zhang, W., Fu, Z., Cheung, R. T., & Chan, R. H.	2020	An investigation of in-ear sensing for motor task classification. Journal of Neural Engineering, 17(6), 066010. https://doi.org/10.1088/1741-2552/abc1b6								
39	Kaur, R., Korolkov, M., Hernandez, M. E., & Sowers, R.	2020	Automatic identification of brain independent components in electroencephalography data collected while standing in a virtually immersive environment-A Deep Learning-Based approach. In 2020 42nd Annual International Conference of the IEEE Engineering in Medicine & Biology Society (EMBC) (pp. 95-98). IEEE. https://doi.org/10.1109/EMBC44109.2020.9175741								
40	Daeglau, M., Zich, C., Welzel, J., Saak, S. K., Scheffels, J. F., & Kranczioch, C.	2020	Motor Imagery EEG neurofeedback skill acquisition in the context of declarative interference and sleep. bioRxiv, 2020-12. https://doi.org/10.1101/2020.12.11.420919								

41	Timpe, C. M. F.	2020	Cortical Excitability in Schizophrenia and Bipolar Disorder: A Study of an EEG-based Index (Master's thesis).								0.3
42	Kovalev, D. Y. E., Shanin, I. A., & Tirikov, E. M.	2020	Multidisciplinary neuroinformatics problems for execution in distributed computing infrastructures. Системы и средства информатики, 30(2), 43-55. https://doi.org/10.14357/08696527200205								
43	Handiru, V. S., Alivar, A., Hoxha, A., Saleh, S., Selvan, E., Yue, G., & Allexandre, D.	2020	Graph-theoretical Analysis of EEG Functional Connectivity during Balance Perturbation in Traumatic Brain Injury. bioRxiv, 2020-10. https://doi.org/10.1101/2020.10.08.332353								
44	Hsu, S. H., Lin, Y., Onton, J., Jung, T. P., & Makeig, S.	2020	Unsupervised Learning of Brain State Dynamics during Emotion Imagery using High-Density EEG. bioRxiv, 2020-10. https://doi.org/10.1101/2020.10.29.361394								
45	Guo, L. L.	2020	The Temporal Structure of Neural Processes Underlying Human Precision Grasp Computations. University of Toronto (Canada).								
46	Krol, L. R.	2020	Neuroadaptive technology: concepts, tools, and validations (Doctoral dissertation, Dissertation, Berlin, Technische Universität Berlin, 2020).								
47	Galang, C. M.	2020	Pain Observation, Empathy, and the Sensorimotor System: Behavioural and Neurophysiological Explorations (Doctoral dissertation). http://hdl.handle.net/11375/25917								
48	Blundon, E. G.	2020	Measurement of awareness at the end of life (Doctoral dissertation, University of British Columbia). https://doi.org/10.14288/1.0390919								
49	Kaufman, N.	2020	To see or not to see the error of one´ s ways: Consciousness and the Error-Related Negativity.	0.7	0.7	0.7	0.7	0.7	0.7		
50	Magnusson, O.	2020	Attentional selection and suppression in non-clinical adults: An event-related potential study.								
51	Jin, H.	2020	The Behavioral and Neural Indicators of Face Specific Processing: Holistic Processing and the N170 (Doctoral dissertation, ResearchSpace@ Auckland). http://hdl.handle.net/2292/50468								
52	Zapała, D., Iwanowicz, P., Francuz, P., & Augustynowicz, P.	2020	Handedness Effects on Movement Imagery During Kinesthetic and Visual-Motor Conditions. An EEG Study. https://doi.org/10.21203/rs.3.rs-107906/v1								
53	Cvetkovic, I.	2020	Machine Learning-Based Prediction of Facial Emotional Expressions from EEG.	0.6	0.6						

54	DiGirolamo, M. A.	2020	The Complex Model of Empathy: Investigating the Effect of Arousal on Bottom-Up and Top-Down Empathy Components (Doctoral dissertation, Brandeis University).								
55	Peterson, E. K.	2020	Associations between EEG and cognition in Parkinson's disease. http://dx.doi.org/10.26021/11217								
56	Barnstaple, R. E.	2020	An applied neurobiological model of dance, why it matters, and how it heals. http://hdl.handle.net/10315/38198								
57	Do, N. T. T.	2020	Human brain dynamics during multitasking physical navigation (Doctoral dissertation). http://hdl.handle.net/10453/148843								
58	Xie, T.	2020	Cognitive reserve, individual alpha frequency in Parkinson’s disease. http://dx.doi.org/10.26021/10739								
59	Apelgren, F., & Pettersson, I.	2020	Assessing Cognitive Workload Between Different Tasks- Using EEG to develop and examine a method to measure variation of cognitive workload between different levels of difficulty.	0.85	0.85						
60	Miyakoshia, M., Jurgielb, J., Dillonb, A., Changb, S., Piacentinib, J., Makeiga, S., ... & Miyakoshi, M.	2020	Running title: EEG study on blink suppression in healthy children.								

2021

Index	Author	Year	Journal information	muscle	eye	heart	line noise	channel noise	other	brain	brain removed
1	Aamodt, A., Nilsen, A. S., Thürer, B., Moghadam, F. H., Kauppi, N., Juel, B. E., & Storm, J. F.	2021	EEG signal diversity varies with sleep stage and aspects of dream experience. Frontiers in psychology, 12, 655884.								
2	Abou-Abbas, L., van Noordt, S., Desjardins, J. A., Cichonski, M., & Elsabbagh, M.	2021	Use of empirical mode decomposition in ERP analysis to classify familial risk and diagnostic outcomes for autism spectrum disorder. Brain Sciences, 11(4), 409.								
3	Ahlström, C., Zemblys, R., Jansson, H., Forsberg, C.,	2021	Effects of partially automated driving on the development of driver sleepiness. Accident Analysis & Prevention, 153, 106058.								

	Karlsson, J., & Anund, A.										
4	Akella, A., Singh, A. K., Leong, D., Lal, S., Newton, P., Clifton-Bligh, R., ... & Lin, C. T.	2021	Classifying multi-level stress responses from brain cortical EEG in nurses and non-health professionals using machine learning auto encoder. IEEE Journal of Translational Engineering in Health and Medicine, 9, 1-9.	0.9	0.9						
5	Allexandre, D., Handiru, V. S., Hoxha, A., Mark, D., Suviseshamuthu, E. S., & Yue, G. H.	2021	Altered Modulation of the Movement-Related Beta Desynchronization with Force in Stroke–a Pilot Study. In 2021 43rd Annual International Conference of the IEEE Engineering in Medicine & Biology Society (EMBC) (pp. 6751-6754). IEEE.								
6	Arnau, S., Brümmer, T., Liegel, N., & Wascher, E.	2021	Inverse effects of time-on-task in task-related and task-unrelated theta activity. Psychophysiology, 58(6), e13805.								0.5
7	Bakhtyari, M., & Mirzaei, S.	2021	Detection of ADHD Disorder Using Dynamic Connectivity Tensors in Bidirectional Circular Reservoir Computing. In 2021 28th National and 6th International Iranian Conference on Biomedical Engineering (ICBME) (pp. 265-270). IEEE. https://doi.org/10.1109/ICBME54433.2021.9750383								
8	Banks, P.	2021	Where not what: the role of spatial-motor processing in decision-making (Doctoral dissertation, McMaster University).								
9	Barban, F., Chiappalone, M., Bonassi, G., Mantini, D., & Semprini, M.	2021	Yet another artefact rejection study: an exploration of cleaning methods for biological and neuromodulatory noise. Journal of Neural Engineering, 18(4), 0460c2.								
10	Bates, K. E.	2021	Picture this: an investigation of the neural and behavioural correlates of mental imagery in childhood and adulthood with implications for children with ADHD (Doctoral dissertation, UCL (University College London)).								
11	Baykova, R.	2021	Investigating the role of Bayesian inference in duration perception (Doctoral dissertation, University of Sussex).	0.9	0.9						
12	Bennett, J. D., John, S. E., Grayden, D. B., & Burkitt, A. N.	2021	A neurophysiological approach to spatial filter selection for adaptive brain–computer interfaces. Journal of Neural Engineering, 18(2), 026017. https://doi.org/10.1088/1741-2552/abd51f								
13	Boord, M. S., Davis, D. H., Psaltis, P. J., Coussens, S. W., Feuerriegel, D., Garrido, M. I., ... & Keage, H. A.	2021	DelIrium VULnerability in GEriatrics (DIVULGE) study: a protocol for a prospective observational study of electroencephalogram associations with incident postoperative delirium. BMJ Neurology Open, 3(2).								
14	Bower, I. S., Clark, G. M., Tucker, R., Hill, A., Lum, J., Mortimer, M., & Enticott, P. G.	2021	Enlarged interior built environment scale modulates high frequency EEG oscillations.								0.3

15	Bu-Omer, H. M., Gofuku, A., Sato, K., & Miyakoshi, M.	2021	Parieto-occipital alpha and Low-Beta EEG power reflect sense of agency. Brain Sciences, 11(6), 743.								
16	Büchel, D., Lehmann, T., Sandbakk, Ø., & Baumeister, J.	2021	EEG-derived brain graphs are reliable measures for exploring exercise-induced changes in brain networks. Scientific reports, 11(1), 20803.								
17	Chandrakumar, D., Coussens, S., Keage, H. A. D., Banks, S., Dorrian, J., & Loetscher, T.	2021	Monotonous driving induces shifts in spatial attention as a function of handedness. Scientific Reports, 11(1), 1-12.								
18	Chen, I. C., Chang, C. H., Chang, Y., Lin, D. S., Lin, C. H., & Ko, L. W.	2021	Neural dynamics for facilitating adhd diagnosis in preschoolers: central and parietal delta synchronization in the kiddie continuous performance test. IEEE Transactions on Neural Systems and Rehabilitation Engineering, 29, 1524-1533.								
19	Chen, X., Li, C., Liu, A., McKeown, M. J., Qian, R., & Wang, Z. J.	2021	Toward Open-World Electroencephalogram Decoding Via Deep Learning: A Comprehensive Survey. arXiv preprint arXiv:2112.06654.								
20	Cline, C. C., Lucas, M. V., Sun, Y., Menezes, M., & Etkin, A.	2021	Advanced artifact removal for automated TMS-EEG data processing. In 2021 10th International IEEE/EMBS Conference on Neural Engineering (NER) (pp. 1039-1042). IEEE. https://doi.org/10.1109/NER49283.2021.9441147								
21	Contier, F., Weymar, M., Wartenburger, I., & Rabovsky, M.	2021	The P600, but not the N400, is modulated by sustained attention. bioRxiv.		0.3						
22	Contier, F., Weymar, M., Wartenburger, I., & Rabovsky, M.	2021	Sustained attention as measured by RT variability is a strong modulator for the P600, but not the N400. bioRxiv, 2021-11.		0.3						
23	Corcoran, A. W., Alday, P. M., Coussens, S., Bellan, V., Howlett, C. A., Immink, M. A., ... & Bornkessel-Schlesewsky, I.	2021	EEG and behavioral correlates of attentional processing while walking and navigating naturalistic environments.		0.9	0.9					
24	Cromarty, L., Shirwaiker, R., & Wang, P.	2021	Brain Network Topology and the Cognitive Redline: First Results from a Pilot Study.								
25	Cruz-Garza, J. G., Darfler, M., Rounds, J. D., Gao, E., & Kalantari, S.	2021	EEG-based investigation of the impact of classroom design on cognitive performance of students. arXiv preprint arXiv:2102.03629.	0.9	0.9	0.9	0.9	0.9			

26	Czeszumski, A., Gert, A. L., Keshava, A., Ghadirzadeh, A., Kalthoff, T., Ehinger, B. V., ... & König, P.	2021	Coordinating with a robot partner affects neural processing related to action monitoring. Frontiers in Neurorobotics, 15, 686010.								
27	Daeglau, M., Zich, C., Welzel, J., Saak, S. K., Scheffels, J. F., & Kranczioch, C.	2021	Event-related desynchronization in motor imagery with EEG neurofeedback in the context of declarative interference and sleep. Neuroimage: Reports, 1(4), 100058.								
28	Daly, I.	2021	Removal of physiological artifacts from simultaneous EEG and fMRI recordings. Clinical Neurophysiology, 132(10), 2371-2383. https://doi.org/10.1016/j.clinph.2021.05.036								
29	Damián-Chávez, M. M., Ledesma-Coronado, P. E., Drexel-Romo, M., Ibarra-Zárate, D. I., & Alonso-Valerdi, L. M.	2021	Environmental noise at library learning commons affects student performance and electrophysiological functioning. Physiology & Behavior, 241, 113563. https://doi.org/10.1016/j.physbeh.2021.113563								
30	Delaux, A., de Saint Aubert, J. B., Ramanoël, S., Bécu, M., Gehrke, L., Klug, M., ... & Arleo, A.	2021	Mobile brain/body imaging of landmark-based navigation with high-density EEG. European Journal of Neuroscience, 54(12), 8256-8282.								
31	Delorme, A., Truong, D., Martinez-Cancino, R., Pernet, C., Sivagnanam, S., Yoshimoto, K., ... & Makeig, S.	2021	Tools for importing and evaluating BIDS-EEG formatted data. In 2021 10th International IEEE/EMBS Conference on Neural Engineering (NER) (pp. 210-213). IEEE.								
32	Desjardins, J. A., van Noordt, S., Huberty, S., Segalowitz, S. J., & Elsabbagh, M.	2021	EEG Integrated Platform Lossless (EEG-IP-L) pre-processing pipeline for objective signal quality assessment incorporating data annotation and blind source separation. Journal of Neuroscience Methods, 347, 108961.								
33	Do, T. T. N., Jung, T. P., & Lin, C. T.	2021	Retrosplenial Segregation Reflects the Navigation Load During Ambulatory Movement. IEEE Transactions on Neural Systems and Rehabilitation Engineering, 29, 488-496.								
34	Duan, W., Chen, X., Wang, Y. J., Zhao, W., Yuan, H., & Lei, X.	2021	Reproducibility of power spectrum, functional connectivity and network construction in resting-state EEG. Journal of Neuroscience Methods, 348, 108985.								

35	Eisma, J., Rawls, E., Long, S., Mach, R., & Lamm, C.	2021	Frontal midline theta differentiates separate cognitive control strategies while still generalizing the need for cognitive control. Scientific Reports, 11(1), 1-14.								
36	Elsayed, N. E., Tolba, A. S., Rashad, M. Z., Belal, T., & Sarhan, S.	2021	A deep learning approach for brain computer interaction-motor execution EEG signal classification. IEEE Access, 9, 101513-101529.								
37	Fairchild, G. T., Marini, F., & Snow, J. C.	2021	Graspability Modulates the Stronger Neural Signature of Motor Preparation for Real Objects vs. Pictures. Journal of Cognitive Neuroscience, 33(12), 2477-2493.								
38	Fera, C. J	2021	Artifact Removal from Electroencephalogram Signals Using Dictionary Learning (Doctoral dissertation, San Diego State University).								
39	Forner, N. A.	2021	Altered Alpha Oscillatory Power Dynamics Underlie Difficulties with Cognitive Flexibility (Doctoral dissertation, University of New Hampshire).								
40	Frömer, R., Lin, H., Dean Wolf, C. K., Inzlicht, M., & Shenhav, A	2021	Expectations of reward and efficacy guide cognitive control allocation. Nature Communications, 12(1), 1030.	0.95	0.85					0.01	
41	Galang, C. M., Jenkins, M., Fahim, G., & Obhi, S. S.	2021	Exploring the relationship between social power and the ERP components of empathy for pain. Social Neuroscience, 16(2), 174-188.								
42	Galdino, L., Fernandes, T., Schmidt, K. E., & dos Santos, N. A.	2021	Abnormal Low-Gamma Small-World Response After Visual Stimulation in Schizophrenia.								
43	Garrett-Ruffin, S., Hindash, A. C., Kaczkurkin, A. N., Mears, R. P., Morales, S., Paul, K., ... & Keil, A.	2021	Open science in psychophysiology: An overview of challenges and emerging solutions. International Journal of Psychophysiology, 162, 69-78. https://doi.org/10.1016/j.ijpsycho.2021.02.005								
44	Gehrke, L., & Gramann, K.	2021	Single-trial regression of spatial exploration behavior indicates posterior EEG alpha modulation to reflect egocentric coding. European Journal of Neuroscience, 54(12), 8318-8335.		0.5						0.5
45	Gholamipour, N., & Ghassemi, F.	2021	Estimation of the independent components reliability of EEG signal in a clinical application. Biomedical Signal Processing and Control, 65, 102336. https://doi.org/10.1016/j.bspc.2020.102336								
46	Gramann, K., Hohlefeld, F. U., Gehrke, L., & Klug, M.	2021	Human cortical dynamics during full-body heading changes. Scientific Reports, 11(1), 18186.								
47	Greco, A., Gallitto, G., D’Alessandro, M., & Rastelli, C.	2021	Increased entropic brain dynamics during DeepDream-induced altered perceptual phenomenology. Entropy, 23(7), 839.								

48	Guan, Y., & Wessel, J. R.	2021	Timing-dependent differential effects of unexpected events on error processing reveal the interactive dynamics of surprise and error processing. Psychophysiology, 58(12), e13922.								
49	Guarnieri, R.,	2021	Real-time analysis of high-density EEG signals for closed-loop applications.(Doctoral dissertation, University of Leuven).								
50	Guarnieri, R., Zhao, M., Taberna, G. A., Ganzetti, M., Swinnen, S. P., & Mantini, D.	2021	RT-NET: real-time reconstruction of neural activity using high-density electroencephalography. Neuroinformatics, 19, 251-266.								
51	Guo, L. L., Oghli, Y. S., Frost, A., & Niemeier, M.	2021	Multivariate Analysis of Electrophysiological Signals Reveals the Time Course of Precision Grasps Programs: Evidence for Nonhierarchical Evolution of Grasp Control. Journal of Neuroscience, 41(44), 9210-9222.								
52	Hasan, S. S., & Bai, O	2021	VMD-WSST: A Combined BCI Algorithm to Predict Self-paced Gait Intention. In 2021 IEEE International Conference on Systems, Man, and Cybernetics (SMC) (pp. 3188-3193). IEEE. https://doi.org/10.1109/SMC52423.2021.9658856								
53	Hasan, S. S., Marquez, J. S., Siddiquee, M. R., Fei, D. Y., & Bai, O.	2021	Preliminary Study on Real-Time Prediction of Gait Acceleration Intention from Volition-Associated EEG Patterns. IEEE Access, 9, 62676-62686.								
54	Hatlestad-Hall, C., Bruña, R., Syvertsen, M. R., Erichsen, A., Andersson, V., Vecchio, F., ... & Haraldsen, I. H.	2021	Source-level EEG and graph theory reveal widespread functional network alterations in focal epilepsy. Clinical Neurophysiology, 132(7), 1663-1676.								
55	Hatlestad-Hall, C., Bruña, R., Erichsen, A., Andersson, V., Syvertsen, M. R., Skogan, A. H., ... & Haraldsen, I. H.	2021	The organization of functional neurocognitive networks in focal epilepsy correlates with domain-specific cognitive performance. Journal of Neuroscience Research, 99(10), 2669-2687.								
56	Haugwitz, L.	2021	Stress effects on endogenous and exogenous visuospatial attention (Master's thesis, University of Twente).								
57	Hausfeld, L., Shiell, M., Formisano, E., & Riecke, L.	2021	Cortical processing of distracting speech in noisy auditory scenes depends on perceptual demand. Neuroimage, 228, 117670.								
58	Hervault, M., Zanone, P. G., Buisson, J. C., & Huys, R.	2021	Cortical sensorimotor activity in the execution and suppression of discrete and rhythmic movements. Scientific Reports, 11(1), 22364.								

59	Hodapp, A., & Grimm, S.	2021	Neural signatures of temporal regularity and recurring patterns in random tonal sound sequences. European Journal of Neuroscience, 53(8), 2740-2754.								
60	Hollenstein, N., Tröndle, M., Plomecka, M., Kiegeland, S., Özyurt, Y., Jäger, L. A., & Langer, N.	2021	Reading task classification using EEG and eye-tracking data. arXiv preprint arXiv:2112.06310.	0.8	0.8	0.8	0.8	0.8			
61	Houtman, S. J., Lammertse, H. C., van Berkel, A. A., Balagura, G., Gardella, E., Ramautar, J. R., ... & Linkenkaer-Hansen, K.	2021	STXBP1 syndrome is characterized by inhibition-dominated dynamics of resting-state EEG. Frontiers in Physiology, 12, 2293.								
62	Hsu, H. C., Peng, P. H., Peng, P. C., Hsu, T. Y., & Chuang, C. H.	2021	Cooperative and Competitive-related Inter-Brain Synchrony during Gaming. In 2021 IEEE International Conference on Systems, Man, and Cybernetics (SMC) (pp. 3391-3395). IEEE. https://doi.org/10.1109/SMC52423.2021.9658676	0.9	0.9	0.9	0.9				
63	Hucke, C. I., Heinen, R. M., Pacharra, M., Wascher, E., & Van Thriel, C.	2021	Spatiotemporal processing of bimodal odor lateralization in the brain using electroencephalography microstates and source localization. Frontiers in Neuroscience, 14, 620723.								
64	Hui, K. Y., Wong, C. H., Siu, A. M., Lee, T. M., & Chan, C. C.	2021	Cognitive and Emotional Appraisal of Motivational Interviewing Statements: An Event-Related Potential Study. Frontiers in Human Neuroscience, 15, 727175.								
65	Hülsemann, M. J., & Rasch, B.	2021	Embodiment of sleep-related words: Evidence from event-related potentials. Psychophysiology, 58(8), e13824.	0.25	0.9	0.6					
66	Ibitoye, R. T., Castro, P., Desowska, A., Cooke, J., Edwards, A. E., Guven, O., ... & Bronstein, A. M.	2021	Small vessel disease disrupts EEG postural brain networks in 'unexplained dizziness in the elderly'. Clinical Neurophysiology, 132(11), 2751-2762.								
67	Ismail, L.	2021	Topological Changes in the Functional Brain Networks Induced by Isometric Force Exertions Using a Graph Theoretical Approach: An EEG-Based Neuroergonomics Study.								
68	Jin, Z.	2021	State-of-the-art EEG artifact removal evaluation. bioRxiv, 2021-10.								
69	Kastrati, A., Płomecka, M. B., Pascual, D., Wolf, L., Gillioz, V.,	2021	EEGEyeNet: a simultaneous electroencephalography and eye-tracking dataset and benchmark for eye movement prediction. arXiv preprint arXiv:2111.05100.								

	Wattenhofer, R., & Langer, N.										
70	Kim, H., Kim, Y., Miyakoshi, M., Stapornchaisit, S., Yoshimura, N., & Koike, Y.	2021	Brain Activity Reflects Subjective Response to Delayed Input When Using an Electromyography-Controlled Robot. Frontiers in Systems Neuroscience, 128.								
71	Klug, M., & Gramann,	2021	Identifying key factors for improving ICA-based decomposition of EEG data in mobile and stationary experiments. European Journal of Neuroscience, 54(12), 8406-8420.								
72	Ko, L. W., Stevenson, C., Chang, W. C., Yu, K. H., Chi, K. C., Chen, Y. J., & Chen, C. H.	2021	Integrated gait triggered mixed reality and neurophysiological monitoring as a framework for next-generation ambulatory stroke rehabilitation. IEEE Transactions on Neural Systems and Rehabilitation Engineering, 29, 2435-2444.							0.7	
73	Koshiyama, D., Miyakoshi, M., Joshi, Y. B., Molina, J. L., Tanaka-Koshiyama, K., Sprock, J., ... & Light, G. A.	2021	Neural network dynamics underlying gamma synchronization deficits in schizophrenia. Progress in Neuro-Psychopharmacology and Biological Psychiatry, 107, 110224.								
74	Koshiyama, D., Miyakoshi, M., Joshi, Y. B., Nakanishi, M., Tanaka-Koshiyama, K., Sprock, J., & Light, G. A.	2021	Sources of the frontocentral mismatch negativity and P3a responses in schizophrenia patients and healthy comparison subjects. International Journal of Psychophysiology, 161, 76-85.							0.8	
75	Koshiyama, D., Miyakoshi, M., Joshi, Y. B., Nakanishi, M., Tanaka-Koshiyama, K., Sprock, J., & Light, G. A.	2021	Source decomposition of the frontocentral auditory steady-state gamma band response in schizophrenia patients and healthy subjects. Psychiatry and Clinical Neurosciences, 75(5), 172-179.							0.7	
76	Koshiyama, D., Miyakoshi, M., Tanaka-Koshiyama, K., Joshi, Y. B., Sprock, J., Braff, D. L., & Light, G. A.	2021	Abnormal phase discontinuity of alpha-and theta-frequency oscillations in schizophrenia. Schizophrenia research, 231, 73-81.							0.7	
77	Koshiyama, D., Miyakoshi, M., Thomas, M. L., Joshi, Y. B., Molina, J. L., Tanaka-	2021	Unique contributions of sensory discrimination and gamma synchronization deficits to cognitive, clinical, and psychosocial functional impairments in schizophrenia. Schizophrenia Research, 228, 280-287.	0.7	0.7	0.7	0.7	0.7			

	Koshiyama, K., ... & Light, G. A.										
78	Kulkarni, S., & Patil, P. R.	2021	Analysis of DEAP dataset for emotion recognition. In International Conference on Intelligent and Smart Computing in Data Analytics: ISCDA 2020 (pp. 67-76). Springer Singapore. https://doi.org/10.1007/978-981-33-6176-8_8								
79	Kumaravel, V. P., Kartsch, V., Benatti, S., Vallortigara, G., Farella, E., & Buiatti, M.	2021	Efficient artifact removal from low-density wearable EEG using artifacts subspace reconstruction. In 2021 43rd Annual International Conference of the IEEE Engineering in Medicine & Biology Society (EMBC) (pp. 333-336). IEEE.								
80	Ladouce, S., Mustile, M., & Dehais, F.	2021	Capturing cognitive events embedded in the real-world using mobile EEG and Eye-Tracking. bioRxiv, 2021-11.								
81	Lanzone, J., Ricci, L., Tombini, M., Boscarino, M., Mecarelli, O., Pulitano, P., ... & Assenza, G.	2021	The effect of Perampanel on EEG spectral power and connectivity in patients with focal epilepsy. Clinical Neurophysiology, 132(9), 2176-2183. https://doi.org/10.1016/j.clinph.2021.05.026								
82	Lauer, T., Schmidt, F., & Võ, M. L. H.	2021	The role of contextual materials in object recognition. Scientific reports, 11(1), 1-12.		0.5						
83	Laureanti, R., Bilucaglia, M., Zito, M., Circi, R., Fici, A., Rivetti, F., ... & Russo, V.	2021	Yellow (lens) better: Bioelectrical and biometrical measures to assess arousing and focusing effects. In 2021 43rd Annual International Conference of the IEEE Engineering in Medicine & Biology Society (EMBC) (pp. 6163-6166). IEEE. https://doi.org/10.1109/EMBC46164.2021.9630201								0.7
84	Lehmann, T., Büchel, D., Mouton, C., Gokeler, A., Seil, R., & Baumeister, J.	2021	Functional cortical connectivity related to postural control in patients six weeks after anterior cruciate ligament reconstruction. Frontiers in Human Neuroscience, 15, 655116.								
85	Lengali, L., Hippe, J., Hatlestad-Hall, C., Rygvold, T. W., Sneve, M. H., & Andersson, S.	2021	Sensory-induced human LTP-Like synaptic plasticity–using visual evoked potentials to explore the relation between LTP-like synaptic plasticity and visual perceptual learning. Frontiers in Human Neuroscience, 15, 684573.								
86	Li, L. Y., Schiffman, J., Hu, D. K., Lopour, B. A., & Martin, E. A.	2021	An Effortful Approach to Social Affiliation in Schizophrenia: Preliminary Evidence of Increased Theta and Alpha Connectivity during a Live Social Interaction. Brain Sciences, 11(10), 1346.								
87	Liang, M., Zheng, J., Isham, E., & Ekstrom, A.	2021	Common and distinct roles of frontal midline theta and occipital alpha oscillations in coding temporal intervals and spatial distances. Journal of Cognitive Neuroscience, 33(11), 2311-2327.	0.9	0.9	0.9	0.9	0.9			
88	Liebherr, M., Corcoran, A. W., Alday, P. M.,	2021	EEG and behavioral correlates of attentional processing while walking and navigating naturalistic environments. Scientific reports, 11(1), 22325.	0.9	0.9						

	Coussens, S., Bellan, V., Howlett, C. A., ... & Bornkessel-Schlesewsky, I.										
89	Liebherr, M., Corcoran, A. W., Alday, P. M., Coussens, S., Bellan, V., Howlett, C. A., ... & Bornkessel-Schlesewsky, I.	2021	Additive effects of cognitive load, motor demand, and environmental complexity on attention: A real-world eeg-study. bioRxiv, 2021-05.		0.9	0.9					
90	Lin, H. S.	2021	Hypothesis-Driven Source Separation and Dimension Reduction of Neural Time Series Data (Doctoral dissertation, University of Toronto (Canada)).								
91	Lin, Z.	2021	Electroencephalography of the Trail Making Test. (Master's thesis, University of Toronto).								
92	Lin, Z., Tam, F., Churchill, N. W., Lin, F. H., MacIntosh, B. J., Schweizer, T. A., & Graham, S. J.	2021	Trail making test performance using a touch-sensitive tablet: Behavioral kinematics and electroencephalography. Frontiers in Human Neuroscience, 15, 663463.								
93	Lopes, F., Leal, A., Medeiros, J., Pinto, M. F., Dourado, A., Dümpelmann, M., & Teixeira, C.	2021	Automatic electroencephalogram artifact removal using deep convolutional neural networks. IEEE Access, 9, 149955-149970. https://doi.org/10.1109/ACCESS.2021.3125728								
94	Magosso, E., Ricci, G., & Ursino, M.	2021	Alpha and theta mechanisms operating in internal-external attention competition. Journal of Integrative Neuroscience, 20(1), 1-19.								
95	Malcolm, B. R., Foxe, J. J., Joshi, S., Verghese, J., Mahoney, J. R., Molholm, S., & De Sanctis, P.	2021	Aging-related changes in cortical mechanisms supporting postural control during base of support and optic flow manipulations. European Journal of Neuroscience, 54(12), 8139-8157. https://doi.org/10.1111/ejn.15004								
96	Mayeli, A., Al Zoubi, O., Henry, K., Wong, C. K., White, E. J., Luo, Q., ... & Tulsa 1000 Investigators.	2021	Automated pipeline for EEG artifact reduction (APPEAR) recorded during fMRI. Journal of neural engineering, 18(4), 0460b4.								
97	Mazurek, K. A., Patelaki, E., Foxe, J. J., & Freedman, E. G.	2021	Using the MoBI motion capture system to rapidly and accurately localize EEG electrodes in anatomic space. European Journal of Neuroscience, 54(12), 8396-8405.								

98	McDermott, E. J., Raggam, P., Kirsch, S., Belardinelli, P., Ziemann, U., & Zrenner, C.	2021	Artifacts in EEG-based BCI therapies: friend or foe?. Sensors, 22(1), 96.								
99	Meghdadi, A. H., Stevanović Karić, M., McConnell, M., Rupp, G., Richard, C., Hamilton, J., ... & Berka, C.	2021	Resting state EEG biomarkers of cognitive decline associated with Alzheimer's disease and mild cognitive impairment. PloS one, 16(2), e0244180.								
100	Menicucci, D., Lunghi, C., Zaccaro, A., Morrone, M. C., & Gemignani, A.	2021	Sleep Slow Oscillations and Spindles encode ocular dominance plasticity and promote its consolidation in adult humans. bioRxiv.								
101	Miyakoshi, M., Gehrke, L., Gramann, K., Makeig, S., & Iversen, J.	2021	The AudioMaze: An EEG and motion capture study of human spatial navigation in sparse augmented reality. European Journal of Neuroscience, 54(12), 8283-8307. https://doi.org/10.1111/ejn.15131								
102	Miyakoshi, M., Nariai, H., Rajaraman, R. R., Bernardo, D., Shrey, D. W., Lopour, B. A., ... & Hussain, S. A.	2021	Automated preprocessing and phase-amplitude coupling analysis of scalp EEG discriminates infantile spasms from controls during wakefulness. Epilepsy Research, 178, 106809.								
103	Mohammed, B. O. H. M.	2021	Study on Brain Activities Associated with the Sense of Agency Using EEG Measurements. (Doctoral dissertation, Okayama university).								
104	Movahed, R. A., Hamedani, N. E., Sadredini, S. Z., & Rezaeian, M. R.	2021	An Automated EEG-based mild cognitive impairment diagnosis framework using spectral and functional connectivity features. In 2021 28th National and 6th International Iranian Conference on Biomedical Engineering (ICBME) (pp. 271-275). IEEE. https://doi.org/10.1109/ICBME54433.2021.975029110.1109/ICBME54433.2021.9750291								
105	Movahed, R. A., Jahromi, G. P., Shahyad, S., & Meftahi, G. H.	2021	A major depressive disorder classification framework based on EEG signals using statistical, spectral, wavelet, functional connectivity, and nonlinear analysis. Journal of Neuroscience Methods, 358, 109209. https://doi.org/10.1016/j.jneumeth.2021.109209								
106	Nilsson, M.	2021	No relationship between frontal alpha asymmetry and motivational and personality traits.(Master's thesis, University of Skövde, School of Bioscience.).	0.9	0.9						
107	Ogunniyi, V., Abugaber, D.,	2021	Neural Oscillations as Predictors of Variability in Second Language Proficiency. Columbia Undergraduate Science Journal, 15.								

	Finestrat, I., Luque, A., & Morgan-Short, K.										
108	Ogunniyi, V., Abugaber, D., Finestrat, I., Luque, A., & Morgan-Short, K.	2021	Predicting Second Language Proficiency with Resting-state Brain Rhythms.<br>Columbia Undergraduate Science Journal, 15, 39-54								
109	Pang, L., Guo, L., Zhang, J., Wanyan, X., Qu, H., & Wang, X.	2021	Subject-specific mental workload classification using EEG and stochastic configuration network (SCN). Biomedical Signal Processing and Control, 68, 102711. https://doi.org/10.1016/j.bspc.2021.102711								
110	Parhiz, S. T.	2021	Effect of Levodopa on EEG Connectivity in Parkinson's Patients (Doctoral dissertation, The University of Western Ontario).								
111	Pari, R. K.	2021	Neural dynamics of stroboscopic stimulation at different stimulation frequencies. bioRxiv, 2021-06.								
112	Patel, A. N., Chau, G., Chang, C., Sun, A., Huang, J., Jung, T. P., & Gilja, V.	2021	Affective response to volitional input perturbations in obstacle avoidance and target tracking games. In 2021 43rd Annual International Conference of the IEEE Engineering in Medicine & Biology Society (EMBC) (pp. 6679-6682). IEEE. https://doi.org/10.1109/EMBC46164.2021.9630523								
113	Pei, L., & Ouyang, G.	2021	Online recognition of handwritten characters from scalp-recorded brain activities during handwriting. Journal of Neural Engineering, 18(4), 046070.								
114	Persici, V., Blain, S., Iversen, J. R., Key, A. P., Kotz, A. S., McAuley, J. D., & Gordon, R. L.	2021	Individual differences in neural entrainment to rhythm predict spoken grammar skills in six-year-old children. PsyArXiv.								
115	Piazza, C., Pirovano, I., Mastropietro, A., Genova, C., Gagliardi, C., Turconi, A. C., ... & Biffi, E.	2021	Development and Preliminary Testing of a System for the Multimodal Analysis of Gait Training in a Virtual Reality Environment. Electronics, 10(22), 2838.								0.4
116	Placidi, G., Cinque, L., & Polsinelli, M.	2021	A fast and scalable framework for automated artifact recognition from EEG signals represented in scalp topographies of independent components. Computers in Biology and Medicine, 132, 104347. https://doi.org/10.1016/j.compbiomed.2021.104347								
117	Popov, T., Miller, G. A., Rockstroh, B., Jensen, O., & Langer, N.	2021	Alpha oscillations link action to cognition: An oculomotor account of the brain’s dominant rhythm. BioRxiv, 2021-09.	0.8	0.8	0.8	0.8	0.8			
118	Protzak, J., & Gramann, K.	2021	EEG beta-modulations reflect age-specific motor resource allocation during dual-task walking. Scientific reports, 11(1), 1-11.								

119	Protzak, J., Wiczorek, R., & Gramann, K.	2021	Peripheral visual perception during natural overground dual-task walking in older and younger adults. Neurobiology of aging, 98, 146-159. https://doi.org/10.1016/j.neurobiolaging.2020.10.009								
120	Rajabioun, R., Akyürek, A. Ö., & Sezer, E. A.	2021	Deep Learning Approach for EEG Artifact Identification and Classification. In 2021 6th International Conference on Computer Science and Engineering (UBMK) (pp. 320-325). IEEE. https://doi.org/10.1109/UBMK52708.2021.9558979								
121	Rawls, E., & Lamm, C.	2021	The aversion positivity: Mediofrontal cortical potentials reflect parametric aversive prediction errors and drive behavioral modification following negative reinforcement. Cortex, 140, 26-39.	0.7	0.7	0.7	0.7	0.7			
122	Rawls, E., Wolkowicz, N. R., Ham, L. S., & Lamm, C.	2021	Negative urgency as a risk factor for hazardous alcohol use: Dual influences of cognitive control and reinforcement processing. Neuropsychologia, 161, 108009.	0.7	0.7	0.7	0.7	0.7			
123	Reiser, J. E., Wascher, E., Rinkenauer, G., & Arnau, S.	2021	Cognitive-motor interference in the wild: Assessing the effects of movement complexity on task switching using mobile EEG. European Journal of Neuroscience, 54(12), 8175-8195.								0.5
124	Richard, C. D., Poole, J. R., McConnell, M., Meghdadi, A. H., Stevanovic-Karic, M., Rupp, G., ... & Berka, C.	2021	Alterations in electroencephalography theta as candidate biomarkers of acute cannabis intoxication. Frontiers in Neuroscience, 15, 744762.								
125	Richard, C., Karić, M. S., McConnell, M., Poole, J., Rupp, G., Fink, A., ... & Berka, C.	2021	Elevated inter-brain coherence between subjects with concordant stances during discussion of social issues. Frontiers in Human Neuroscience, 15, 611886.								
126	Rodrigues, J., Weiß, M., Hewig, J., & Allen, J. J.	2021	EPOS: EEG processing open-source scripts. Frontiers in neuroscience, 15, 660449.								
127	Rodriguez-Larios, J., de Oca, E. A. B. M., & Alaerts, K.	2021	The EEG spectral properties of meditation and mind wandering differ between experienced meditators and novices. Neuroimage, 245, 118669.								
128	Rovetti, J., Copelli, F., & Russo, F. A.	2021	Audio and visual speech emotion activate the left pre-supplementary motor area. Cognitive, Affective, & Behavioral Neuroscience, 1-13.								
129	Rudnev, V., Melnikov, M., Savelov, A., Shtark, M., & Sokhadze, E. M.	2021	. fMRI-EEG fingerprint regression model for motor cortex. NeuroRegulation, 8(3), 162-162.								

130	Rygvold, T. W., Hatlestad-Hall, C., Elvsåshagen, T., Moberget, T., & Andersson, S.	2021	Do visual and auditory stimulus-specific response modulation reflect different mechanisms of neocortical plasticity?. European Journal of Neuroscience, 53(4), 1072-1085.								
131	Sadiya, S., Alhanai, T., & Ghassemi, M. M.	2021	Artifact detection and correction in eeg data: a review. In 2021 10th International IEEE/EMBS Conference on Neural Engineering (NER) (pp. 495-498). IEEE. https://doi.org/10.1109/NER49283.2021.9441341								
132	Sæther, L. S., Roelfs, D., Moberget, T., Andreassen, O. A., Elvsåshagen, T., Jönsson, E. G., & Vaskinn, A.	2021	Exploring neurophysiological markers of visual perspective taking: Methodological considerations. International Journal of Psychophysiology, 161, 1-12.								0.2
133	Sahel, J. A., Boulanger-Scemama, E., Pagot, C., Arleo, A., Galluppi, F., Martel, J. N., ... & Roska, B.	2021	Partial recovery of visual function in a blind patient after optogenetic therapy. Nature medicine, 27(7), 1223-1229.								
134	Saravanapandian, V	2021	Electrophysiological biomarkers of neurodevelopmental disorders: Discoveries from Dup15q syndrome. University of California, Los Angeles.								
135	Saravanapandian, V., Nadkarni, D., Hsu, S. H., Hussain, S. A., Maski, K., Golshani, P., ... & Jeste, S. S.	2021	Abnormal sleep physiology in children with 15q11. 2-13.1 duplication (Dup15q) syndrome. Molecular autism, 12(1), 1-14.	0.5	0.5	0.5	0.5	0.5			
136	Sawangjai, P., Trakulruangroj, M., Boonnag, C., Piriyajitakonkij, M., Tripathy, R. K., Sudhawiyangkul, T., & Wilaiprasitporn, T	2021	EEGANet: Removal of ocular artifacts from the EEG signal using generative adversarial networks. IEEE Journal of Biomedical and Health Informatics, 26(10), 4913-4924. https://doi.org/10.1109/JBHI.2021.3131104								
137	Scanlon, J. E., Jacobsen, N. S. J., Maack, M. C., & Debener, S	2021	Does the electrode amplification style matter? A comparison of active and passive EEG system configurations during standing and walking. European Journal of Neuroscience, 54(12), 8381-8395.	0.4	0.85						
138	Semmler, C.	2021	The Use of Information about the Foreseeable Termination of a Prolonged Task to Modulate Time on Task Effects: an ERP-Study (Master's thesis, University of Twente).								

139	Seraji, M., Mohebbi, M., Safari, A., & Krekelberg, B.	2021	Multiple sclerosis reduces synchrony of the magnocellular pathway. Plos one, 16(8), e0255324.	0.7	0.7	0.7	0.7	0.7			
140	Shah, V. (	2021	Improved Segmentation for Automated Seizure Detection Using Channel-Dependent Posteriors. (Unpublished, doctoral dissertation Temple University.								
141	Sharifian, F., Schneider, D., Arnau, S., & Wascher, E.	2021	Decoding of cognitive processes involved in the continuous performance task. International Journal of Psychophysiology, 167, 57-68.								
142	Shenoy Handiru, V., Alivar, A., Hoxha, A., Saleh, S., Suviseshamuthu, E. S., Yue, G. H., & Allexandre, D.	2021	Graph-theoretical analysis of EEG functional connectivity during balance perturbation in traumatic brain injury: A pilot study. Human Brain Mapping, 42(14), 4427-4447.								
143	Sherman, D. A., Lehmann, T., Baumeister, J., Gokeler, A., Donovan, L., & Norte, G. E.	2021	External focus of attention influences cortical activity associated with single limb balance performance. Physical Therapy, 101(12), pzab223.								
144	Shi, Q., Li, Z., Zhang, L., Jiang, H., Tian, F., Zhao, Q., & Hu, B.	2021	High-speed ocular artifacts removal of multichannel EEG based on improved moment matching. Journal of Neural Engineering, 18(5), 056038. https://doi.org/10.1088/1741-2552/ac1d5a								
145	Shinagawa, K., Ito, Y., Tsuji, K., Tanaka, Y., Odaka, M., Shibata, M., ... & Umeda, S.	2021	Temporal changes in attentional resources consumed by mind-wandering that precede awareness: An ERP study. Neuroimage: Reports, 1(4), 100060.								
146	Shirazi, S. Y.	2021	Corticomuscular Adaptation to Mechanical Perturbations in a Seated Locomotor Task. (Doctoral dissertation, University of Central florida).								
147	Shumska, M.	2021	Motor Imagery EEG Classification with the SNN-based NeuCube Framework (Doctoral dissertation, University of Groningen).								
148	Simon, J. C., & Gutsell, J. N.	2021	Recognizing humanity: dehumanization predicts neural mirroring and empathic accuracy in face-to-face interactions. Social cognitive and affective neuroscience, 16(5), 463-473.	0.5	0.5				0.5		
149	Smit, D. J., Andreassen, O. A., Boomsma, D. I., Burwell, S. J., Chorlian, D. B., De	2021	Large-scale collaboration in ENIGMA-EEG: A perspective on the meta-analytic approach to link neurological and psychiatric liability genes to electrophysiological brain activity. Brain and Behavior, 11(8), e02188.								

	Geus, E. J., ... & Wright, M. J.										
150	Soghoyan, G., Ledovsky, A., Nekrashevich, M., Martynova, O., Polikanova, I., Portnova, G., ... & Sharaev, M.	2021	A Toolbox and Crowdsourcing Platform for Automatic Labeling of Independent Components in Electroencephalography. Frontiers in Neuroinformatics, 60.								
151	Solovey, E. T., & Putze, F.	2021	Improving HCI with brain input: Review, trends, and outlook. Foundations and Trends® in Human–Computer Interaction, 13(4), 298-379. http://dx.doi.org/10.1561/1100000078								
152	Song, S., & Nordin, A. D.	2021	Mobile electroencephalography for studying neural control of human locomotion. Frontiers in Human Neuroscience, 642.								
153	Stuart, S., Wagner, J., Makeig, S., & Mancini, M.	2021	Brain activity response to visual cues for gait impairment in Parkinson's disease: an EEG study. Neurorehabilitation and neural repair, 35(11), 996-1009.							0.7	
154	Sung, S. H., Kim, S., Park, B. K., Kang, D. Y., Sul, S., Jeong, J., & Kim, S. P.	2021	A Study on Facial Expression Change Detection Using Machine Learning Methods with Feature Selection Technique. Mathematics, 9(17), 2062.	0.8	0.8						
155	Tellache, M. M., Kambara, H., Koike, Y., Miyakoshi, M., & Yoshimura, N.	2021	Investigating Neural Representation of Finger-Movement Directions Using Electroencephalography Independent Components. Journal of Biomedical Science and Engineering, 14(6), 240-265.								
156	Toma, F. M., & Miyakoshi, M.	2021	Left frontal EEG power responds to stock price changes in a simulated asset bubble market. Brain Sciences, 11(6), 670.								
157	Tran, X. A., McDonald, N., Dickinson, A., Scheffler, A., Frohlich, J., Marin, A., ... & Spurling Jeste, S.	2021	Functional connectivity during language processing in 3-month-old infants at familial risk for autism spectrum disorder. European Journal of Neuroscience, 53(5), 1621-1637. https://doi.org/10.1111/ejn.15005								
158	Upshaw, J. D.	2021	The Effect of a Mindfulness-Based Intervention on Attention and Cognitive Control as a Function of Smartphone Notifications (Doctoral dissertation, University of Arkansas).								
159	Van, L. D., Tu, Y. C., Chang, C. Y., Wang, H. J., & Jung, T. P.	2021	Hardware-Oriented Memory-Limited Online Artifact Subspace Reconstruction (HMO-ASR) Algorithm. IEEE Transactions on Circuits and Systems II: Express Briefs, 68(12), 3493-3497.								
160	Vařeka, L., & Ladouce, S.	2021	Prediction of navigational decisions in the real-world: a visual P300 event-related potentials brain-computer interface. International Journal of Human–Computer Interaction, 37(14), 1375-1389.								

161	Vařeka, L., & Ladouce, S.	2021	Identification of Neuropathic Pain Severity based on Linear and Non-Linear EEG Features. In 2021 43rd Annual International Conference of the IEEE Engineering in Medicine & Biology Society (EMBC) (pp. 169-173). IEEE.								
162	Vrijdag, X. C. E.	2021	Monitoring gas narcosis in hyperbaric environments (Unpublished, doctoral dissertation, the university of Auckland).								
163	Wang, P. B.	2021	Modifying Motor Skill Learning via Neuromodulation of Frontoparietal Networks (Doctoral dissertation, Arizona State University).								
164	Wang, W. E., Ho, R. L., Gatto, B., van der Veen, S. M., Underation, M. K., Thomas, J. S., ... & Coombes, S. A.	2021	Cortical dynamics of movement-evoked pain in chronic low back pain. The Journal of Physiology, 599(1), 289-305.								
165	Wang, Y., Bai, Y., Xia, X., Niu, Z., Yang, Y., He, J., & Li, X.	2021	Comparison of synchrosqueezing transform to alternative methods for time-frequency analysis of TMS-evoked EEG oscillations. Biomedical Signal Processing and Control, 70, 102975. https://doi.org/10.1016/j.bspc.2021.102975								
166	Wiemer, J., Leimeister, F., & Pauli, P.	2021	Subsequent memory effects on event-related potentials in associative fear learning. Social Cognitive and Affective Neuroscience, 16(5), 525-536.	0.7	0.7	0.7	0.7	0.7			
167	Willoughby, T., Heffer, T., van Noordt, S., Desjardins, J., Segalowitz, S., & Schmidt, L.	2021	An ERP investigation of children and adolescents' sensitivity to wins and losses during a peer observation manipulation. Developmental Cognitive Neuroscience, 51, 100995.								
168	Wilson, N., Gorji, H. T., VanBree, J., Hoffmann, B., Tavakolian, K., & Petros, T.	2021	Identifying opportunities for augmented cognition during live flight scenario: an analysis of pilot mental workload using EEG. In 94th International Symposium on Aviation Psychology (p. 444).								
169	Wisniewski, M. G., Zakrzewski, A. C., Bell, D. R., & Wheeler, M.	2021	EEG power spectral dynamics associated with listening in adverse conditions. Psychophysiology, 58(9), e13877.								
170	Wu, M., Luo, B., Yu, Y., Li, X., Gao, J., Li, J., ... & Riecke, L	2021	Non-Invasive Rhythmic Musical-Electric Trigeminal Nerve Stimulation Improves Consciousness in Patients with Disorders of Consciousness. https://doi.org/10.21203/rs.3.rs-256846/v1								
171	Wunderlich, A., & Gramann, K.	2021	Eye movement-related brain potentials during assisted navigation in real-world environments. European Journal of Neuroscience, 54(12), 8336-8354.								
172	Xu, B., Deng, L., Zhang, D., Xue, M.,	2021	Electroencephalogram Source Imaging and Brain Network Based Natural Grasps Decoding. Frontiers in Neuroscience, 1620.								

	Li, H., Zeng, H., & Song, A.										
173	Yano, M., & Koizumi, M.	2021	The role of discourse in long-distance dependency formation. Language, Cognition and Neuroscience, 36(6), 711-729.		0.9						
174	Yao, Z., Lin, X., & Hu, X.	2021	Optimistic amnesia: how online and offline processing shape belief updating and memory biases in immediate and long-term optimism biases. Social Cognitive and Affective Neuroscience, 16(5), 453-462.								
175	Zaky, M. H., Shoorangiz, R., Poudel, G. R., Yang, L., & Jones, R. D.	2021	Investigating the neural signature of microsleeps using EEG. In 2021 43rd Annual International Conference of the IEEE Engineering in Medicine & Biology Society (EMBC) (pp. 6293-6296). IEEE.								
176	Zapała, D., Iwanowicz, P., Francuz, P., & Augustynowicz, P.	2021	Handedness effects on motor imagery during kinesthetic and visual-motor conditions. Scientific Reports, 11(1), 13112.								
177	Zeng, Y., Fiorentino, R., & Zhang, J.	2021	Electrophysiological signatures of perceiving alternated tone in Mandarin Chinese: Mismatch negativity to underlying tone conflict. Frontiers in Psychology, 12, 735593.								
178	Zhang, H., Wei, C., Zhao, M., Liu, Q., & Wu, H.	2021	A novel convolutional neural network model to remove muscle artifacts from EEG. In ICASSP 2021-2021 IEEE International Conference on Acoustics, Speech and Signal Processing (ICASSP) (pp. 1265-1269). IEEE.								
179	Zhang, H., Zhao, M., Wei, C., Mantini, D., Li, Z., & Liu, Q.	2021	Eegdenoisenet: A benchmark dataset for deep learning solutions of eeg denoising. Journal of Neural Engineering, 18(5), 056057.								
180	Zhang, J., Xu, Z., Zhou, Y., Wang, P., Fu, P., Xu, X., & Zhang, D.	2021	An empirical comparative study on the two methods of eliciting singers' emotions in singing: Self-imagination and VR training. Frontiers in Neuroscience, 15, 693468.								
181	Zhao, M., Bonassi, G., Guarnieri, R., Pelosin, E., Nieuwboer, A., Avanzino, L., & Mantini, D.	2021	A multi-step blind source separation approach for the attenuation of artifacts in mobile high-density electroencephalography data. Journal of neural engineering, 18(6), 066041.								
182	Zheng, J., Liang, M., Sinha, S., Ge, L., Yu, W., Ekstrom, A., & Hsieh, F.	2021	Time-frequency analysis of scalp EEG with Hilbert-Huang transform and deep learning. IEEE Journal of biomedical and health informatics, 26(4), 1549-1559. https://doi.org/10.1109/JBHI.2021.3110267								
183	Zhu, X., Oh, Y., Chesebrough, C., Zhang, F., & Kounios, J.	2021	Pre-stimulus brain oscillations predict insight versus analytic problem-solving in an anagram task. Neuropsychologia, 162, 108044.								

184	Zuure, M. B., & Cohen, M. X.	2021	Narrowband multivariate source separation for semi-blind discovery of experiment contrasts. Journal of Neuroscience Methods, 350, 109063.								

2022

Index	Author	Year	Journal information	muscle	eye	heart	line noise	channel noise	other	brain	brain removed
1	Aamodt, A., Nilsen, A. S., Markhus, R., Kusztor, A., HasanzadehMoghadam, F., Kauppi, N., Thürer, B., Storm, J. F., & Juel, B. E.	2022	EEG Lempel-Ziv complexity varies with sleep stage, but does not seem to track dream experience. Frontiers in Human Neuroscience, 16. https://doi.org/10.3389/fnhum.2022.987714			1.0 ?					0.15
2	Abreu, R., Soares, J. F., Lima, A. C., Sousa, L., Batista, S., Castelo-Branco, M., & Duarte, J. V.	2022	Optimizing EEG source reconstruction with concurrent fMRI-derived spatial priors. Brain Topography, 35(3), 282-301. https://doi.org/10.1007/s10548-022-00891-3								
3	Acar, Z. A., & Makeig, S.	2022	Evaluation of skull conductivity using SCALE head tissue conductivity estimation using EEG. In 2022 44th Annual International Conference of the IEEE Engineering in Medicine & Biology Society (EMBC) (pp. 4826-4829). IEEE. https://doi.org/10.1109/EMBC48229.2022.9872004								
4	An, S., Oh, S. J., Jun, S. B., & Sung, J. E.	2022	Aging-Related Dissociation of Spatial and Temporal N400 in Sentence-Level Semantic Processing: Evidence From Source Analyses. Frontiers in Aging Neuroscience, 14. https://doi.org/10.3389/fnagi.2022.877235								
5	Arai, T., & Nittono, H.	2022	Cosmetic makeup enhances facial attractiveness and affective neural responses. Plos one, 17(8), e0272923. https://doi.org/10.1371/journal.pone.0272923								
6	Assecondi, S., Villa-Sánchez, B., & Shapiro, K.	2022	Event-related potentials as markers of efficacy for combined working memory training and transcranial direct current stimulation regimens: A proof-of-concept study. Frontiers in Systems Neuroscience, 16. https://doi.org/10.3389/fnsys.2022.837979	0.9	0.9	0.9	0.9	0.9			
7	Bagdasarov, A., Roberts, K., Bréchet, L., Brunet, D., Michel, C. M., & Gaffrey, M. S.	2022	Spatiotemporal dynamics of EEG microstates in four-to eight-year-old children: Age-and sex-related effects. Developmental cognitive neuroscience, 57, 101134. https://doi.org/10.1016/j.dcn.2022.101134								
8	Bailey, N. W., Biabani, M., Hill, A. T., Miljevic, A., Rogasch, N. C.,	2022	Introducing RELAX (the Reduction of Electroencephalographic Artifacts): A fully automated pre-processing pipeline for cleaning EEG data - Part 1:								

	McQueen, B., Murphy, O. W., & Fitzgerald, P. B.		Algorithm and Application to Oscillations. https://doi.org/10.1101/2022.03.08.483548. COVID-19 SARS-CoV-2 preprints from medRxiv and bioRxiv								
9	Bakhtyari, M., & Mirzaei, S.	2022	ADHD detection using dynamic connectivity patterns of EEG data and ConvLSTM with attention framework. Biomedical Signal Processing and Control Volume 76, July 2022, 103708. https://doi.org/10.1016/j.bspc.2022.103708								
10	Balestrieri, E., Michel, R., & Busch, N. A.	2022	Alpha-band lateralization and microsaccades elicited by exogenous cues do not track attentional orienting. bioRxiv, 2022-12. https://doi.org/10.1101/2022.12.12.520080	0.5	0.5	0.5	0.5	0.5	0.5		0.1
11	Banea, O. C.	2022	Towards neurophysiological biomarkers to assess repetitive transcranial magnetic stimulation treatment for patients with schizophrenia and auditory verbal hallucinations. https://hdl.handle.net/20.500.11815/2881								
12	Banea, O. C., Dos Santos, L. G. B., Marcu, S., Stefánnson, S. B., Wassermann, E. M., Ívarsson, E., ... & Gargiulo, P.	2022	Network signatures of rTMS treatment in patients with schizophrenia and auditory verbal hallucination during an auditory-motor task using HD-EEG. Schizophrenia research, 243, 310-314. https://doi.org/10.1016/j.schres.2021.06.002								
13	Bao, Z., & Frewen, P.	2022	Sense of self in mind and body: an eLORETA-EEG study. Neuroscience of Consciousness, 2022(1), niac017. https://doi.org/10.1093/nc/niac017							0.7	
14	Barros, C., Pereira, A. R., Sampaio, A., Buján, A., & Pinal, D.	2022	Frontal alpha asymmetry and negative mood: a cross-sectional study in older and younger adults. Symmetry, 14(8), 1579. https://doi.org/10.3390/sym14081579								0.2
15	Bayot, M., Gérard, M., Derambure, P., Dujardin, K., Defebvre, L., Betrouni, N., & Delval, A.	2022	Functional networks underlying freezing of gait: a resting-state electroencephalographic study. Neurophysiologie Clinique, 52(3), 212-222. https://doi.org/10.1016/j.neucli.2022.03.003								
16	Begau, A., Arnau, S., Klatt, L. I., Wascher, E., & Getzmann, S.	2022	Using visual speech at the cocktail-party: CNV evidence for early speech extraction in younger and older adults. Hearing Research, 426, 108636. https://doi.org/10.1016/j.heares.2022.108636	0.3	0.3	0.3					0.3
17	Begau, A., Klatt, L. I., Schneider, D., Wascher, E., & Getzmann, S.	2022	The role of informational content of visual speech in an audiovisual cocktail party: Evidence from cortical oscillations in young and old participants. European Journal of Neuroscience, 56(8), 5215-5234. https://doi.org/10.1111/ejn.15811	0.3	0.3	0.3	0.3		0.3		0.3
18	Begau, A., Klatt, L. I., Schneider, D., Wascher, E., & Getzmann, S.	2022	Age influences audiovisual speech processing in multi-talker scenarios–Evidence from cortical oscillations.	0.3	0.3	0.3	0.3	0.3	0.3	0.3	

			bioRxiv, 2022-02. https://doi.org/10.1101/2022.02.23.481314								
19	Berto, M., Ricciardi, E., Pietrini, P., & Bottari, D.	2022	Distinct cortical responses to Auditory Statistics: pre-attentive discriminations based on local and global representations. bioRxiv, 2022-03. https://doi.org/10.1101/2022.03.17.484757		0.95						
20	Bhattacharyya, A., Verma, A., Ranta, R., & Pachori, R. B.	2022	Ocular Artifacts Elimination from Multivariate EEG Signal using Frequency-Spatial Filtering. IEEE Transactions on Cognitive and Developmental Systems. https://doi.org/10.1109/TCDS.2022.3226775								
21	Bigelow, F. J., Clark, G. M., Lum, J. A. G., & Enticott, P. G.	2022	Facial emotion processing and language during early-to-middle childhood development: An event related potential study. Developmental Cognitive Neuroscience Volume 53, February 2022, 101052. https://doi.org/10.1016/j.dcn.2021.101052								
22	Bigelow, F. J., Clark, G. M., Lum, J. A., & Enticott, P. G.	2022	Moral content influences facial emotion processing development during early-to-middle childhood. Neuropsychologia, 176, 108372. https://doi.org/10.1016/j.neuropsychologia.2022.108372								
23	Bilucaglia, M., Laureanti, R., Circi, R., Zito, M., Bellati, M., Fici, A., Rivetti, F., Mainardi, L. T., & Russo, V.	2022	Spectral differences in resting-state EEG associated to individual Emotional Styles. In 2022 44th Annual International Conference of the IEEE Engineering in Medicine & Biology Society (EMBC) (pp. 4052-4055). IEEE. https://doi.org/10.1109/EMBC48229.2022.9871191								0.7
24	Bin, Z.	2022	Investigation on the neural oscillatory mechanisms of speech production and comprehension								
25	Blundon, E. G., Gallagher, R. E., & Ward, L. M.	2022	Resting state network activation and functional connectivity in the dying brain. Clinical Neurophysiology, 135, 166-178. https://doi.org/10.1016/j.clinph.2021.10.018								
26	Blundon, E. G., Gallagher, R., DiMaio, L., & Ward, L. M.	2022	Electrophysiological evidence of sustained attention to music among conscious participants and unresponsive hospice patients at the end of life. Clinical Neurophysiology, 139, 9-22. https://doi.org/10.1016/j.clinph.2022.03.018								
27	Bower, I. S., Clark, G. M., Tucker, R., Hill, A. T., Lum, J. A., Mortimer, M. A., & Enticott, P. G.	2022	Built environment color modulates autonomic and EEG indices of emotional response. Psychophysiology, 59(12), e14121. https://doi.org/10.1111/psyp.14121								0.3
28	Bower, I. S., Clark, G. M., Tucker, R., Hill, A. T., Lum, J. A., Mortimer, M. A., & Enticott, P. G.	2022	Enlarged interior built environment scale modulates high frequency EEG oscillations. Eneuro. https://doi.org/10.1523/ENEURO.0104-22.2022								0.3
29	Bower, I. S., Tucker, R., & Enticott, P.	2022	Architecture with Feeling Research Study.								

30	Bujan, A., Sampaio, A., & Pinal, D.	2022	Resting-state electroencephalographic correlates of cognitive reserve: Moderating the age-related worsening in cognitive function. Frontiers in Aging Neuroscience, 988. https://doi.org/10.3389/fnagi.2022.854928								
31	Callara, A. L., Cecchetto, C., Dal Bò, E., Citi, L., Gentili, C., Vanello, N., Scilingo, E. P., & Greco, A.	2022	Human body odors of happiness and fear modulate the late positive potential component during neutral face processing: a preliminary ERP study on healthy subjects. In 2022 44th Annual International Conference of the IEEE Engineering in Medicine & Biology Society (EMBC) (pp. 4093-4096). IEEE. https://doi.org/10.1109/EMBC48229.2022.9871495	1.0?	1.0?	1.0?	1.0?	1.0?	1.0?		
32	Campos-Arteaga, G., Araneda, A., Ruiz, S., Rodríguez, E., & Sitaram, R.	2022	Classifying brain states and pupillary responses associated with the processing of old and new information. International Journal of Psychophysiology, 176, 129-141. https://doi.org/10.1016/j.ijpsycho.2022.04.004								
33	Carlos, B. M., Campos, B. M., Alvim, M. K., & Castellano, G.	2022	Brain Connectivity Measures in EEG-Based Biometry for Epilepsy Patients: A Pilot Study. In Computational Neuroscience: Third Latin American Workshop, LAWCN 2021, São Luís, Brazil, December 8–10, 2021, Revised Selected Papers (pp. 155-169). Cham: Springer International Publishing. https://doi.org/10.1007/978-3-031-08443-0_10								
34	Casaponsa, A., García-Guerrero, M. A., Martínez, A., & Ojeda, N.	2022	Electrophysiological evidence for a Whorfian double dissociation of categorical perception across two languages.								
35	Cassani, C. M., Coelli, S., Calcagno, A., Temporiti, F., Mandaresu, S., Gatti, R., ... & Bianchi, A. M.	2022	Selecting a pre-processing pipeline for the analysis of EEG event-related rhythms modulation. In 2022 44th Annual International Conference of the IEEE Engineering in Medicine & Biology Society (EMBC) (pp. 4044-4047). IEEE. https://doi.org/10.1109/EMBC48229.2022.9871394								
36	Celli, M., Mazzonetto, I., Zangrossi, A., Bertoldo, A., Cona, G., & Corbetta, M.	2022	One-year-later spontaneous EEG features predict visual exploratory human phenotypes. Communications Biology, 5(1), 1361. https://doi.org/10.1038/s42003-022-04294-9								
37	Chen, H., He, H., Sun, S., Li, J., Shao, X., Li, J., Li, X., & Hu, B.	2022	Weight-based Channel-model Matrix Framework: a reasonable solution for EEG-based cross-dataset emotion recognition. arXiv preprint arXiv:2209.05849. https://doi.org/10.48550/arXiv.2209.05849								
38	Chen, H., Zhang, H., Liu, C., Chai, Y., & Li, X.	2022	An outlier detection-based method for artifact removal of few-channel EEGs. Journal of Neural Engineering, 19(5), 056028. https://doi.org/10.1088/1741-2552/ac954d								

39	Chen, I. C., Chang, C. L., Chang, M. H., & Ko, L. W.	2022	Atypical functional connectivity during rest and task-related dynamic alteration in young children with attention deficit hyperactivity disorder: An analysis using the phase-locking value. Psychiatry and Clinical Neurosciences, 76(6), 235-245. https://doi.org/10.1111/pcn.13344								
40	Chen, I. C., Chen, C. L., Chang, C. H., Fan, Z. C., Chang, Y., Lin, C. H., & Ko, L. W.	2022	Task-Rate-Related Neural Dynamics Using Wireless EEG to Assist Diagnosis and Intervention Planning for Preschoolers with ADHD Exhibiting Heterogeneous Cognitive Proficiency. Journal of Personalized Medicine, 12(5), 731. https://doi.org/10.3390/jpm12050731								
41	Chen, K., Wang, R., Huang, J., Gao, F., Yuan, Z., Qi ,Y., & Wu, H.	2022	A resource for assessing dynamic binary choices in the adult brain using EEG and mouse-tracking. Scientific Data volume 9, Article number: 416 (2022). https://doi.org/10.1038/s41597-022-01538-5								
42	Chen, P. H., & Rau, P. L. P.	2022	Alpha Oscillations in Parietal and Parietooccipital Explaining How Boredom Matters Prospective Memory. Frontiers in Neuroscience, 16. https://doi.org/10.3389/fnins.2022.789031		0.5						
43	Chen, X., Li, C., Liu, A., McKeown, M. J., Qian, R., & Wang, Z. J.	2022	Toward Open-World Electroencephalogram Decoding Via Deep Learning: A comprehensive survey. IEEE Signal Processing Magazine (Volume: 39, Issue: 2, March 2022) DOI: 10.1109/MSP.2021.3134629								
44	Cheng, B., Wunderlich, A., Gramann, K., Lin, E., & Fabrikant, S. I.	2022	The effect of landmark visualization in mobile maps on brain activity during navigation: A virtual reality study. Frontiers in Virtual Reality, 3, 981625. https://doi.org/10.3389/frvir.2022.981625		0.7						
45	Cheng, T. H. Z., Creel, S. C., & Iversen, J. R.	2022	How do you feel the rhythm: Dynamic motor-auditory interactions are involved in the imagination of hierarchical timing. Journal of Neuroscience, 42(3), 500-512. https://doi.org/10.1523/JNEUROSCI.1121-21.2021							0.4	
46	Chiang, H. S., Lydon, E. A., Kraut, M. A., Hart Jr, J., & Mudar, R. A.	2022	Differences in EEG oscillations between normal aging and mild cognitive impairment during semantic memory retrieval. European Journal of Neuroscience. https://doi.org/10.1111/ejn.16001	0.7	0.7	0.7					
47	Chiang, H. S., Motes, M., Kraut, M., Vanneste, S., & Hart, J.	2022	High-definition transcranial direct current stimulation modulates theta response during a Go-NoGo task in traumatic brain injury. Clinical Neurophysiology, 143, 36-47. https://doi.org/10.1016/j.clinph.2022.08.015	0.7	0.7	0.7					
48	Chuang, C. H., Chang, K. Y., Huang, C. S., & Jung, T. P.	2022	IC-U-Net: a U-Net-based denoising autoencoder using mixtures of independent components for automatic EEG artifact removal. NeuroImage, 263, 119586. https://doi.org/10.1016/j.neuroimage.2022.119586							0.8	

49	Chung, Y. S., Bagdasarov, A., & Gaffrey, M. S.	2022	Sex-specific association between emotion negativity and neural processing of reward feedback in young children. bioRxiv, 2022-06. https://doi.org/10.1101/2022.06.20.496831		0.7						
50	Chung, Y. S., Bagdasarov, A., Gaffrey, M. S., Chung, Y. S., Bagdasarov, A., & Gaffrey, M. S.	2022	Sex differences in the neural processing of reward feedback in young children.		0.7						
51	Contier, F., Weymar, M., Wartenburger, I., & Rabovsky, M.	2022	Sustained attention as measured by reaction time variability is a strong modulator for the P600, but not the N400. Journal of Cognitive Neuroscience, 34(12), 2297-2310.								
52	Copelli, F., Rovetti, J., Ammirante, P., & Russo, F. A.	2022	Human mirror neuron system responsivity to unimodal and multimodal presentations of action. Experimental Brain Research, 240(2), 537-548. https://doi.org/10.1007/s00221-021-06266-7								
53	Cristaldi, F. D. P., Buodo, G., Duma, G. M., Sarlo, M., & Mento, G.	2022	Unbalanced functional connectivity at rest affects the ERP correlates of affective prediction in high intolerance of uncertainty individuals: A high density EEG investigation. International Journal of Psychophysiology, 178, 22-33. https://doi.org/10.1016/j.ijpsycho.2022.06.006								
54	Cruz-Garza, J. G., Darfler, M., Rounds, J. D., Gao, E., & Kalantari, S.	2022	EEG-based investigation of the impact of room size and window placement on cognitive performance. Journal of Building Engineering, 53, 104540. https://doi.org/10.1016/j.jobe.2022.104540								
55	Darfler, M., Cruz-Garza, J. G., & Kalantari, S.	2022	An EEG-based investigation of the effect of perceived observation on visual memory in virtual environments. Brain Sciences, 12(2), 269. https://doi.org/10.3390/brainsci12020269	0.9	0.9	0.9		0.9			
56	Davis, M-C., Hill, A. T., Fitzgerald, P. B., Stout, J. C., & Hoy, K. E.	2022	Motivationally salient cue processing measured using the monetary incentive delay (MID) task with electroencephalography (EEG): A potential marker of apathy in Huntington's disease. Neuropsychologia Volume 177, 15 December 2022, 108426. https://doi.org/10.1016/j.neuropsychologia.2022.108426								
57	Davis, M. C.	2022	Chapter Eight: Effect of tACS on motivationally salient cue processing. Investigating transcranial alternating current stimulation for apathy in Huntington's disease, 233.								
58	Davis, M. C., Hill, A. T., Fitzgerald, P. B., Bailey, N. W., Stout, J. C., & Hoy, K. E.	2022	Examining neurophysiological markers of apathy and processing speed in late premanifest and early-stage manifest Huntington's disease. medRxiv, 2022-08. https://doi.org/10.1101/2022.08.09.22278610								

59	Davisa, M. C., Hillc, A. T., Fitzgeralda, P. B., Baileya, N. W., Stoutd, J. C., & Hoya, K. E.	2022	Investigating resting state neurophysiological markers of apathy and processing speed in prodromal and early-stage manifest Huntington's disease.								
60	Debnath, R., & Wetzel, N.	2022	Processing of task-irrelevant sounds during typical everyday activities in children. Developmental Psychobiology, 64(7), e22331. https://doi.org/10.1002/dev.22331								
61	del Mar Cordero, M., Denis-Noël, A., Spinelli, E., & Meunier, F.	2022	Neural correlates of acoustic and semantic cues during speech segmentation in French. In Interspeech 2022 (pp. 4058-4062). ISCA. https://doi.org/10.21437/Interspeech.2022-10986	0.8	0.8			0.8			
62	Delorme, A.	2022	EEG is better left alone. bioRxiv, 2022-12. Scientific Reports volume 13, Article number: 2372 (2023) https://doi.org/10.1038/s41598-023-27528-0	0.9	0.9						
63	Dercksen, T. T., Widmann, A., Scharf, F., & Wetzel, N.	2022	Sound omission related brain responses in children. Developmental Cognitive Neuroscience, 53, 101045. https://doi.org/10.1016/j.dcn.2021.101045								
64	Di Dona, G., Scaltritti, M., & Sulpizio, S.	2022	Formant-invariant voice and pitch representations are pre-attentively formed from constantly varying speech and non-speech stimuli. European Journal of Neuroscience, 56(3), 4086-4106. https://doi.org/10.1111/ejn.15730								
65	Diachenko, M., Houtman, S. J., Juarez-Martinez, E. L., Ramautar, J. R., Weiler, R., Mansvelder, H. D., Bruining, H., Bloem, P., & Linkenkaer-Hansen, K.	2022	Improved manual annotation of EEG signals through convolutional neural network guidance. Eneuro. https://doi.org/10.1523/ENEURO.0160-22.2022								
66	Donnelly, N. A., Bartsch, U., Moulding, H. A., Eaton, C., Marston, H., Hall, J. H., Hall, J., Owen, M. J., van den Bree, M. B. M., & Jones, M. W.	2022	Sleep EEG in young people with 22q11. 2 deletion syndrome: A cross-sectional study of slow-waves, spindles and correlations with memory and neurodevelopmental symptoms. Elife, 11, e75482. https://doi.org/10.7554/eLife.75482	1.0?	1.0?	1.0?	1.0?	1.0?	1.0?		
67	Dück, K., Overmeyer, R., Mohr, H., & Endrass, T.	2022	Are electrophysiological correlates of response inhibition linked to impulsivity and compulsivity? A machine-learning analysis of a Go/Nogo task. Psychophysiology, e14310. https://doi.org/10.1111/psyp.14310								
68	Duraisingam, A., Soria, D., & Palaniappan, R.	2022	Event-Related Neural Responses to Short Term Habituation of Visual Food and Non-Food Stimuli.	0.7	0.7	0.7	0.7	0.7			

69	Elsabbagh, M.	2022	Inter-trial theta phase consistency during face processing in infants is associated with later emerging autism. https://doi.org/10.1002/aur.2701								
70	Faustino Lacerda de Souza, R., Borges de Araujo Lima, L. A., Almeida Silveira Mendes, T. M., Soares Brandão, D., Andrés Laplagne, D., & Cordeiro de Sousa, M. B.	2022	Implicit motor imagery performance and cortical activity throughout the menstrual cycle. Scientific Reports, 12(1), 21385. https://doi.org/10.1038/s41598-022-25766-2								
71	Forbes, O., Schwenn, P. E., Wu, P. P. Y., Santos-Fernandez, E., Xie, H. B., Lagopoulos, J., McLoughlin, L. T., Sacks, D. D., Mengersen, K., & Hermens, D. F.	2022	EEG-based clusters differentiate psychological distress, sleep quality and cognitive function in adolescents. Biological Psychology, 173, 108403. https://doi.org/10.1016/j.biopsycho.2022.108403								
72	Forschack, N., Gundlach, C., Hillyard, S., & Müller, M. M.	2022	Electrophysiological evidence for target facilitation without distractor suppression in two-stimulus search displays. Cerebral Cortex, Volume 32, Issue 17, 1 September 2022, Pages 3816–3828, https://doi.org/10.1093/cercor/bhab450								
73	Forschack, N., Gundlach, C., Hillyard, S., & Müller, M. M.	2022	Attentional capture is modulated by stimulus saliency in visual search as evidenced by event-related potentials and alpha oscillations. Attention, Perception, & Psychophysics, 85, 685-704. https://doi.org/10.3758/s13414-022-02629-6							0.42	
74	Fraschini, M., La Cava, S. M., Rodriguez, G., Vitale, A., & Demuru, M.	2022	Scorepochs: a computer-aided scoring tool for resting-state M/EEG epochs. Sensors, 22(8), 2853. https://doi.org/10.3390/s22082853							0.2	
75	Galdino, L. B., Fernandes, T., Schmidt, K. E., & Santos, N. A.	2022	Altered brain connectivity during visual stimulation in schizophrenia. Experimental Brain Research, 1-11. https://doi.org/10.1007/s00221-022-06495-4								
76	Galdino, L., Silva, G. M., Bonifacio, T., Santos, N. A., & Orme-Johnson, D.	2022	Effects of the Automatic Self-Transcending Meditation on cognition and mental states in the EEG, skin conductance and behavioral performance: a pilot study. bioRxiv, 2022-10. https://doi.org/10.1101/2022.10.11.511756								
77	Gao, F.	2022	The When, Where, and How of Chinese Morphological Processing in the Human Brain: Evidence from EEG-fNIRS (Doctoral dissertation, University of Macau).								
78	García-Pretelt, F. J., Suárez-Relevo, J. X., Aguillon-Niño, D. F.,	2022	Automatic Classification of Subjects of the PSEN1-E280A Family at Risk of Developing Alzheimer's Disease Using Machine Learning and Resting State								

	Lopera-Restrepo, F. J., Ochoa-Gómez, J. F., & Tobón-Quintero, C. A.		Electroencephalography. Journal of Alzheimer's Disease, 87(2), 817-832. https://doi.org/10.3233/JAD-210148								
79	Gehrke, L., Lopes, P., Klug, M., Akman, S., & Gramann, K.	2022	Neural sources of prediction errors detect unrealistic VR interactions. Journal of Neural Engineering, 19(3), 036002.								
80	Gérard, M., Bayot, M., Derambure, P., Dujardin, K., Defebvre, L., Betrouni, N., & Delval, A.	2022	EEG-based functional connectivity and executive control in patients with Parkinson’s disease and freezing of gait. Clinical Neurophysiology, 137, 207-215. https://doi.org/10.1016/j.clinph.2022.01.128								
81	Getzmann, S., Arnau, S., Gajewski, P. D., & Wascher, E.	2022	When long appears short: Effects of auditory distraction on event-related potential correlates of time perception. European journal of neuroscience, 55(1), 121-137. https://doi.org/10.1111/ejn.15553								
82	Gold, M. C., Yuan, S., Tirrell, E., Kronenberg, E. F., Kang, J. W. D., Hindley, L., Scherif, M., Brown, J. C., & Carpenter, L. L.	2022	Large-scale EEG neural network changes in response to therapeutic TMS. Brain Stimulation, 15(2), 316-325. https://doi.org/10.1016/j.brs.2022.01.007								
83	Granerud, G., Elvsåshagen, T., Arntzen, E., Juhasz, K., Emilsen, N. M., Sønderby, I. E., ... & Malt, E. A.	2022	A family study of symbolic learning and synaptic plasticity in autism spectrum disorder. Frontiers in Human Neuroscience, 16. https://doi.org/10.3389/fnhum.2022.950922								
84	Guevara, J. E.	2022	Evaluating Physiological Correlates of Mental Effort during Cognitive Demand (Doctoral dissertation, The University of Utah).								
85	Harmening, N., Klug, M., Gramann, K., & Miklody, D.	2022	HArtMuT—modeling eye and muscle contributors in neuroelectric imaging.<br>Citation Nils Harmening et al 2022 J. Neural Eng. 19 066041<br>DOI 10.1088/1741-2552/aca8ce<br>.								
86	Hassall, C. D., Yan, Y., & Hunt, L. T.	2022	The Neural Correlates of Continuous Feedback Processing. bioRxiv, 2022-10. https://doi.org/10.1101/2022.10.06.511117		0.8						
87	Hatlestad-Hall, C., Rygvold, T. W., & Andersson, S.	2022	BIDS-structured resting-state electroencephalography (EEG) data extracted from an experimental paradigm. Data in Brief, 45, 108647. https://doi.org/10.1016/j.dib.2022.108647								

88	Hervault, M., Zanone, P. G., Buisson, J. C., & Huys, R.	2022	Multiple Brain Sources Are Differentially Engaged in the Inhibition of Distinct Action Types. Journal of Cognitive Neuroscience, 34(2), 258-272. https://doi.org/10.1162/jocn_a_01794								
89	Hervault, M., Zanone, P. G., Buisson, J. C., & Huys, R.	2022	Hold your horses: Differences in EEG correlates of inhibition in cancelling and stopping an action. Neuropsychologia, 172, 108255. https://doi.org/10.1016/j.neuropsychologia.2022.108255								
90	Hill, A. T., Clark, G. M., Bigelow, F. J., Lum, J. A. G., & Enticott, P. G.	2022	Periodic and aperiodic neural activity displays age-dependent changes across early-to-middle childhood. Developmental Cognitive Neuroscience Volume 54, April 2022, 101076. https://doi.org/10.1016/j.dcn.2022.101076								
91	Hill, A. T., Van Der Elst, J., Bigelow, F. J., Lum, J. A., & Enticott, P. G.	2022	Right anterior theta connectivity predicts autistic social traits in typically developing children. Biological Psychology, 175, 108448. https://doi.org/10.1016/j.biopsycho.2022.108448								
92	Hilton, C., Raddatz, L., & Gramann, K.	2022	A general spatial transformation process? Assessing the neurophysiological evidence on the similarity of mental rotation and folding. Neuroimage: Reports, 2(2), 100092. https://doi.org/10.1016/j.ynirp.2022.100092		0.7						
93	Hollenstein, N., Tröndle, M., Plomecka, M., Kiegeland, S., Özyurt, Y., Jäger, L. A., & Langer, N.	2022	The ZuCo Benchmark on Cross-Subject Reading Task Classification with EEG and Eye-Tracking Data. bioRxiv, 2022-03. https://doi.org/10.1101/2022.03.08.483414	0.8	0.8	0.8	0.8	0.8			
94	HOSGÖREN, B.	2022	EEG Correlates of Spatial Navigation in Patients with Right Hippocampal Lesion: A Mobile Brain/Body Imaging (MoBI) Study. https://hdl.handle.net/20.500.12608/30630								
95	Hsu, S. H., Lin, Y., Onton, J., Jung, T-P., & Makeig, S.	2022	Unsupervised learning of brain state dynamics during emotion imagination using high-density EEG. NeuroImage Volume 249, 1 April 2022, 118873. https://doi.org/10.1016/j.neuroimage.2022.118873								
96	Hui, Y. L. K.	2022	Emotional and cognitive processes associated with the statements of motivational interviewing. https://theses.lib.polyu.edu.hk/handle/200/11637								
97	Isbell, E., & Grammer, J. K.	2022	Event-related potentials data quality in young children: Standardized measurement error of ERN and Pe. Developmental Psychobiology, 64(4), e22245. https://doi.org/10.1002/dev.22245		0.7						0.05
98	Ismail, L., Karwowski, W., Farahani, F. V., Rahman, M., Alhujailli,	2022	Modeling Brain Functional Connectivity Patterns during an Isometric Arm Force Exertion Task at Different Levels of Perceived Exertion: A Graph Theoretical Approach. Brain								

	A., Fernandez-Sumano, R., & Hancock, P. A.		Sciences, 12(11), 1575. https://doi.org/10.3390/brainsci12111575								
99	Iwama, S., Yanagisawa, T., Hirose, R., & Ushiba, J.	2022	Beta rhythmicity in human motor cortex reflects neural population coupling that modulates subsequent finger coordination stability. Communications biology, 5(1), 1375. https://doi.org/10.1038/s42003-022-04326-4								
100	Iwama, S., Zhang, Y., & Ushiba, J.	2022	De novo brain-computer interfacing deforms manifold of populational neural activity patterns in human cerebral cortex. Eneuro, 9(6). https://doi.org/10.1523/ENEURO.0145-22.2022								
101	Jeon, J. H., &Cai, H.	2022	Multi-class classification of construction hazards via cognitive states assessment using wearable EEG. Advanced Engineering Informatics Volume 53, August 2022, 101646. https://doi.org/10.1016/j.aei.2022.101646	0.8	0.8	0.8	0.8	0.8	0.8		
102	Jeong, E., Cha, K. S., Shin, H. R., Kim, E. Y., Jun, J. S., Kim, T. J., Byun, J-L., Shin, J-W., Sunwoo, J-S., & Jung, K. Y.	2022	Alerting network alteration in isolated rapid eye movement sleep behavior disorder patients with mild cognitive impairment. Sleep Medicine, 89, 10-18. https://doi.org/10.1016/j.sleep.2021.11.002								
103	Jia, Y. C., Ding, F. Y., Cheng, G., Liu, Y., Yu, W., Zou, Y., & Zhang, D. J.	2022	Infants' neutral facial expressions elicit the strongest initial attentional bias in adults: Behavioral and electrophysiological evidence. Psychophysiology, 59(1), e13944. https://doi.org/10.1111/psyp.13944	0.9	0.9	0.9	0.9	0.9			
104	John, A. R., Singh, A. K., Do, T. T. N., Eidels, A., Nalivaiko, E., Gavgani, A. M., Brown, S., Bennett, M., Lai, S., Simpson, A. M., Gustin, S. M., Double, K., Walker, F. R., Kleitman, S., Morley, J., & Lin, C. T.	2022	Unraveling the Physiological Correlates of Mental Workload Variations in Tracking and Collision Prediction Tasks. IEEE Transactions on Neural Systems and Rehabilitation Engineering, 30, 770-781. https://doi.org/10.1109/TNSRE.2022.3157446	1.0?	1.0?	1.0?	1.0?	1.0?	1.0?		
105	Josphineleela, R., Marcus, M., Kistan, A., Anitha, M., & Uma, K.	2022	PHYSIOLOGICAL EMOTION RECOGNITION BY MEANS OF MACHINE LEARNING DEMONSTRATION. Journal of Positive School Psychology, 6(2), 169-177.								
106	Jurgiel, J., Miyakoshi, M., Dillon, A., Piacentini, J., & Loo, S. K.	2022	Additive and interactive effects of attention-deficit/hyperactivity disorder and tic disorder on brain connectivity. Biological Psychiatry: Cognitive Neuroscience and Neuroimaging. https://doi.org/10.1016/j.bpsc.2022.10.003								

107	Kato, T., Kaneko, N., Sasaki, A., Endo, N., Yuasa, A., Milosevic, M., Watanabe, K., & Nakazawa, K.	2022	Corticospinal excitability and somatosensory information processing of the lower limb muscle during upper limb voluntary or electrically induced muscle contractions. European Journal of Neuroscience, 55(7), 1810-1824. https://doi.org/10.1111/ejn.15643								
108	Kepler, V. F., Seet, M. S., Hamano, J., Saba, M., Thakor, N. V., Dimitriadis, S. I., & Dragomir, A.	2022	Odor pleasantness modulates functional connectivity in the olfactory hedonic processing network. Brain Sciences, 12(10), 1408. https://doi.org/10.3390/brainsci12101408		0.9						
109	Khoshnoud, S., Alvarez Igarzábal, F., & Wittmann, M.	2022	Brain–Heart Interaction and the Experience of Flow While Playing a Video Game. Frontiers in Human Neuroscience, 16. https://doi.org/10.3389/fnhum.2022.819834	0.8	0.8	0.8			0.8		
110	Klatt, L. I., Getzmann, S., & Schneider, D.	2022	Attentional modulations of alpha power are sensitive to the task-relevance of auditory spatial information. Cortex, 153, 1-20. https://doi.org/10.1016/j.cortex.2022.03.022								0.5
111	Klug, M., Berg, T., & Gramann, K.	2022	No need for extensive artifact rejection for ICA-A multi-study evaluation on stationary and mobile EEG datasets. bioRxiv, 2022-09. https://doi.org/10.1101/2022.09.13.507772								
112	Klug, M., Jeung, S., Wunderlich, A., Gehrke, L., Protzak, J., Djebbara, Z., Argubi-Wollesen, A., Wollesen, B., & Gramann, K.	2022	The BeMoBIL Pipeline for automated analyses of multimodal mobile brain and body imaging data. bioRxiv, 2022-09. https://doi.org/10.1101/2022.09.29.510051								
113	Kmiecik, M. J., Tu, F. F., Darnell, S., Harber, K., & Hellman, K. M.	2022	Early Cortical Mechanisms of Visual Discomfort in Premenarchal Adolescents. Power, 40(30), 20.								
114	Koivisto, M., Jalava, E., Kuusisto, L., Railo, H., & Grassini, S.	2022	Top-down processing and nature connectedness predict psychological and physiological effects of nature. Environment and Behavior, 54(5), 917-945.								
115	Korochkina, M., Nickels, L., & Bürki, A.	2022	An investigation of behavioural and electrophysiological markers of integration in learning of novel names for novel concepts.								
116	Koshiyama, D., Miyakoshi, M., Tanaka-Koshiyama, K., Sprock, J., & Light, G. A.	2022	High-power gamma-related delta phase alteration in schizophrenia patients at rest. Psychiatry and Clinical Neurosciences, 76(5), 179-186. https://doi.org/10.1111/pcn.13331							0.7	
117	Kotowski, K.	2022	ANALIZA SYGNAŁU EEG DLA POTRZEB ROZPOZNAWANIA EMOCJI.								
118	Krugliak, A., & Clarke, A.	2022	Towards real-world neuroscience using mobile EEG and augmented reality. Scientific Reports, 12(1), 1-11. https://doi.org/10.1038/s41598-022-06296-3	0.2	0.2	0.2	0.2	0.2	0.2	0.2	

119	Krugliak, A., & Clarke, A.	2022	Towards real-world neuroscience using mobile EEG and augmented reality.Science report, 12, 2291 https://doi.org/10.1038/s41598-022-06296-3	0.2	0.2	0.2		0.2			
120	Kumaravel, V. P., Buiatti, M., Parise, E., & Farella, E.	2022	Adaptable and Robust EEG Bad Channel Detection Using Local Outlier Factor (LOF). Sensors, 22(19), 7314. https://doi.org/10.3390/s22197314								
121	Kumaravel, V. P., Farella, E., Parise, E., & Buiatti, M.	2022	NEAR: An artifact removal pipeline for human newborn EEG data. Developmental Cognitive Neuroscience Volume 54, April 2022, 101068. https://doi.org/10.1016/j.dcn.2022.101068								
122	Ladouce, S., Mustile, M., Ietswaart, M., & Dehais, F.	2022	Capturing Cognitive Events Embedded in the Real World Using Mobile Electroencephalography and Eye-Tracking. Journal of Cognitive Neuroscience, 34(12), 2237-2255. https://doi.org/10.1162/jocn_a_01903								
123	Langer, N., Plomecka, M. B., Tröndle, M., Negi, A., Popov, T., Milham, M., & Haufe, S.	2022	A benchmark for prediction of psychiatric multimorbidity from resting EEG data in a large pediatric sample. NeuroImage, 258, 119348. https://doi.org/10.1016/j.neuroimage.2022.119348	0.8	0.8	0.8	0.8	0.8			
124	Lanzone, J., Boscarino, M., Tufo, T., Di Lorenzo, G., Ricci, L., Colicchio, G., Lazzaro, V. D., Tombini, M., & Assenza, G.	2022	Vagal nerve stimulation cycles alter EEG connectivity in drug-resistant epileptic patients: A study with graph theory metrics. Clinical Neurophysiology, 142, 59-67. https://doi.org/10.1016/j.clinph.2022.07.503	1.0?	1.0?	1.0?	1.0?	1.0?	1.0?		
125	Larra, M. F., Zhang, X., Finke, J. B., Schächinger, H., Wascher, E., & Arnau, S.	2022	Stress effects on the top-down control of visuospatial attention: Evidence from cue-dependent alpha oscillations. Cognitive, Affective, & Behavioral Neuroscience, 22(4), 722-735. https://doi.org/10.3758/s13415-022-00994-1							0.5	
126	Li, A., Feitelberg, J., Saini, A. P., Höchenberger, R., & Scheltienne, M.	2022	MNE-ICALabel: Automatically annotating ICA components with ICLabel in Python. Journal of Open Source Software, 7(76), 4484. https://doi.org/10.21105/joss.04484								
127	Li, J.	2022	Electrode Evaluation and Electrocortical Dynamics of Adapting to Small Perturbations during Treadmill Walking. https://purls.library.ucf.edu/go/DP0027098								
128	Li, Y., Huang, P., Huang, J., Zhong, Z., Zhou, S., Dong, H., ... & Li, P.	2022	Remote ischemic preconditioning improves cognitive control in healthy adults: Evidence from an event-related potential study. Frontiers in Neuroscience, 16, 936975. https://doi.org/10.3389/fnins.2022.936975								
129	Liang, M.	2022	Electrophysiological Signatures of Spatial and Temporal Coding in Humans (Doctoral dissertation, The University of Arizona).								

130	Liegel, N., Schneider, D., Wascher, E., & Arnau, S.	2022	Task prioritization modulates alpha, theta and beta EEG dynamics reflecting proactive cognitive control. Scientific Reports, 12(1), 15072. https://doi.org/10.1038/s41598-022-19158-9	0.5	0.5	0.5	0.5	0.5			
131	Liegel, N., Schneider, D., Wascher, E., & Arnau, S.	2022	Task prioritization modulates low frequency EEG dynamics reflecting proactive cognitive control. bioRxiv, 2022-05. https://doi.org/10.1101/2022.05.04.490638								
132	Lin, H., Ristic, J., Inzlicht, M., & Otto, A. R.	2022	The average reward rate modulates behavioral and neural indices of effortful control allocation. Journal of Cognitive Neuroscience, 34(11), 2113-2126. https://doi.org/10.1162/jocn_a_01905								
133	Lin, H., Ristic, J., Inzlicht, M., & Otto, A. R.	2022	The opportunity cost of time modulates behavioral and neural indices of effortful control allocation. PsyArXiv. April, 4.	0.95	0.85						0.01
134	Long, C., Hu, X., Qi, G., & Zhang, L.	2022	Self-interest is intuitive during opportunity (in) equity: Evidence from multivariate pattern analysis of electroencephalography data. Neuropsychologia, 174, 108343. https://doi.org/10.1016/j.neuropsychologia.2022.108343								
135	Lopes, F., Leal, A., Medeiros, J., Pinto, M. F., Dourado, A., Dümpelmann, M., & Teixeira, C.	2022	EPIC: Annotated epileptic EEG independent components for artifact reduction. Scientific Data, 9(1), 512. https://doi.org/10.1038/s41597-022-01524-x								
136	Lopes, F., Leal, A., Medeiros, J., Pinto, M. F., Dourado, A., Dümpelmann, M., & Teixeira, C.	2022	Ensemble deep neural network for automatic classification of EEG independent components. IEEE Transactions on Neural Systems and Rehabilitation Engineering, 30, 559-568. https://doi.org/10.1109/TNSRE.2022.3154891								
137	Lu, L., Yang, J., Shu, R., & Long, C.	2022	The default-interventionist model underlies premise typicality weakening the premise diversity effect during category-based induction: Event-related potentials evidence. Scandinavian Journal of Psychology. Volume64, Issue3 June 2023 Pages 325-338 https://doi.org/10.1111/sjop.12892	0.7	0.7	0.7	0.7	0.7			0.05
138	Ma, L.	2022	The Impact of Attentional Focus on Sensory Reweighting for Postural Control in the Aging Adult (Doctoral dissertation, Temple University. Libraries). http://dx.doi.org/10.34944/dspace/8286	0.8	0.8		0.8	0.8			
139	Ma, L., Marshall, P. J., & Wright, W. G.	2022	The impact of external and internal focus of attention on visual dependence and EEG alpha oscillations during postural control. Journal of NeuroEngineering and Rehabilitation, 19(1), 81. https://doi.org/10.1186/s12984-022-01059-7	0.8	0.8		0.8	0.8			

140	Mai, A., Serman, M., Best, S., Jensen, N. S., Foellmer, J., Schroeer, A., ... & Corona-Strauss, F. I.	2022	Speech Tracking in Complex Auditory Scenes with Differentiated In-and Out-Field-Of-View Processing in Hearing Aids. In 2022 44th Annual International Conference of the IEEE Engineering in Medicine & Biology Society (EMBC) (pp. 798-801). IEEE. https://doi.org/10.1109/EMBC48229.2022.9870826								
141	Manolovitz, B.	2022	Effects of Repetition of Unfamiliar Music on Listening Behavior and Neural Responses (Doctoral dissertation, University of Miami).		0.8						
142	Mark, E. B., Liao, D., Nedergaard, R. B., Hansen, T. M., Drewes, A. M., & Brock, C.	2022	Central neuronal transmission in response to tonic cold pain is modulated in people with type 1 diabetes and severe polyneuropathy. Journal of Diabetes and its Complications, 36(8), 108263. https://doi.org/10.1016/j.jdiacomp.2022.108263							0.2	
143	Markovinović, I., Vrankić, M., Vlahinić, S., & Šverko, Z.	2022	Design considerations for the auditory brain computer interface speller. Biomedical Signal Processing and Control, 75, 103546. https://doi.org/10.1016/j.bspc.2022.103546								
144	Marusic, U., Peskar, M., De Pauw, K., Omejc, N., Drevensek, G., Rojc, B., ... & Kavcic, V.	2022	Neural bases of age-related sensorimotor slowing in the upper and lower limbs. Frontiers in aging neuroscience, 355. https://doi.org/10.3389/fnagi.2022.819576		0.85						
145	Menicucci, D., Cesari, V., Cipriani, E., Piarulli, A., & Gemignani, A.	2022	Sleep Deprivation Induces Acute Dissociation via Altered EEG Rhythms Expression and Connectivity. bioRxiv, 2022-03. https://doi.org/10.1101/2022.03.21.485177	0.8	0.8	0.8	0.8	0.8			0.15
146	Menicucci, D., Lunghi, C., Zaccaro, A., Morrone, M. C., & Gemignani, A.	2022	Mutual interaction between visual homeostatic plasticity and sleep in adult humans. Elife, 11, e70633. https://doi.org/10.7554/eLife.70633								
147	Metzen, D., Genç, E., Getzmann, S., Larra, M. F., Wascher, E., & Ocklenburg, S.	2022	Frontal and parietal EEG alpha asymmetry: a large-scale investigation of short-term reliability on distinct EEG systems. Brain Structure and Function volume 227, pages725–740 (2022). https://doi.org/10.1007/s00429-021-02399-1		0.8						
148	Mijancos Martínez, G.	2022	Improving the quality of combined EEG-TMS neural recordings: artifact removal and time analysis (Master's thesis, Universitat Politècnica de Catalunya). http://hdl.handle.net/2117/369068								
149	Mishra, S., Srinivasan, N., & Tiwary, U. S.	2022	Dynamic functional connectivity of emotion processing in beta band with naturalistic emotion stimuli. Brain sciences, 12(8), 1106. https://doi.org/10.3390/brainsci12081106	1.0?	1.0?	1.0?	1.0?	1.0?	1.0?		
150	Mishra, S., Srinivasan, N., & Tiwary, U. S.	2022	Cardiac–brain dynamics depend on context familiarity and their interaction predicts experience of emotional arousal.								

			Brain Sciences, 12(6), 702. https://doi.org/10.3390/brainsci12060702								
151	Miyamoto, K., Tanaka, H., & Nakamura, S.	2022	Online EEG-based emotion prediction and music generation for inducing affective states. IEICE TRANSACTIONS on Information and Systems, 105(5), 1050-1063. https://doi.org/10.1587/transinf.2021EDP7171								
152	Monachino, A. D., Lopez, K. L., Pierce, L. J., & Gabard-Durnam, L. J.	2022	The HAPPE plus Event-Related (HAPPE+ ER) software: A standardized preprocessing pipeline for event-related potential analyses. Developmental Cognitive Neuroscience, 57, 101140. https://doi.org/10.1016/j.dcn.2022.101140	0.8?	0.8?	0.8?	0.8?	0.8?	0.8?		
153	Morgan-Short, K., Finestrat, I., Luque, A., & Abugaber, D.	2022	Exploring New Insights Into Explicit and Implicit Second Language Processing: Event-Related Potentials Analyzed by Source Attribution. Language Learning, 72(2), 365-411. https://doi.org/10.1111/lang.12492								
154	Mota, N. B., Soares, E., Altszyler, E., Sánchez-Gendriz, I., Muto, V., Heib, D., Slezak, D. F., Sigman, M., Copelli, M., Schabus, M., & Ribeiro, S.	2022	Imagetic and affective measures of memory reverberation diverge at sleep onset in association with theta rhythm. NeuroImage, 264, 119690. https://doi.org/10.1016/j.neuroimage.2022.119690	1.0?	1.0?	1.0?	1.0?	1.0?	1.0?		
155	Movahed, R. A., & Rezaeian, M.	2022	Automatic Diagnosis of Mild Cognitive Impairment Based on Spectral, Functional Connectivity, and Nonlinear EEG-Based Features. Computational and Mathematical Methods in Medicine, 2022. https://doi.org/10.1155/2022/2014001								
156	Movahed, R. A., Jahromi, G. P., Shahyad, S., & Meftahi, G. H.	2022	A major depressive disorder diagnosis approach based on EEG signals using dictionary learning and functional connectivity features. Physical and Engineering Sciences in Medicine, 45(3), 705-719. https://doi.org/10.1007/s13246-022-01135-1								
157	Mustile, M., Kourtis, D., Edwards, M. G., Donaldson, D. I., & Ietswaart, M.	2022	The neural response is heightened when watching a person approaching compared to walking away: Evidence for dynamic social neuroscience. Neuropsychologia, 175, 108352. https://doi.org/10.1016/j.neuropsychologia.2022.108352								
158	Mygind, L., Clark, G. M., Bigelow, F. J., Fuller-Tyszkiewicz, M., Knibbs, L. D., Mavoa, S., ... & Enticott, P. G.	2022	Green matter: is there an association between vegetation cover around the home and social brain function in childhood?.								

159	Nárai, Á., Nemecz, Z., Vidnyánszky, Z., & Weiss, B.	2022	Lateralization of orthographic processing in fixed-gaze and natural reading conditions. Cortex, 157, 99-116. https://doi.org/10.1016/j.cortex.2022.07.017								
160	Nguyen, T. N. Q., Vo, H. T. T., Nguyen, H. A., & Van Huynh, T.	2022	Machine Learning in Classification of Parkinson's Disease Using Electroencephalogram with Simon's Conflict. In Computational Intelligence Methods for Green Technology and Sustainable Development: Proceedings of the International Conference GTSD2022 (pp. 110-122). Cham: Springer International Publishing. https://doi.org/10.1007/978-3-031-19694-2_10								
161	Nikolaev, A. R., Bramão, I., Johansson, R., & Johansson, M.	2022	Episodic memory formation in naturalistic viewing. bioRxiv, 2022-05. https://doi.org/10.1101/2022.05.24.485821	0.4	0.9	0.05	0.4	0.4	0.4		
162	Nilsson, E. J., Bärgman, J., Ljung Aust, M., Matthews, G., & Svanberg, B.	2022	Let complexity bring clarity: a multidimensional assessment of cognitive load using physiological measures. Frontiers in neuroergonomics, 3, 2. https://doi.org/10.3389/fnrgo.2022.787295		0.7						0.2
163	Niu, H., Zhai, Y., Huang, Y., Wang, X., & Wang, X.	2022	Investigating the short-term cognitive abilities under local strong thermal radiation through EEG measurement. Building and Environment Volume 224, October 2022, 109567. https://doi.org/10.1016/j.buildenv.2022.109567								
164	Oh, J.	2022	Assessment of human finger position sense and the effect of vibro-tactile stimulation on proprioceptive acuity. https://hdl.handle.net/11299/243131.								
165	Oh, Y.	2022	Classification of Insight and Analytic Problem-Solving Using an Interpretable Deep-Learning Model Based on Single-Trial Eeg Data. Drexel University.								
166	Oliaee, A., Mohebbi, M., Shirani, S., & Rostami, R.	2022	Extraction of discriminative features from EEG signals of dyslexic children; before and after the treatment. Cognitive Neurodynamics, 16(6), 1249-1259. https://doi.org/10.1007/s11571-022-09794-2								
167	Osorio, S., Straube, B., Meyer, L., & He, Y.	2022	Co-speech gestures enhance lexical-semantic prediction: Naturalistic evidence from time-resolved regression ERPs.								
168	Ossandon, J. P., Roder, B., & Stange, L.	2022	Crossmodal visual predictions elicit spatially specific early visual cortex activity but later than real visual stimuli. bioRxiv, 2022-12. https://doi.org/10.1101/2022.12.14.520404	0.8	0.8	0.8	0.8				
169	Ouyang, G., Dien, J., & Lorenz, R.	2022	Handling EEG artifacts and searching individually optimal experimental parameter in real time: a system development and demonstration. Journal of Neural Engineering, 19(1), 016016. https://doi.org/10.1088/1741-2552/ac42b6								

170	Park, J., Jang, S., Gwak, J., Kim, B. C., Lee, J. J., Choi, K. Y., ... & Ahn, S.	2022	Individualized diagnosis of preclinical Alzheimer's Disease using deep neural networks. Expert Systems with Applications, 210, 118511. https://doi.org/10.1016/j.eswa.2022.118511								
171	Parmigiani, S., Ross, J. M., Cline, C., Minasi, C. B., Gogulski, J., & Keller, C. J.	2022	Reliability and validity of TMS-EEG biomarkers. arXiv preprint arXiv:2207.08456. https://doi.org/10.48550/arXiv.2207.08456								
172	Peh, W. Y., Yao, Y., & Dauwels, J.	2022	Transformer convolutional neural networks for automated artifact detection in scalp EEG. In 2022 44th Annual International Conference of the IEEE Engineering in Medicine & Biology Society (EMBC) (pp. 3599-3602). IEEE. https://doi.org/10.1109/EMBC48229.2022.9871916								
173	Penalver-Andres, J. A., Buetler, K. A., Koenig, T., Müri, R. M., & Marchal-Crespo, L.	2022	Resting-State Functional Networks Correlate with Motor Performance in a Complex Visuomotor Task: An EEG Microstate Pilot Study on Healthy Individuals. Brain topography, 1-18. https://doi.org/10.1007/s10548-022-00934-9								0.4
174	Pentz, A., Timpe, C. M. M., Normann, E. M., Slapø, N. B., Melle, I., Lagerberg, T. V., ... & Elvsåshagen, T.	2022	Mismatch Negativity in Schizophrenia Spectrum and Bipolar Disorders: Group and Sex Differences and Associations with Symptom Severity. https://ssrn.com/abstract=4105403								
175	Perera, M. P. N., Mallawaarachchi, S., Bailey, N. W., Murphy, O. W., & Fitzgerald, P. B.	2022	Obsessive-Compulsive Disorder (OCD) is Associated with Increased Engagement of Frontal Brain Regions Across Multiple Event Related Potentials. bioRxiv, 2022-11. https://doi.org/10.1101/2022.11.05.515279								
176	Perez, T. M., Glue, P., Adhia, D. B., Navid, M. S., Zeng, J., Dillingham, P., Smith, M., Niazi, I. K., Young, C. K., & De Ridder, D.	2022	Infraslow closed-loop brain training for anxiety and depression (ISAD): a protocol for a randomized, double-blind, sham-controlled pilot trial in adult females with internalizing disorders. Trials, 23(1), 1-26. https://doi.org/10.1186/s13063-022-06863-z								
177	Persici, V., Blain, S. D., Iversen, J. R., Key, A. P., Kotz, S. A., McAuley, J. D., & Gordon, R. L.	2022	Individual differences in neural markers of beat processing predict spoken grammar skills in six-year-old children.								
178	Pokorny, V. J., Sponheim, S. R., & Rawls, E.	2022	Impact of reduced-dimensionality independent components analysis on event-related potential measurements. Psychophysiology, e14223. https://doi.org/10.1111/psyp.14223	0.8	0.8	0.8	0.8	0.8			
179	Polver, S., Quadrelli, E., Turati, C., & Bulf, H.	2022	Decoding functional brain networks through graph measures in infancy: The case of emotional faces.								

			Biological Psychology, 170, 108292. https://doi.org/10.1016/j.biopsycho.2022.108292								
180	Rahman, M. L., Files, B. T., Oiknine, A. H., Pollard, K. A., Khooshabeh, P., Song, C., & Passaro, A. D.	2022	Combining Neural and Behavioral Measures Enhances Adaptive Training. Frontiers in Human Neuroscience, 16, 18. https://doi.org/10.3389/fnhum.2022.787576	0.7	0.7	0.7	0.7	0.7	0.7		
181	Railo, H., Varjonen, A., Lehtonen, M., & Sikka, P.	2022	Event-related potential correlates of learning to produce novel foreign phonemes. Neurobiology of Language, 3(4), 599-614. https://doi.org/10.1162/nol_a_00080							0.7	
182	Railo, H., Varjonen, A., Lehtonen, M., & Sikka, P.	2022	Changes in late frontal event-related potentials to self-produced foreign phonemes correlate with improvements in pronunciation. https://doi.org/10.1101/2022.01.18.476741							0.7	
183	Ramírez-Mendoza, R. A., Lozoya-Santos, J. D. J., Zavala-Yoé, R., Alonso-Valerdi, L. M., Morales-Menendez, R., Carrión, B., Ponce, Cruz, P. P., & Gonzalez-Hernandez, H. G. (Eds.).	2022	Biometry: Technology, Trends and Applications. CRC Press.								
184	Reed, C. L., Siqi-Liu, A., Lydic, K., Lodge, M., Chitre, A., Denaro, C., Petropoulos, A., Joshi, J., Bukach, C. M., & Couperus, J. W.	2022	Selective contributions of executive function ability to the P3. International Journal of Psychophysiology, 176, 54-61. https://doi.org/10.1016/j.ijpsycho.2022.03.004								
185	Richardson, D. P., Foxe, J. J., Mazurek, K. A., Abraham, N., & Freedman, E. G.	2022	Neural markers of proactive and reactive cognitive control are altered during walking: a mobile brain-body imaging (MoBI) study. NeuroImage, 247, 118853. https://doi.org/10.1016/j.neuroimage.2021.118853								0.7
186	Rodrigues, J., Ziebell, P., Müller, M., & Hewig, J.	2022	Standardizing continuous data classifications in a virtual T-maze using two-layer feedforward networks. Scientific Reports, 12(1), 12879. https://doi.org/10.1038/s41598-022-17013-5								
187	Rodriguez-Larios, J., ElShafei, A., Wiehe, M., & Haegens, S.	2022	Visual working memory recruits two functionally distinct alpha rhythms in posterior cortex. bioRxiv, 2022-04. https://doi.org/10.1101/2022.04.15.488484	0.8	0.8	0.8		0.8			
188	Russo, V., Bilucaglia, M., Circi, R., Bellati, M., Valesi, R., Laureanti, R., Licitra, G., & Zito, M.	2022	The Role of the Emotional Sequence in the Communication of the Territorial Cheeses: A Neuromarketing Approach. Foods, 11(15), 2349. https://doi.org/10.3390/foods11152349								0.7

189	Ryan, D. B., Eckert, M. A., Sellers, E. W., Schairer, K. S., McBee, M. T., Jones, M. R., & Smith, S. L.	2022	Impact of Effortful Word Recognition on Supportive Neural Systems Measured by Alpha and Theta Power. Ear and Hearing, 43(5), 1549-1562. https://doi.org/10.1097/AUD.0000000000001211								
190	Rygvold, T. W., Hatlestad-Hall, C., Elvsåshagen, T., Moberget, T., & Andersson, S.	2022	Long term potentiation-like neural plasticity and performance-based memory function. Neurobiology of Learning and Memory, 196, 107696. https://doi.org/10.1016/j.nlm.2022.107696								
191	Rygvold, T. W., Hatlestad-Hall, C., Elvsåshagen, T., Moberget, T., & Andersson, S.	2022	Long-Term Potentiation-Like Visual Synaptic Plasticity Is Negatively Associated With Self-Reported Symptoms of Depression and Stress in Healthy Adults. Frontiers in Human Neuroscience, 16. https://doi.org/10.3389/fnhum.2022.867675								
192	Sabu, P., Stuldreher, I. V., Kaneko, D., & Brouwer, A. M.	2022	A review on the role of affective stimuli in event-related frontal alpha asymmetry. Frontiers in Computer Science, 4(4). https://doi.org/10.3389/fcomp.2022.869123								
193	Salous, M., Küster, D., Scheck, K., Dikfidan, A., Neumann, T., Putze, F., & Schultz, T.	2022	SmartHelm: User Studies from Lab to Field for Attention Modeling. In 2022 IEEE International Conference on Systems, Man, and Cybernetics (SMC) (pp. 1012-1019). IEEE. https://doi.org/10.1109/SMC53654.2022.9945155	1.0?	1.0?	1.0?	1.0?	1.0?	1.0?		
194	Samiei, S., Delrobaei, M., & Khadem, A.	2022	Evaluating the Effect of Increasing Working Memory Load on EEG-Based Functional Brain Networks. Frontiers in Biomedical Technologies, 9(3), 160-169. https://doi.org/10.18502/fbt.v9i3.9641								
195	Sandberg, C. W., Exton, E., Coburn, K. L., Chun, S., & Miller, C.	2022	Event related potential exploration of the organizational structure of abstract versus concrete words in neurologically intact younger adults. Brain and Language, 230, 105138. https://doi.org/10.1016/j.bandl.2022.105138								
196	Sarailoo, R., Latifzadeh, K., Amiri, S. H., Bossaghzadeh, A., & Ebrahimpour, R.	2022	Assessment of Instantaneous Cognitive Load Imposed by Educational Multimedia using EEG Signals. Frontiers in Neuroscience, 1275. https://doi.org/10.3389/fnins.2022.744737	0.9?	0.9?	0.9?	0.9?	0.9?	0.9?		
197	Scanlon, J. E. M., Jacobsen, N. S. J., Maak, M. C., & Debener, S.	2022	Stepping in time: Alpha-mu and beta oscillations during a walking synchronization task. NeuroImage Volume 253, June 2022, 119099. https://doi.org/10.1016/j.neuroimage.2022.119099	0.7	0.7	0.7	0.7	0.7	0.7		
198	Schneider, J.	2022	Manual or Automatic? Putting standardized EEG-Preprocessing to the Test with the Alpha-Asymmetries of Personality.	0.8	0.8	0.8	0.8	0.8			
199	Schubert, A. L., Löffler, C., & Hagemann, D.	2022	A neurocognitive psychometrics account of individual differences in attentional control. Journal of Experimental								

			Psychology: General, 151(9), 2060. https://doi.org/10.1037/xge0001184								
200	Scilingo, E. P., & Greco, A.	2022	Human body odors of happiness and fear modulate the late positive potential component during neutral face processing: a preliminary ERP study on healthy subjects.	1.0?	1.0?	1.0?	1.0?	1.0?	1.0?		
201	Serban, C. A., Barborica, A., Roceanu, A. M., Mindruta, I., Ciurea, J., Pâslaru, A. C., ... & Moldovan, M.	2022	A method to assess the default EEG macrostate and its reactivity to stimulation. Clinical Neurophysiology, 134, 50-64. https://doi.org/10.1016/j.clinph.2021.12.002								
202	Sessa, P., Lomoriello, A. S., Duma, G. M., Mento, G., Stefani, E. D., & Ferrari, P. F.	2022	Degenerate pathway for processing smile and other emotional expressions in congenital facial palsy: an hdEEG investigation. Article Information DOI: https://doi.org/10.1098/rstb.2021.0190 PubMed:36126673 Published by:Royal Society Print ISSN:0962-8436 Online ISSN:1471-2970	0.7	0.7	0.7	0.7	0.7	0.7		
203	Seymour, R. A., Alexander, N., Mellor, S., O'Neill, G. C., Tierney. T. M., Barnes, G. R., & Maguire, E. A.	2022	Interference suppression techniques for OPM-based MEG: Opportunities and challenges. NeuroImage Volume 247, 15 February 2022, 118834. https://doi.org/10.1016/j.neuroimage.2021.118834								
204	Sherman, D. A., Baumeister, J. O. C. H. E. N., Stock, M. S., Murray, A. M., Bazett-Jones, D. M., & Norte, G. E.	2022	Inhibition of motor planning and response selection following ACL reconstruction. Medicine & Science in Sports & Exercise, 55(3), 440-449. https://doi.org/10.1249/MSS.0000000000003072								
205	Sherman, D. A., Baumeister, J., Stock, M. S., Murray, A. M., Bazett-Jones, D. M., & Norte, G. E.	2022	Weaker Quadriceps Corticomuscular Coherence in Individuals Following ACL Reconstruction during Force Tracing. Medicine and Science in Sports and Exercise. https://doi.org/10.1249/mss.0000000000003080								
206	Sherman, D. A., Lehmann, T., Baumeister, J., Grooms, D. R., & Norte, G. E.	2022	Somatosensory perturbations influence cortical activity associated with single-limb balance performance. Experimental Brain Research, 240(2), 407-420. https://doi.org/10.1007/s00221-021-06260-z								
207	Shetty, A., Hebbar, S. P., Shenoy, R., Peter, V., & Krishnan, G.	2022	A prime-masked ERP investigation on phonology in visual word processing among bilingual speakers of alphasyllabic and alphabetic orthographies. Scientific Reports, 12(1), 1-10. https://doi.org/10.1038/s41598-022-13654-8	0.8	0.8			0.8			

208	Shirani, S., & Mohebbi, M.	2022	Brain functional connectivity analysis in patients with relapsing-remitting multiple sclerosis: A graph theory approach of EEG resting state. Frontiers in Neuroscience, 16. https://doi.org/10.3389/fnins.2022.801774	1.0?	1.0?	1.0?	1.0?	1.0?	1.0?		
209	Simon, A., Østergaard, J., Bech, S., & Loquet, G.	2022	Optimal time lags for linear cortical auditory attention. https://doi.org/10.5281/zenodo.6576990								
210	Somon, B., Giebeler, Y., Darmet, L., & Dehais, F.	2022	Benchmarking cEEGrid and solid gel-based electrodes to classify inattentional deafness in a flight simulator. Frontiers in Neuroergonomics, 2, 802486. https://doi.org/10.3389/fnrgo.2021.802486	0.7	0.7	0.7	0.7	0.7	0.7		
211	Song, T., Xu, L., Peng, Z., Wang, L., Dai, C., Xu, M., Shao, Y., & Li, S.	2022	Total sleep deprivation impairs visual selective attention and triggers a compensatory effect: evidence from event-related potentials. Cognitive Neurodynamics, 1-11. https://doi.org/10.1007/s11571-022-09861-8								
212	Souza, R. F. L. D., Mendes, T. M. A. S., Lima, L. A. B. D. A., Brandão, D. S., Laplagne, D. A., & Sousa, M. B. C. D.	2022	Effect of the menstrual cycle on electroencephalogram alpha and beta bands during motor imagery and action observation. Frontiers in Human Neuroscience, 16, 276. https://doi.org/10.3389/fnhum.2022.878887	0.8	0.8			0.8			
213	Stergiadis, C., Kostaridou, V. D., Veloudis, S., Kazis, D., & Klados, M. A.	2022	A Personalized User Authentication System Based on EEG Signals. Sensors, 22(18), 6929. https://doi.org/10.3390/s22186929								
214	Strang, C. C., Harris, A., Moody, E. J., & Reed, C. L.	2022	Peak frequency of the sensorimotor mu rhythm varies with autism-spectrum traits. Frontiers in Neuroscience, 16, 950539. https://doi.org/10.3389/fnins.2022.950539								
215	Studnicki, A., Downey, R. J., & Ferris, D. P.	2022	Characterizing and Removing Artifacts Using Dual-Layer EEG during Table Tennis. Sensors 2022, 22(15), 5867; https://doi.org/10.3390/s22155867								
216	Suarez, J. X., Gramann, K., Ochoa, J. F., Toro, J. P., Mejia, A. M., & Hernandez, A. M.	2022	Changes in brain activity of trainees during laparoscopic surgical virtual training assessed with electroencephalography. Brain Research, 1783, 147836. https://doi.org/10.1016/j.brainres.2022.147836								
217	Susam, B. T., Riek, N. T., Beck, K., Eldeeb, S., Hudac, C. M., Gable, P. A., Conner, C., Akcakaya, M., White, S., & Mazefsky, C.	2022	Quantitative EEG Changes in Youth With ASD Following Brief Mindfulness Meditation Exercise. IEEE Transactions on Neural Systems and Rehabilitation Engineering, 30, 2395-2405. https://doi.org/10.1109/TNSRE.2022.3199151								
218	Suviseshamuthu, E. S., Shenoy Handiru, V., Allexandre, D., Hoxha,	2022	EEG-Based Spectral Analysis Showing Brainwave Changes Related to Modulating Progressive Fatigue During a Prolonged Intermittent Motor Task. Frontiers in	0.2	0.2	0.2	0.2	0.2			

	A., Saleh, S., & Yue, G. H.		Human Neuroscience, 16, 100. https://doi.org/10.3389/fnhum.2022.770053								
219	Suzuki, K., Sugaya, M., & Laohakangvalvit, T.	2022	Preliminary Study on Model Construction for Estimating Mental Illness and Developmental Disorders. In 2022 IEEE 4th Global Conference on Life Sciences and Technologies (LifeTech) (pp. 327-330). IEEE. https://doi.org/10.1109/LifeTech53646.2022.9754853								
220	Tang, Y., Tang, R., Wang, X., Yu, L., Zhang, Y., Wang, S., Cao, F., & Ding, X.	2022	Efficiency moderates the relationship between sleep-onset insomnia and resting-state electroencephalogram microstate. Journal of integrative neuroscience, 21(2), 52. https://doi.org/10.31083/j.jin2102052								
221	Telesford, Q. K., Gonzalez-Moreira, E., Xu, T., Tian, Y., Colcombe, S., Cloud, J., Russ, B. E., Falchier, A., Nentwich, N., Madsen, J., Parra, L., Schroeder, C., Milham, M., & Franco, A. R.	2022	An open-access dataset of naturalistic viewing using simultaneous EEG-fMRI. bioRxiv, 2022-11. https://doi.org/10.1101/2022.11.23.517540								
222	Terpou, B. A., Shaw, S. B., Théberge, J., Férat, V., Michel, C. M., McKinnon, M. C., Lanius, R. A., & Ros, T.	2022	Spectral decomposition of EEG microstates in post-traumatic stress disorder. NeuroImage: Clinical, 35, 103135. https://doi.org/10.1016/j.nicl.2022.103135	0.5							
223	Tian, Q., Yang, N. B., Fan, Y., Dong, F., Bo, Q. J., Zhou, F. C., ... & Fan, M.	2022	Detection of schizophrenia cases from healthy controls with combination of neurocognitive and electrophysiological features. Frontiers in Psychiatry, 13, 810362. https:\\doi.org/10.3389/fpsyt.2022.810362								
224	Tichko, P., Page, N., Kim, J. C., Large, E. W., & Loui, P.	2022	Neural Entrainment to Musical Pulse in Naturalistic Music Is Preserved in Aging: Implications for Music-Based Interventions. Brain Sciences, 12(12), 1676. https://doi.org/10.3390/brainsci12121676	0.9	0.9						
225	Tröndle, M., Popov, T., Dziemian, S., & Langer, N.	2022	Decomposing the role of alpha oscillations during brain maturation. Elife, 11, e77571. https://doi.org/10.7554/eLife.77571	0.8	0.8	0.8	0.8	0.8			
226	Tsai, B. Y., Diddi, S. V. S., Ko, L-W., Wang, S-J., Chang, C-Y., & Jung, T-P.	2022	Development of an Adaptive Artifact Subspace Reconstruction Based on Hebbian/Anti-Hebbian Learning Networks for Enhancing BCI Performance								
227	Upshaw, J. D., Stevens Jr, C. E., Ganis, G., & Zabelina, D. L.	2022	The hidden cost of a smartphone: The effects of smartphone notifications on cognitive control from a behavioral and electrophysiological perspective. Plos one,	0.945	0.945	0.945	0.945	0.945	0.945		

			17(11), e0277220. https://doi.org/10.1371/journal.pone.0277220								
228	van Boxtel, W. S.	2022	A Matter of Memory? Sentence Comprehension in Healthy Aging (Doctoral dissertation, University of Essex).								
229	Van Hoornweder, S., Blanco-Mora, D. A., Depestele, S., van Dun, K., Cuypers, K., Verstraelen, S., & Meesen, R.	2022	Aging and Complexity Effects on Hemisphere-Dependent Movement-Related Beta Desynchronization during Bimanual Motor Planning and Execution. Brain Sciences, 12(11), 1444. https://doi.org/10.3390/brainsci12111444	0.5	0.5	0.7	0.7	0.7	0.7		
230	van Noordt, S., Desjardins, J. A., BASIS Team, & Elsabbagh, M.	2022	Inter-trial theta phase consistency during face processing in infants is associated with later emerging autism. Autism Research, 15(5), 834-846. https://doi.org/10.1002/aur.2701								
231	Visser, A., Büchel, D., Lehmann, T., & Baumeister, J.	2022	Continuous table tennis is associated with processing in frontal brain areas: an EEG approach. Experimental Brain Research, 240(6), 1899-1909. https://doi.org/10.1007/s00221-022-06366-y							0.9	
232	Vitório, R., Morris, R., Das, J., Walker, R., Mancini, M., & Stuart, S.	2022	Brain activity response to cues during gait in Parkinson's disease: A study protocol. Plos one, 17(11), e0275894. https://doi.org/10.1371/journal.pone.0275894								
233	Vourvopoulos, A., Blanco-Mora, D. A., Aldridge, A., Jorge, C., Figueiredo, P., & i Badia, S. B.	2022	Enhancing Motor-Imagery Brain-Computer Interface Training With Embodied Virtual Reality: A Pilot Study With Older Adults. In 2022 IEEE International Conference on Metrology for Extended Reality, Artificial Intelligence and Neural Engineering (MetroXRAINE) (pp. 157-162). IEEE. https://doi.org/10.1109/MetroXRAINE54828.2022.9967664								
234	Vourvopoulos, A., Blanco-Mora, D., Aldridge, A., Jorge, C., Fernandes, J. C., Figueiredo, P., & i Badia, S. B.	2022	Influence of VR-based Brain-Computer Interfaces Training in Brain Activity and Clinical Outcome in Chronic Stroke: A Longitudinal Study of Single Cases. https://doi.org/10.21203/rs.3.rs-2193322/v1	0.9	0.9						
235	Wadsley, C. G., Cirillo, J., Nieuwenhuys, A., & Byblow, W. D.	2022	Decoupling countermands nonselective response inhibition during selective stopping. Journal of Neurophysiology, 127(1), 188-203. https://doi.org/10.1152/jn.00495.2021								
236	Wakim, K. M., Foxe, J., & Molholm, S.	2022	Sensorimotor processing in autism and typical development: a high-density electrical mapping study of response-locked neural activity in children and adolescents. Authorea Preprints. https://doi.org/10.22541/au.166853521.16135258/v1		0.8						0.05

237	Wang, B.	2022	Endophenotypes for Psychosis: Genetic Basis and Mechanistic Insights (Doctoral dissertation, UCL (University College London)).		0.8						
238	Wang, G., Yang, Y., Wang, J., Hao, Z., Luo, X., & Liu, J.	2022	Dynamic changes of brain networks during standing balance control under visual conflict. Frontiers in neuroscience, 16, 1003996. https://doi.org/10.3389/fnins.2022.1003996								
239	Wang, L., Wang, Y., Liu, Z., Wu, E. X., & Chen, F.	2022	A Speech-Level–Based Segmented Model to Decode the Dynamic Auditory Attention States in the Competing Speaker Scenes. Frontiers in Neuroscience, 15. https://doi.org/10.3389/fnins.2021.760611								
240	Wang, P., Pathania, A., Euler, M. J., Duff, K., & Schaefer, S. Y.	2022	Investigating the relationship between resting-state EEG frontoparietal coherence, visuospatial ability, and motor skill acquisition: A retrospective analysis. NeuroRegulation, 9(2), 82-82. https://doi.org/10.15540/nr.9.2.82	0.9	0.9						
241	Wang, R., Yu, R., Tian, Y., & Wu, H.	2022	Individual variation in the neurophysiological representation of negative emotions in virtual reality is shaped by sociability. NeuroImage Volume 263, November 2022, 119596. https://doi.org/10.1016/j.neuroimage.2022.119596	0.7	0.7						
242	Wang, W., & Li, B.	2022	A novel model based on a 1D-ResCNN and transfer learning for processing EEG attenuation. Computer Methods in Biomechanics and Biomedical Engineering, 1-14. https://doi.org/10.1080/10255842.2022.2162339								
243	Wang, W., Li, B., & Wang, H.	2022	A novel end-to-end network based on a bidirectional GRU and a self-attention mechanism for denoising of electroencephalography signals. Neuroscience, 505, 10-20. https://doi.org/10.1016/j.neuroscience.2022.10.006								
244	Wascher, E., Arnau, S., Gutberlet, M., Chuang, L. L., Rinkenauer, G., & Reiser, J. E.	2022	Visual Demands of Walking Are Reflected in Eye-Blink-Evoked EEG-Activity. Applied Sciences, 12(13), 6614. https://doi.org/10.3390/app12136614		0.3						
245	Wascher, E., Sharifian, F., Gutberlet, M., Schneider, D., Getzmann, S., & Arnau, S.	2022	Mental chronometry in big noisy data. PloS one, 17(6), e0268916. https://doi.org/10.1371/journal.pone.0268916		0.3						0.3
246	Weiblen, R., Robert, C., Petereit, P., Heldmann, M., Munte, T. F., Munchau, A., ... & Kramer, U. M.	2022	Neural, physiological and behavioral correlates of empathy for pain in Tourette syndrome. bioRxiv, 2022-12. https://doi.org/10.1101/2022.12.19.521056								

247	Weiss, B., Nárai, Á., & Vidnyánszky, Z.	2022	Lateralization of early orthographic processing during natural reading is impaired in developmental dyslexia. NeuroImage Volume 258, September 2022, 119383. https://doi.org/10.1016/j.neuroimage.2022.119383								
248	Widmann, A., & Schröger, E.	2022	Intention-based predictive information modulates auditory deviance processing. Frontiers in Neuroscience, 16. https://doi.org/10.3389/fnins.2022.995119		1						
249	Wikström, V., Saarikivi, K., Falcon, M., Makkonen, T., Martikainen, S., Putkinen, V., Cowley, B. U., & Tervaniemi, M.	2022	Inter-brain synchronization occurs without physical co-presence during cooperative online gaming. Neuropsychologia, 174, 108316. https://doi.org/10.1016/j.neuropsychologia.2022.108316		0.9						
250	Wiwatowska, E., Czajeczny, D., & Michałowski, J. M.	2022	Decreased preparatory activation and inattention to cues suggest lower activation of proactive cognitive control among high procrastinating students. Cognitive, Affective, & Behavioral Neuroscience, 171-186. https://doi.org/10.3758/s13415-021-00945-2								
251	Wiwatowska, E., Pietruch, M., Katafoni, P., & Michałowski, J. M	2022	“I can’t focus now, I will study tomorrow”-The link between academic procrastination and resistance to distraction.								
252	Wiwatowska, E., Wypych, M., & Michałowski, J. M.	2022	The Influence of Norm-referenced Feedback on Attention and Performance Monitoring Among High and Low Procrastinating Students.								
253	Wolpaw, J., Ozsoy, S., Berenholtz, S., Wright, S., Bowen, K., Gogula, S., ... & Toy, S.	2022	A Multimodal Evaluation of Podcast Learning, Retention, and Electroencephalographically Measured Attention in Medical Trainees. Cureus, 14(11). https://doi.org/10.7759/cureus.31289								
254	Wu, M., Luo, B., Yu, Y., Li, X., Gao, J., Li, J., Sorger, B., & Riecke, L.	2022	Rhythmic musical-electrical trigeminal nerve stimulation improves impaired consciousness. NeuroImage: Clinical, 36, 103170. https://doi.org/10.1016/j.nicl.2022.103170								
255	Yang, B., Ma, J., Ding, R., Xia, X., & Ding, X.	2022	The role of task demands in racial face encoding. Scientific Reports, 12(1), 18896. https://doi.org/10.1038/s41598-022-19880-4								
256	Yang, Y., Truong, N. D., Maher, C., Nikpour, A., & Kavehei, O.	2022	Continental generalization of a human-in-the-loop AI system for clinical seizure recognition. Expert Systems with Applications, 207, 118083.								
257	Yaroshchak, S., & Smaida, M.	2022	ISSN 2522-9400 European Modern Studies Journal Vol 5 No 6.								
258	Yeh, T. C., Huang, C. C. Y., Chung, Y. A., Im, J. J., Lin, Y. Y., Ma, C. C.,	2022	High-frequency transcranial random noise stimulation modulates gamma-band EEG source-based large-scale functional network connectivity in patients with schizophrenia: A randomized, double-blind, sham-								

	Tzeng, N-S., & Chang, H. A.		controlled clinical trial. Journal of Personalized Medicine, 12(10), 1617. https://doi.org/10.3390/jpm12101617								
259	Yi, Y., Billor, N., Liang, M., Cao, X., Ekstrom, A., & Zheng, J.	2022	Classification of EEG signals: an interpretable approach using functional data analysis. Journal of Neuroscience Methods, 376, 109609. https://doi.org/10.1016/j.jneumeth.2022.109609	0.9	0.9	0.9	0.9	0.9			
260	Yu, J., Li, C., Lou, K., Wei, C., & Liu, Q.	2022	Embedding decomposition for artifacts removal in EEG signals. Journal of Neural Engineering, 19(2), 026052. https://doi.org/10.1088/1741-2552/ac63eb								
261	Zhang, Z., Yu, X., Rong, X., & Iwata, M.	2022	A Novel Multimodule Neural Network for EEG Denoising. IEEE Access, 10, 49528-49541. https://doi.org/10.1109/ACCESS.2022.3173261								
262	Zhou, D., Zhang, G., Dang, J., Unoki, M., & Liu, X.	2022	Detection of brain network communities during natural speech comprehension from functionally aligned EEG sources. Frontiers in Computational Neuroscience, 16. https://doi.org/10.3389/fncom.2022.919215								
263	Zhou, Y., Xu, Z., Niu, Y., Wang, P., Wen, X., Wu, X., & Zhang, D.	2022	Cross-Task cognitive workload recognition based on EEG and domain adaptation. IEEE Transactions on Neural Systems and Rehabilitation Engineering, 30, 50-60. https://doi.org/10.1109/TNSRE.2022.3140456								
264	Zhu, B., Cruz-Garza, J. G., Yang, Q., Shoaran, M., & Kalantari, S.	2022	Identifying uncertainty states during wayfinding in indoor environments: An EEG classification study. Advanced Engineering Informatics, 54, 101718. https://doi.org/10.1016/j.aei.2022.101718								
265	Zhu, F., Li, Y., Shi, Z., & Shi, W.	2022	TV-NARX and Coiflets WPT based time-frequency Granger causality with application to corticomuscular coupling in hand-grasping. Frontiers in Neuroscience, 16, 1014495. https://doi.org/10.3389/fnins.2022.1014495								
266	Zhu, X., Rong, W., Zhao, L., He, Z., Yang, Q., Sun, J., & Liu, G.	2022	EEG emotion classification network based on attention fusion of multi-channel band features. Sensors, 22(14), 5252. https://doi.org/10.3390/s22145252								

2023

Index	Author	Year	Journal information	muscle	eye	heart	line noise	channel noise	other	brain	brain removed
1	Jeon, J., & Cai, H.	2023	Wearable EEG-based construction hazard identification in virtual and real environments: A comparative study. Safety Science, 165, 106213.								
2	Rodriguez-Larios, J., & Haegens, S.	2023	Genuine beta bursts in human working memory: controlling for the influence of lower-frequency rhythms. bioRxiv, 2023-05.	0.8	0.8	0.8		0.8			

3	Piazza, C., Dondena, C., Riboldi, E. M., Riva, V., & Cantiani, C.	2023	Baseline EEG in the first year of life: preliminary insights into the development of autism spectrum disorder and language impairments. iScience.	0.8	0.8	0.8		0.8	0.8		
4	Bonasch, H., & Ehinger, B. V.	2023	Decoding accuracies as well as ERP amplitudes do not show between-task correlations. bioRxiv, 2023-05.								
5	Guan, Q., Ma, L., Chen, Y., Luo, Y., & He, H.	2023	Midfrontal theta phase underlies evidence accumulation and response thresholding in cognitive control. Cerebral Cortex, bhad175.								
6	Li, Y., Liu, A., Yin, J., Li, C., & Chen, X.	2023	A Segmentation-Denoising Network for Artifact Removal from Single-Channel EEG. IEEE Sensors Journal.								
7	Zhen, Y., Gao, L., Chen, J., Gu, L., Shu, H., Wang, Z., ... & Zhang, Z.	2023	EEG reveals alterations in motor imagery in people with amnestic mild cognitive impairment, The Journals of Gerontology: Series B,, gbad076,								
8	Dal Bò, E	2023	Olfaction in affective disorders: an investigation of psychophysiological, behavioral and social response （Unpublished, doctoral dissertation Università degli studi Padova								
9	Miriam, C	2023	A deeper look into visual cognition: low dimensionality in visual exploration dynamics and memory retrieval （Unpublished, doctoral dissertation) Università degli studi Padova								
10	Abugaber, D., Finestrat, I., Luque, A., & Morgan-Short, K.	2023	Generalized additive mixed modeling of EEG supports dual-route accounts of morphosyntax in suggesting no word frequency effects on processing of regular grammatical forms. https://doi.org/10.1016/j.jneuroling.2023.101137								
11	Yin, Y., Chen, S., Song, T., Zhou, Q., & Shao, Y.	2023	Cognitive Load Moderates the Effects of Total Sleep Deprivation on Working Memory: Evidence from Event-Related Potentials. Brain Sciences, 13(6), 898.								
12	Kim, S., Yun, B. Y., Choi, J. Y., Kim, Y. U., & Kim, S.	2023	Quantification of visual thermal perception changes in a wooden interior environment using physiological responses and immersive virtual environment. Building and Environment, 110420.								
13	Hahn, A., Riedelsheimer, J., Royer, Z., Frederick, J., Kee, R., Crimmins, R., ... & Jantzen, K.	2023	Effects of Cleft Lip on Visual Scanning and Neural Processing of Infant Faces. Authorea Preprints.	0.6	0.6	0.6	0.6				
14	Polo, E	2023	Multimodal assessment of emotional responses by physiological monitoring: novel auditory and visual elicitation strategies in traditional and virtual reality								

			environments.（Unpublished, doctoral dissertation) Sapienza Università di Roma								
15	Del Tatto, V., Fortunato, G., Bueti, D., & Laio, A.	2023	Robust inference of causality in high-dimensional dynamical processes from the Information Imbalance of distance ranks. arXiv preprint arXiv:2305.10817.	0.9	0.9						
16	Chun, S. Y.	2023	Electrophysiological patterns of sentence superiority effect in sentence repetition.（Unpublished, doctoral dissertation) The Pennsylvania State UniversityThe Graduate School								
17	Zheng, Y., Shi, P., Deng, L., Jiang, H., & Zhou, S.	2023	Contextual valence influences the neural dynamics of time and magnitude representation during feedback evaluation. Psychophysiology, e14335.	0.95	0.85						
18	Ossandón, J. P., Stange, L., Gudi-Mindermann, H., Rimmele, J. M., Sourav, S., Bottari, D., ... & Röder, B.	2023	The development of oscillatory and aperiodic resting state activity is linked to a sensitive period in humans. NeuroImage, 120171.	0.8	0.8	0.8					
19	Motolese, F., Stelitano, D., Lanzone, J., Albergo, G., Cruciani, A., Masciulli, C., ... & NEUROFARBA, U.	2023	Feasibility and efficacy of an at-home, technology-supported mindfulness program in people with Multiple Sclerosis: a proof-of-principle study.								
20	Busch, N., Geyer, T., & Zinchenko, A.	2023	Resting State Alpha Oscillations and Inhibitory Cognitive Control: Evidence from Stroop and Navon Tasks.								
21	Yacovone, A.	2023	Grape Expectations: A collection of EEG stories on form-based prediction in natural language contexts（Unpublished, doctoral dissertation) Harvard University	0.75	0.75	0.75	0.75	0.75			
22	van Nieuwenhuizen, H., Chesebro, A. G., Polizu, C., Clarke, K., Strey, H. H., Weistuch, C., & Mujica-Parodi, L. R.	2023	Ketosis regulates K+ ion channels, strengthening brain-wide signaling disrupted by age. bioRxiv, 2023-05.								
23	Kang, K., Orlandi, S., Leung, J., Akter, M., Lorenzen, N., Chau, T., & Thaut, M. H.	2023	Electroencephalographicinterbrain synchronization in children withdisabilities, their parents, and neurologic musictherapists.European Journal of Neuroscience,1–17.https://doi.org/10.1111/ejn.16036KANGET AL.17	0.8	0.8	0.8	0.8	0.8			

24	Ody, E., Kircher, T., Straube, B., & He, Y.	2023	Pre-movement event-related potentials and multivariate pattern of EEG encode action outcome prediction.https://doi.org/10.31234/osf.io/fjk4r	0.79	0.79			0.79			
25	Schumacher, P. B., Weiland-Breckle, H., Reul, G., & Brilmayer, I.	2023	Tracking meaning evolution in the brain: Processing consequences of conventionalization. PsyArXiv, 2023-03	0.8	0.8						
26	Yu, X., Li, J., Zhu, H., Tian, X., & Lau, E.	2023	Electrophysiological hallmarks for event relations and event roles in working memory. bioRxiv, 2023-05.	0.9	0.9	0.9	0.9	0.9			
27	Arnau, S., Sharifian, F., Wascher, E., & Larra, M. F. (2023).	2023	Removing the cardiac field artifact from the EEG using neural network regression. Psychophysiology, e14323.	0.3	0.3						
28	Hinchberger, V., Kang, S. H., Kline, J., Stanley, C. J., Bulea, T. C., & Damiano, D. L.	2023	Investigation of brain mechanisms underlying upper limb function in bilateral cerebral palsy using EEG. Clinical Neurophysiology, 151, 116-127. https://doi.org/10.1016/j.clinph.2023.04.006								
29	Mygind, L., Clark, G. M., Bigelow, F. J., Fuller-Tyszkiewicz, M., Knibbs, L. D., Mavoa, S., ... & Enticott, P. G.	2023	Green enrichment for better mind readers? Residential nature and social brain function in childhood. Journal of Environmental Psychology, 88, 102029. https://doi.org/10.1016/j.jenvp.2023.102029								
30	Bagdasarov, A., Roberts, K., Brunet, D., Michel, C. M., & Gaffrey, M. S.	2023	Exploring the association between EEG microstates during resting-state and error-related activity in young children.	0.7	0.7						
31	Zhao, B., Zhang, G., Wang, L., & Dang, J.	2023	Multimodal evidence for predictive coding in sentence oral reading. Cerebral Cortex, bhad145.								
32	Astley, J., Keage, H. A., Kelson, E., Callahan, R., Hofmann, J., Thiessen, M., ... & Coussens, S.	2023	Font disfluency and reading performance in children: An event-related potential study. Brain and Cognition, 169, 105986.								
33	Ryan, D. B., Eckert, M. A., Sellers, E. W., Schairer, K. S., McBee, M. T., Ridley, E. A., & Smith, S. L.	2023	Performance Monitoring and Cognitive Inhibition during a Speech-in-Noise Task in Older Listeners. In Seminars in Hearing (Vol. 44, No. 02, pp. 124-139). Thieme Medical Publishers, Inc.. https://doi.org/10.1055/s-0043-1767695								
34	Callan, D. E., Fukada, T., Dehais, F., & Ishii, S.	2023	The role of brain-localized gamma and alpha oscillations in inattentional deafness: implications for understanding human attention. Frontiers in Human Neuroscience.								

35	Depestele, S., van Dun, K., Verstraelen, S., Ross, V., Van Hoornweder, S., Brijs, K., ... & Meesen, R.	2023	The effect of Dual-Task modality on midfrontal theta power and driving performance across the adult lifespan. Transportation Research Part F: Traffic Psychology and Behaviour, 95, 188-201. https://doi.org/10.1016/j.trf.2023.04.007								
36	Du, Y. K., Liang, M., McAvan, A. S., Wilson, R. C., & Ekstrom, A. D.	2023	Frontal-midline oscillations index the evolution of spatial memory during active navigation. bioRxiv, 2023-04.	0.9	0.9	0.9	0.9	0.9			
37	Kalantari, S., Cruz-Garza, J., Xu, T. B., Mostafavi, A., & Gao, E.	2023	Store layout design and consumer response: a behavioural and EEG study. Building Research & Information, 1-18. https://doi.org/10.1080/09613218.2023.2201415								
38	Munilla-Fajardo, J., Al-Safi, H. E., Ortiz-García, A., & Luque-Vilaseca, J. L.	2023	Hybrid Genetic Algorithm for Clustering IC Topographies of EEGs. Brain Topography, 36(3), 338-349.								
39	Joshi, Y. B., Gonzalez, C. E., Molina, J. L., MacDonald, L. R., Din, J. M., Minhas, J., ... & Light, G. A.	2023	Mismatch negativity predicts initial auditory-based targeted cognitive training performance in a heterogeneous population across psychiatric disorders. Psychiatry Research, 115215.		0.75						
40	Müller, H., Baumeister, J., Bardal, E. M., Vereijken, B., & Skjæret-Maroni, N.	2023	Exergaming in older adults: the effects of game characteristics on brain activity and physical activity. Frontiers in Aging Neuroscience, 15.								
41	Judd, C. An	2023	An Exploration of the Applications of Neurally-Informed Models of Perceptual Decision Making (Doctoral dissertation, School of Psychology at the University of Dublin, Trinity College, Ireland. 2023).								
42	Mazzeo, S., Lassi, M., Padiglioni, S., Vergani, A. A., Moschini, V., Scarpino, M., ... & Bessi, V.	2023	PRedicting the EVolution of SubjectIvE Cognitive Decline to Alzheimer's Disease With machine learning: the PREVIEW study protocol. medRxiv, 2023-04.								
43	Wu, M., Bosker, H. R., & Riecke, L.	2023	Sentential Contextual Facilitation of Auditory Word Processing Builds Up during Sentence Tracking. Journal of Cognitive Neuroscience, 1-17. https://doi.org/10.1162/jocn_a_02007								

44	Li, X., Zhou, X., Zheng, H., & Wang, C.	2023	The modulation of pain in reward processing is reflected by increased P300 and delta oscillation. Brain and Cognition, 168, 105972. https://doi.org/10.1016/j.bandc.2023.105972								
45	Quettier, T., Maffei, A., Gambarota, F., Ferrari, P. F., & Sessa, P.	2023	Testing EEG functional connectivity between sensorimotor and face processing visual regions in individuals with congenital facial palsy. Frontiers in Systems Neuroscience, 17.								
46	Meng, Z., Chen, Q., Zhou, L., Xu, L., & Chen, A.	2023	The roles of distractors in attentional blink: Impairing attentional enhancement and encoding of working memory. PsyArXiv. 2023-04 https://doi.org/10.31234/osf.io/2x86t								
47	Chiang, K. J., Dong, S., Cheng, C. K., & Jung, T. P.	2023	Using EEG signals to assess workload during memory retrieval in a real-world scenario. Journal of Neural Engineering, 20(3), 036010. https://doi.org/10.1088/1741-2552/accbed								
48	Ringer, H., Schröger, E., & Grimm, S.	2023	Neural signatures of automatic repetition detection in temporally regular and jittered acoustic sequences. bioRxiv, 2023-04.								
49	Zaky, M. H., Shoorangiz, R., Poudel, G. R., Yang, L., Innes, C. R., & Jones, R. D.	2023	Increased cerebral activity during microsleeps reflects an unconscious drive to re-establish consciousness. International Journal of Psychophysiology. https://doi.org/10.1016/j.ijpsycho.2023.05.349								
50	Krugliak, A., & Clarke, A.	2023	Translating visual perception from the lab to the real world using mobile EEG and mixed reality displays								
51	Yeh, T. C., Huang, C. C. Y., Chung, Y. A., Park, S. Y., Im, J. J., Lin, Y. Y., ... & Chang, H. A.	2023	Resting-State EEG Connectivity at High-Frequency Bands and Attentional Performance Dysfunction in Stabilized Schizophrenia Patients. Medicina, 59(4), 737.								
52	Monachesi, B., Deruti, A., Grecucci, A., & Vaes, J.	2023	Electrophysiological, emotional and behavioural responses of female targets of sexual objectification. Scientific Reports, 13(1), 5777.								
53	Ma, L., Marshall, P. J., & Wright, W. G.	2023	The order of attentional focus instructions affects how postural control processes compensate for multisensory mismatch: a crossover study. Experimental Brain Research, 1393-1409. https://doi.org/10.1007/s00221-023-06610-z	0.8	0.8		0.8	0.8			
54	Guendelman, M., Vekslar, R., & Shriki, O.	2023	Seizure dynamotype classification using non-invasive recordings. bioRxiv, 2023-04.								

55	加藤明広・堀江亮太.	2023	視認する単純な画像の形と色を反映する脳波信号特徴量とその判別の研究. 電気学会論文誌 C (電子・情報・システム部門誌), 143(4), 397-405. https://doi.org/10.1541/ieejeiss.143.397								
56	Studnicki, A., & Ferris, D. P.	2023	Parieto-Occipital Electrocortical Dynamics during Real-World Table Tennis. eneuro, 10(4).								
57	Feder, S., Miksch, J., Grimm, S., Krems, J. F., & Bendixen, A	2023	Using event-related brain potentials to evaluate motor-auditory latencies in virtual reality. PsyArXiv 2023-03	0.9	0.9						
58	Venezia, S. A.	2023	The timecourse of race and status based person perception (Doctoral dissertation, University of Delaware).								
59	Cardenas, C. H. M.	2023	Learning representative waveforms to analyze, summarize, and compare long-term neural recordings (Doctoral dissertation, University of Delaware).								
60	Ivanova, M., Neubert, C. R., Schmied, J., & Bendixen, A.	2023	ERP Evidence for Slavic and German Word Stress Cue Sensitivity in English. PsyArXiv 2023-03								
61	Song, S., Haynes, C. A., & Bradford, J. C.	2023	Human cortical, muscular, and kinematic gait adaptation with novel use of an ankle exoskeleton.								
62	López García, D.	2023	Procesado avanzado de señales de electroencefalografía y resonancia magnética en Neurociencia Cognitiva.								
63	Wu, M., Auksztulewicz, R., & Riecke, L.	2023	Multimodal acoustic-electric trigeminal nerve stimulation modulates conscious perception. bioRxiv, 2023-03.								
64	Becker, L., Büchel, D., Lehmann, T., Kehne, M., & Baumeister, J.	2023	Mobile Electroencephalography Reveals Differences in Cortical Processing During Exercises With Lower and Higher Cognitive Demands in Preadolescent Children. Pediatric Exercise Science, 1(aop), 1-11. https://doi.org/10.1123/pes.2021-0212								
65	Mohammadi, Y., Kafraj, M. S., Graversen, C., & Moradi, M. H.	2023	Decreased Resting-State Alpha Self-Synchronization in Depressive Disorder. Clinical EEG and Neuroscience,								
66	Mei, S., Deng, Y., Zheng, G., & Han, S.	2023	Shifting racial identities reduces racial ingroup biases in empathy and altruistic decision-making. PsyArXiv, 2023-03								
67	Liu, S., Liu, X., Chen, S., Su, F., Zhang, B., Ke, Y., ... & Ming, D.	2023	Neurophysiological markers of depression detection and severity prediction in first-episode major depressive disorder. Journal of Affective Disorders, 331, 8-16.								
68	Nenna, F.	2023	Human motion as a natural control of industrial robots in VR: insights on users’ performance and workload.		0.7						

			(Unpublished Doctoral dissertation, University of Padua).								
69	Coelli, S., Calcagno, A., Temporiti, F., Gatti, R., Galli, M., & Bianchi, A. M.	2023	Event-Related Desynchronization Analysis During Action Observation and Motor Imagery of Transitive Movements. scitepress								
70	Jalilpour, S., & Müller-Putz, G.	2023	Balance perturbation and error processing elicit distinct brain dynamics. Journal of Neural Engineering, 20(2), 026026.								
71	Sabo, M., Wascher, E., & Schneider, D.	2023	Remembering what is relevant: how is goal-directed memory reactivation supported by attention and working memory?. bioRxiv, 2023-03.		0.3						
72	Ozkan, A., Uyan, U., & Celikcan, U.	2023	Effects of speed, complexity and stereoscopic VR cues on cybersickness examined via EEG and self-reported measures. Displays, 78, 102415. https://doi.org/10.1016/j.displa.2023.102415								
73	Valente, I. M. S.	2023	Is there evidence for resting-state EEG interhemispheric imbalance in people with depression? A pilot study (Unpublished master’s thesis).								
74	Ringer, H., Schröger, E., & Grimm, S.	2023	Perceptual learning of random acoustic patterns: Impact of temporal regularity and attention. European Journal of Neuroscience.								
75	Bierwirth, P., Antov, M. I., & Stockhorst, U.	2023	Oscillatory and non-oscillatory brain activity reflects fear expression in an immediate and delayed fear extinction task. Psychophysiology, e14283.	0.9							
76	Chueh, T. Y., Hung, C. L., Chang, Y. K., Huang, C. J., & Hung, T. M.	2023	Effects of cognitive demand during acute exercise on inhibitory control and its electrophysiological indices: A randomized crossover study. Physiology & Behavior, 265, 114148. https://doi.org/10.1016/j.physbeh.2023.114148								
77	Chiossi, F., Ou, C., & Mayer, S.	2023	Exploring Physiological Correlates of Visual Complexity Adaptation: Insights from EDA, ECG, and EEG Data for Adaptation Evaluation in VR Adaptive Systems. In Extended Abstracts of the 2023 CHI Conference on Human Factors in Computing Systems (pp. 1-7).								
78	Afzali, A., Khaleghi, A., Hatef, B., Akbari Movahed, R., & Pirzad Jahromi, G.	2023	Automated major depressive disorder diagnosis using a dual-input deep learning model and image generation from EEG signals. Waves in Random and Complex Media, 1-16. https://doi.org/10.1080/17455030.2023.2187237								
79	Popov, T., Tröndle, M., Baranczuk-Turska, Z., Pfeiffer,	2023	Test–retest reliability of resting-state EEG in young and older adults. Psychophysiology, e14268.	0.8	0.8	0.8	0.8	0.8			

	C., Haufe, S., & Langer, N.										
80	Sherman, D. A., Baumeister, J., Stock, M. S., Murray, A. M., Bazett-Jones, D. M., & Norte, G. E.	2023	Brain activation and single-limb balance following anterior cruciate ligament reconstruction. Clinical Neurophysiology, 149, 88-99. https://doi.org/10.1016/j.clinph.2023.02.175								
81	Johari, K., & Berger, J. I.	2023	High-definition transcranial direct current stimulation over right dorsolateral prefrontal cortex differentially modulates inhibitory mechanisms for speech vs. limb movement. Psychophysiology, e14289. https://doi.org/10.1111/psyp.14289								
82	Vlieger, R., Daskalaki, E., Apthorp, D., Lueck, C. J., & Suominen, H.	2023	Evaluating Effects of Resting-State Electroencephalography Data Pre-Processing on a Machine Learning Task for Parkinson’s Disease. medRxiv, 2023-03.								0.7
83	Heffer, T., van Noordt, S., & Willoughby, T.	2023	Developmental trajectories of sensitivity to threat in children and adolescents predict larger medial frontal theta differentiation during response inhibition. Social Cognitive and Affective Neuroscience, 18(1), nsad009.								
84	Constant, M., Mandal, A., Asanowicz, D., Yamaguchi, M., Gillmeister, H., Kerzel, D., ... & Liesefeld, H. R.	2023	A multilab investigation into the N2pc as an indicator of attentional selectivity: Direct replication of Eimer (1996). PsyArXiv, 2023-03		0.8						
85	Domic-Siede, M., Irani, M., Valdés, J., Rodríguez, M., Follet, B., Perrone-Bertolotti, M., & Ossandón, T.	2023	A Visuospatial Planning Task Coupled with Eye-Tracker and Electroencephalogram Systems. JoVE (Journal of Visualized Experiments), (193), e64622.								
86	Depestele, S., van Dun, K., Verstraelen, S., Van Hoornweder, S., & Meesen, R.	2023	Midfrontal theta and cognitive control during interlimb coordination across the adult lifespan. Journal of Motor Behavior, 55(3), 278-288. https://doi.org/10.1080/00222895.2023.2183178								
87	Peskar, M., Omejc, N., Šömen, M. M., Miladinović, A., Gramann, K., & Marusic, U.	2023	Stroop in motion: Neurodynamic modulation underlying interference control while sitting, standing, and walking. Biological psychology, 178, 108543.		0.85						
88	Mendoza-Cardenas, C. H., Meek, A., & Brockmeier, A. J.	2023	Labeling EEG Components with a Bag of Waveforms from Learned Dictionaries. In ICLR 2023 Workshop on Time Series Representation Learning for Health.								

89	Sagehorn, M., Johnsdorf, M., Kisker, J., Sylvester, S., Gruber, T., & Schöne, B.	2023	Real-life relevant face perception is not captured by the N170 but reflected in later potentials: A comparison of 2D and virtual reality stimuli. Frontiers in Psychology, 14.	0.9	0.8	0.9	0.9	0.9			
90	Presti, P., Galasso, G. M., Ruzzon, D., Avanzini, P., Caruana, F., Rizzolatti, G., & Vecchiato, G.	2023	Architectural experience influences the processing of others' body expressions. bioRxiv, 2023-02.								
91	Nakanishi, M., & Miyakoshi, M.	2023	Revisiting Polarity Indeterminacy of ICA-Decomposed ERPs and Scalp Topographies. Brain Topography, 36(2), 223-229. https://doi.org/10.1007/s10548-023-00944-1							0.7	
92	Ballesteros, A. S., Prado, P., Ibanez, A., Perez, J. A. M., & Moguilner, S.	2023	A pipeline for large-scale assessments of dementia EEG connectivity across multicentric settings.OSF Preprints. 2023-02								
93	Uyulan, Ç., Mayor, D., Steffert, T., Watson, T., & Banks, D.	2023	Classification of the Central Effects of Transcutaneous Electroacupuncture Stimulation (TEAS) at Different Frequencies: A Deep Learning Approach Using Wavelet Packet Decomposition with an Entropy Estimator. Applied Sciences, 13(4), 2703.								
94	Yeh, T. C., Huang, C. C. Y., Chung, Y. A., Park, S. Y., Im, J. J., Lin, Y. Y., ... & Chang, H. A.	2023	Online Left-Hemispheric In-Phase Frontoparietal Theta tACS Modulates Theta-Band EEG Source-Based Large-Scale Functional Network Connectivity in Patients with Schizophrenia: A Randomized, Double-Blind, Sham-Controlled Clinical Trial. Biomedicines, 11(2), 630.								
95	Zhou, P., Wu, Q., Zhan, L., Guo, Z., Wang, C., Wang, S., ... & Wu, X.	2023	Alpha peak activity in resting-state EEG is associated with depressive score. Frontiers in Neuroscience, 17.								
96	Bates, K. E., Smith, M. L., Farran, E. K., & Machizawa, M. G	2023	Behavioural and neural correlates of visual working memory reveals metacognitive aspects of mental imagery. PsyArXiv, 2023-02								
97	Bailey, N. W., Biabani, M., Hill, A. T., Miljevic, A., Rogasch, N. C., McQueen, B., ... & Fitzgerald, P. B.	2023	Introducing RELAX: An automated pre-processing pipeline for cleaning EEG data-Part 1: Algorithm and application to oscillations. Clinical Neurophysiology, 149, 178-201.								

98	Bailey, N. W., Hill, A. T., Biabani, M., Murphy, O. W., Rogasch, N. C., McQueen, B., ... & Fitzgerald, P. B.	2023	RELAX part 2: A fully automated EEG data cleaning algorithm that is applicable to Event-Related-Potentials. Clinical Neurophysiology, 149, 202-222.								
99	Taheri Gorji, H., Wilson, N., VanBree, J., Hoffmann, B., Petros, T., & Tavakolian, K.	2023	Using machine learning methods and EEG to discriminate aircraft pilot cognitive workload during flight. Scientific Reports, 13(1), 2507.		0.8						
100	Bailey, N., Baell, O., Payne, J., Humble, G., Geddes, H., Cahill, I., ... & Fitzgerald, P.	2023	Experienced Meditators Show Multifaceted Attention-Related Differences in Neural Activity. bioRxiv, 2023-02.								
101	Turoman, N., Fiave, P. A., Zahnd, C., deBettencourt, M. T., & Vergauwe, E.	2023	Decoding the content of working memory in school-aged children. bioRxiv, 2023-02.								
102	Paek, A. Y., & Prashad, S.	2023	Repetitive execution of a reach-and-lift task causes longitudinal attenuation in movement-related EEG features. bioRxiv, 2023-02.								
103	Bakhtiari, A., Petersen, J., Urdanibia-Centelles, O., Ghazi, M. M., Fagerlund, B., Mortensen, E. L., ... & Benedek, K.	2023	Power and distribution of evoked gamma oscillations in brain aging and cognitive performance. GeroScience, 1-16. https://doi.org/10.1007/s11357-023-00749-x	0.95	0.9						
104	Song, T., Du, F., Xu, L., Peng, Z., Wang, L., Dai, C., ... & Li, S.	2023	Total sleep deprivation selectively impairs motor preparation sub-stages in visual search task: Evidence from lateralized readiness potentials. Frontiers in Neuroscience, 17, 989512.								
105	Delorme, A.	2023	EEG is better left alone. Scientific reports, 13(1), 2372.								
106	Hinss, M. F., Jahanpour, E. S., Somon, B., Pluchon, L., Dehais, F., & Roy, R. N.	2023	Open multi-session and multi-task EEG cognitive Dataset for passive brain-computer Interface Applications. Scientific Data, 10(1), 85.	0.9	0.9	0.9					
107	Massaeli, F., Bagheri, M., & Power, S. D.	2023	EEG-based detection of modality-specific visual and auditory sensory processing. Journal of Neural Engineering, 20(1), 016049.								

108	Sibilano, E., Brunetti, A., Buongiorno, D., Lassi, M., Grippo, A., Bessi, V., ... & Bevilacqua, V.	2023	An attention-based deep learning approach for the classification of subjective cognitive decline and mild cognitive impairment using resting-state EEG. Journal of Neural Engineering, 20(1), 016048.								
109	Kumar G, P., Sharma, K., Manvi, A., & Angarai, R. G.	2023	Coherence-based interhemispheric EEG functional connectivity changes in distinct frequency bands during eyes open meditation. bioRxiv, 2023-02.	0.7	0.7	0.5					
110	Alyan, E., Wascher, E., Arnau, S., Kaesemann, R., & Reiser, J. E.	2023	Operator State in a Workplace Simulation Modulates Eye-Blink Related EEG Activity. IEEE Transactions on Neural Systems and Rehabilitation Engineering, 31, 1167-1179.	0.3	0.3	0.3	0.3	0.3	0.3		0.3
111	Chen, Y., Fazli, S., & Wallraven, C.	2023	Decoding deceipt: EEG signatures of lying behavior under spontaneous versus instructed lying and truth-telling in a two-player game. https://doi.org/10.21203/rs.3.rs-2521275/v1		0.7						
112	Ross, J. M., Cline, C. C., Sarkar, M., Truong, J., & Keller, C. J.	2023	Neural effects of TMS trains on the human prefrontal cortex. bioRxiv, 2023-01.								
113	Xu, T., Zhao, T., Cruz-Garza, J. G., Bhattacharjee, T., & Kalantari, S.	2023	Evaluating Human-in-the-Loop Assistive Feeding Robots Under Different Levels of Autonomy with VR Simulation and Physiological Sensors. In Social Robotics: 14th International Conference, ICSR 2022, Florence, Italy, December 13–16, 2022, Proceedings, Part II (pp. 314-327). Cham: Springer Nature Switzerland.								
114	Railo, H., Kraufvelin, N., Santalahti, J., & Laine, T.	2023	Rapid withdrawal from threatening animals is movement-specific and mediated by reflex-like neural processing. bioRxiv, 2023-01.							0.7	
115	Omejc, N., Peskar, M., Miladinović, A., Kavcic, V., Džeroski, S., & Marusic, U.	2023	On the Influence of Aging on Classification Performance in the Visual EEG Oddball Paradigm Using Statistical and Temporal Features. Life, 13(2), 391.	0.85	0.85	0.85	0.85	0.85	0.85		
116	Iwama, S	2023	Cortico-cortical coupling of neural oscillations underlying human sensorimotor control. (Unpublished Doctoral dissertation, Keio University).								
117	Plechawska-Wójcik, M., Augustynowicz, P., Kaczorowska, M., Zabielska-Mendyk, E., & Zapała, D.	2023	The Influence Assessment of Artifact Subspace Reconstruction on the EEG Signal Characteristics. Applied Sciences, 13(3), 1605.								

118	Mastropietro, A., Pirovano, I., Marciano, A., Porcelli, S., & Rizzo, G.	2023	Reliability of Mental Workload Index Assessed by EEG with Different Electrode Configurations and Signal Pre-Processing Pipelines. Sensors, 23(3), 1367.								0.4
119	Cheng, B., Lin, E., Wunderlich, A., Gramann, K., & Fabrikant, S. I.	2023	Using eye blink-related brain activity to investigate cognitive load during assisted navigation. Frontiers in Neuroscience, 17, 152.								0.3
120	Marzuki, A. A., Gloy, K., Kandler, C., Yip, W. Z., Wong, K. Y., Phon-Amnuaisuk, P., ... & Schaefer, A.	2023	Error-and inhibitory-related brain activity associated with political ideology: A multi-site replication study. PsyArXiv 2023-01 https://doi.org/10.31234/osf.io/39vqj								
121	Hucke, C. I., Heinen, R. M., Wascher, E., & van Thriel, C.	2023	Trigeminal stimulation is required for neural representations of bimodal odor localization: A time-resolved multivariate EEG and fNIRS study. NeuroImage, 269, 119903.								
122	Rho, G., Callara, A. L., Bernardi, G., Scilingo, E. P., & Greco, A.	2023	EEG cortical activity and connectivity correlates of early sympathetic response during cold pressor test. Scientific Reports, 13(1), 1338.								
123	Bangel, K. A., Bais, M., Eijsker, N., Schuurman, P. R., van den Munckhof, P., Figee, M., ... & Denys, D.	2023	Acute effects of deep brain stimulation on brain function in obsessive–compulsive disorder. Clinical Neurophysiology, 148, 109-117.	0.5	0.5	0.5	0.5	0.5			
124	Gil Avila, C., Bott, F. S., Tiemann, L., Hohn, V. D., May, E. S., Nickel, M. M., ... & Ploner, M.	2023	DISCOVER-EEG: an open, fully automated EEG pipeline for biomarker discovery in clinical neuroscience. bioRxiv, 2023-01.								
125	Gogulski, J., Cline, C. C., Ross, J. M., Truong, J., Sarkar, M., Parmigiani, S., & Keller, C. J.	2023	Mapping cortical excitability in the human dorsolateral prefrontal cortex. bioRxiv, 2023-01.								
126	Song, S., & Nordin, A. D.	2023	Balance Perturbations in Simulated Low-Gravity Modulate Human Premotor and Frontoparietal Electrocortical Theta, Alpha, and Beta Band Spectral Power. IEEE Open Journal of Engineering in Medicine and Biology. EEE Open Journal of Engineering in Medicine and Biology								

127	O'Keeffe, R., Rathod, V., Shirazi, S. Y., Mehrdad, S., Edwards, A., Rao, S., & Atashzar, S. F.	2023	Linear versus Nonlinear Muscle Networks: A Case Study to Decode Hidden Synergistic Patterns During Dynamic Lower-limb Tasks. bioRxiv, 2023-01.								
128	Davis, M. C., Fitzgerald, P. B., Bailey, N. W., Sullivan, C., Stout, J. C., Hill, A. T., & Hoy, K. E	2023	Effects of medial prefrontal transcranial alternating current stimulation on neural activity and connectivity in people with Huntington’s disease and neurotypical controls. Brain Research, 1811, 148379. https://doi.org/10.1016/j.brainres.2023.148379								
129	Wisniewski, M. G., Joyner, C. N., Zakrzewski, A. C., & Anguiano, A.	2023	Learning to detect auditory signals in noise: Active top-down selection and stable change in signal representations. Journal of Experimental Psychology: Human Perception and Performance. 49(3), 428–440.	0.5	0.5		0.5	0.5			
130	Truong, D., Robbins, K., Delorme, A., & Makeig, S.	2023	End-to-end Processing of M/EEG Data with BIDS, HED, and EEGLAB. OSF Preprints.OSF Preprints. 2023-01. https://doi.org/10.31219/osf.io/h7puk								
131	Wang, X., Yang, Q., Zhai, Y., Niu, H., & Wang, X.	2023	Effects of Vehicle Air Temperature on Drivers’ Cognitive Abilities Based on EEG. Sustainability, 15(2), 1673.								
132	Liu, C., Downey, R. J., Mu, Y., Richer, N., Hwang, J., Shah, V. A., ... & Ferris, D. P.	2023	Comparison of EEG source localization using simplified and anatomically accurate head models in younger and older adults. https://doi.org/10.1109/TNSRE.2023.3281356							0.5	
133	Gonsisko, C. B., Ferris, D. P., & Downey, R. J.	2023	iCanClean Improves Independent Component Analysis of Mobile Brain Imaging with EEG. Sensors, 23(2), 928.							0.5	
134	Zolezzi, D. M., Alonso-Valerdi, L. M., & Ibarra-Zarate, D. I.	2023	EEG frequency band analysis in chronic neuropathic pain: a linear and nonlinear approach to classify pain severity. Computer Methods and Programs in Biomedicine, 107349.								
135	Sadus, K., Schubert, A. L., Löffler, C., & Hagemann, D	2023	A multiverse study for extracting differences in P3 latencies between young and old adults. PsyArXiv, 2023-01 https://doi.org/10.31234/osf.io/pfza5								0.5
136	Wascher, E., Alyan, E., Karthaus, M., Getzmann, S., Arnau, S., & Reiser, J. E.	2023	Tracking drivers’ minds: Continuous evaluation of mental load and cognitive processing in a realistic driving simulator scenario by means of the EEG. OSF Preprints. 2023-01. doi:10.31219/osf.io/se5nz.								
137	Zapata-Saldarriaga, L. M., Vargas-Serna, A. D., Gil-Gutiérrez, J., Mantilla-Ramos, Y.	2023	Evaluation of Strategies Based on Wavelet-ICA and ICLabel for Artifact Correction in EEG Recordings. Revista Científica, 46(1), 61-76.								

	J., & Ochoa-Gómez, J. F.										
138	Lassi, M., Fabbiani, C., Mazzeo, S., Burali, R., Vergani, A. A., Giacomucci, G., ... & Mazzoni, A.	2023	Degradation of EEG microstates patterns in subjective cognitive decline and mild cognitive impairment: Early biomarkers along the Alzheimer’s Disease continuum?. NeuroImage: Clinical, 38, 103407.							0.75	
139	Lu, L., Yang, J., Shu, R., & Long, C.	2023	The default-interventionist model underlies premise typicality weakening the premise diversity effect during category-based induction: Event-related potentials evidence. Scandinavian Journal of Psychology. https://doi-org.osaka-u.idm.oclc.org/10.1111/sjop.12892	0.7	0.7	0.7	0.7	0.7	0.7		
140	Chen, H., He, H., Zhu, J., Sun, S., Li, J., Shao, X., & Li, J.	2023	Weight-based Channel-model Matrix Framework provides a reasonable solution for EEG-based cross-dataset emotion recognition								
141	Stuldreher, I. V., Kaneko, D., Hiraguchi, H., van Erp, J. B., & Brouwer, A. M.	2023	EEG measures of attention toward food-related stimuli vary with food neophobia. Food quality and preference, 106, 104805.								
142	Kim, H., Miyakoshi, M., Kim, Y., Stapornchaisit, S., Yoshimura, N., & Koike, Y	2023	Electroencephalography Reflects User Satisfaction in Controlling Robot Hand through Electromyographic Signals. Sensors, 23(1), 277.								
143	Fang, L., Andrzejewski, J. A., & Carlson, J. M.	2023	The gray matter morphology associated with the electrophysiological response to errors in individuals with high trait anxiety. International Journal of Psychophysiology, 184, 76-83. https://doi.org/10.1016/j.ijpsycho.2022.12.007								
144	Penalver-Andres, J. A., Buetler, K. A., Koenig, T., Müri, R. M., & Marchal-Crespo, L.	2023	Resting-State Functional Networks Correlate with Motor Performance in a Complex Visuomotor Task: An EEG Microstate Pilot Study on Healthy Individuals. Brain topography, 1-18.								0.4
145	Wang, W., & Li, B.	2023	A novel model based on a 1D-ResCNN and transfer learning for processing EEG attenuation. Computer Methods in Biomechanics and Biomedical Engineering, 1-14. https://doi-org.osaka-u.idm.oclc.org/10.1080/10255842.2022.2162339								
146	Dillen, A., Ghaffari, F., Romain, O., Vanderborght, B., Marusic, U.,	2023	Optimal sensor set for decoding motor imagery from EEG. Applied Sciences, 13(7), 4438.								

	Grosprêtre, S., ... & De Pauw, K.										
147	Nikolaev, A. R., Bramao, I., Johansson, R., & Johansson, M.	2023	Episodic memory formation in unrestricted viewing. NeuroImage, 266, 119821.	0.4	0.9	0.05	0.4	0.4	0.4		
148	Nguyen, T. N. Q., Vo, H. T. T., Nguyen, H. A., & Van Huynh, T.	2023	Machine Learning in Classification of Parkinson’s Disease Using Electroencephalogram with Simon’s Conflict. In Computational Intelligence Methods for Green Technology and Sustainable Development: Proceedings of the International Conference GTSD2022 (pp. 110-122). Cham: Springer International Publishing.								
149	Bao, Z., & Frewen, P.	2023	Sense of self in mind and body: an eLORETA-EEG study. Neuroscience of Consciousness, 2022(1), niac017.							0.7	
150	Dück, K., Overmeyer, R., Mohr, H., & Endrass, T.	2023	Are electrophysiological correlates of response inhibition linked to impulsivity and compulsivity? A machine-learning analysis of a Go/Nogo task. Psychophysiology, e14310.								
151	Bhattacharyya, A., Verma, A., Ranta, R., & Pachori, R. B.	2023	Ocular artifacts elimination from multivariate EEG signal using frequency-spatial filtering. IEEE Transactions on Cognitive and Developmental Systems. , doi: 10.1109/TCDS.2022.3226775.								
152	Depestele, S., van Dun, K., Verstraelen, S., Ross, V., Van Hoornweder, S., Brijs, K., ... & Meesen, R	2023	Age-related changes in midfrontal theta activity during steering control: A driving simulator study. Neurobiology of aging, 123, 145-153. https://doi.org/10.1016/j.neurobiolaging.2022.11.014								
153	McInnes, A. N., Sung, B., & Hooshmand, R.	2023	A practical review of electroencephalography’s value to consumer research. International Journal of Market Research, 65(1), 52-82. https://doi.org/10.1177/14707853221112622								
154	Mostafavi, A., Cruz-Garza, J., & Kalantari, S.	2023	Enhancing lighting design through the investigation of illuminance and correlated color Temperature's effects on brain activity: An EEG-VR approach. Journal of Building Engineering, 106776. https://doi.org/10.1016/j.jobe.2023.106776								
155	Perera, M. P. N., Mallawaarachchi, S., Bailey, N. W., Murphy, O. W., & Fitzgerald, P. B.	2023	Obsessive-compulsive disorder (OCD) is associated with increased electroencephalographic (EEG) delta and theta oscillatory power but reduced delta connectivity. Journal of Psychiatric Research.								

156	Davis, M. C., Hill, A. T., Fitzgerald, P. B., Bailey, N. W., Sullivan, C., Stout, J. C., & Hoy, K. E.	2023	Medial prefrontal transcranial alternating current stimulation for apathy in Huntington's disease. Progress in Neuro-Psychopharmacology and Biological Psychiatry, 126, 110776. https://doi.org/10.1016/j.pnpbp.2023.110776								
157	Ringer, H., Schröger, E., & Grimm, S.	2023	Within-and between-subject consistency of perceptual segmentation in periodic noise: A combined behavioral tapping and EEG study. Psychophysiology, 60(2), e14174.								
158	Schubert, A. L., Löffler, C., Hagemann, D., & Sadus, K.	2023	How robust is the relationship between neural processing speed and cognitive abilities?. Psychophysiology, 60(2), e14165.								
159	Kotowski, K., Ochab, J., Stapor, K., & Sommer, W.	2023	The importance of ocular artifact removal in single-trial ERP analysis: The case of the N250 in face learning. Biomedical Signal Processing and Control, 79, 104115.								
160	Rosenkranz, M., Cetin, T., Uslar, V. N., & Bleichner, M. G.	2023	Investigating the attentional focus to workplace-related soundscapes in a complex audio-visual-motor task using EEG. Frontiers in Neuroergonomics, 3, 38.	0.9	0.9	0.9					
161	Patelaki, E., Foxe, J. J., Mantel, E. P., Kassis, G., & Freedman, E. G.	2023	Paradoxical improvement of cognitive control in older adults under dual-task walking conditions is associated with more flexible reallocation of neural resources: A Mobile Brain-Body Imaging (MoBI) study. NeuroImage, 273, 120098.								
162	Bullock, T., MacLean, M. H., Santander, T., Boone, A. P., Babenko, V., Dundon, N. M., ... & Grafton, S. T.	2023	Habituation of the stress response multiplex to repeated cold pressor exposure. Frontiers in Physiology, 13, 2542.							0.7	
163	Haaf, M., Curic, S., Rauh, J., Steinmann, S., Mulert, C., & Leicht, G.	2023	Opposite Modulation of the NMDA Receptor by Glycine and S-Ketamine and the Effects on Resting State EEG Gamma Activity: New Insights into the Glutamate Hypothesis of Schizophrenia. International Journal of Molecular Sciences, 24(3), 1913.							0.5	
164	Klatt, L. I., Begau, A., Schneider, D., Wascher, E., & Getzmann, S.	2023	Cross-modal interactions at the audiovisual cocktail-party revealed by behavior, ERPs, and neural oscillations. NeuroImage, 271, 120022.		0.3						0.3
165	Kastrati, A., Plomecka, M. B., Küchler, J., Langer, N., & Wattenhofer, R.	2023	Electrode clustering and bandpass analysis of eeg data for gaze estimation. In Annual Conference on Neural Information Processing Systems (pp. 50-65). PMLR.								

166	Liu, Z., Zhang, Z., Liu, Z., Li, S., & Liu, S.	2023	Altered neural correlates of optimal decision-making in individuals with depressive status. Biological Psychology, 176, 108462. https://doi.org/10.1016/j.biopsycho.2022.108462								
167	Okdahl, T., Mark, E. B., Nedergaard, R. B., Knoph, C. S., Cook, M. E., Krogh, K., & Drewes, A. M.	2023	Effects of opium tincture on the enteric and central nervous systems: A randomized controlled trial. Basic & Clinical Pharmacology & Toxicology, 132(5), 434-448.								
168	Wong, Y. S., Pat, N., & Machado, L.	2023	Commonalities between mind wandering and task-set switching: An event-related potential study. Neuropsychologia, 108585.								
169	Tröndle, M., Popov, T., Pedroni, A., Pfeiffer, C., Barańczuk-Turska, Z., & Langer, N.	2023	Decomposing age effects in EEG alpha power. Cortex, 161, 116-144.	0.8	0.8	0.8	0.8	0.8			
170	Bower, I. S., Hill, A. T., & Enticott, P. G.	2023	Functional brain connectivity during exposure to the scale and color of interior built environments. Human Brain Mapping, 44(2), 447-457.								0.3
171	Orłowski, P., & Bola, M.	2023	Sensory modality defines the relation between EEG Lempel–Ziv diversity and meaningfulness of a stimulus. Scientific Reports, 13(1), 3453.	0.6	0.6	0.6	0.6	0.6	0.6		
172	Zhang, G., Wu, G., & Yang, J.	2023	The restorative effects of short-term exposure to nature in immersive virtual environments (IVEs) as evidenced by participants' brain activities. Journal of Environmental Management, 326, 116830. https://doi.org/10.1016/j.jenvman.2022.116830								
173	Billeci, L., Callara, A. L., Guiducci, L., Prosperi, M., Morales, M. A., Calderoni, S., ... & Santocchi, E.	2023	A randomized controlled trial into the effects of probiotics on electroencephalography in preschoolers with autism. Autism, 27(1), 117-132.								
174	Getzmann, S., Schneider, D., & Wascher, E.	2023	Selective spatial attention in lateralized multi-talker speech perception: EEG correlates and the role of age. Neurobiology of Aging, 126, 1-13. https://doi.org/10.1016/j.neurobiolaging.2023.02.003								
175	Patelaki, E., Foxe, J. J., Mazurek, K. A., & Freedman, E. G.	2023	Young adults who improve performance during dual-task walking show more flexible reallocation of cognitive resources: a mobile brain-body imaging (MoBI) study. Cerebral cortex, 33(6), 2573-2592.	0.5	0.5	0.5	0.5	0.5			
176	Strzelczyk, D., Kelly, S. P., & Langer, N.	2023	Neurophysiological markers of successful learning in healthy aging. GeroScience, 1-24.	0.8	0.8	0.8	0.8	0.8			

177	Korochkina, M., Sowman, P. F., Nickels, L., & Bürki, A.	2023	Neural correlates of encoding in novel word learning. Collabra: Psychology, 9(1), 57525.								
178	Lasagna, C. A., Grove, T. B., Semple, E., Suzuki, T., Menkes, M. W., Pamidighantam, P., ... & Tso, I. F.	2023	Reductions in regional theta power and fronto-parietal theta-gamma phase-amplitude coupling during gaze processing in bipolar disorder. Psychiatry Research: Neuroimaging, 331, 111629. https://doi.org/10.1016/j.pscychresns.2023.111629								
179	Morgan-Short, K., Abugaber, D., Finestrat, I., & Luque, A.	2023	Generalized additive mixed modeling of EEG supports dual-route accounts of morphosyntax in suggesting no word frequency effects on processing of regular grammatical forms.PsyArXiv, 2023-03								
180	Cotter, M., Reisli, S., Francisco, A. A., Wakim, K. M., Oakes, L., Crosse, M. J., ... & Molholm, S.	2023	Neurophysiological measures of auditory sensory processing are associated with adaptive behavior in children with Autism Spectrum Disorder. Journal of Neurodevelopmental Disorders, 15(1), 1-14.		0.8						0.05

Summary. Numbers of publications citing the original paper of the ICLabel pretrained classifer by Pion-Tonachini et al. (2019), categorized by those reporting thresholds, missing thresholds, and those we could not download.

**Year**	**Reporting**	**Missing**	**Undownloadable**	**Total**
**2019**	4	11	4	19
**2020**	16	37	7	60
**2021**	53	102	29	184
**2022**	98	126	42	266
**2023**	68	80	32	180
**Total**	239	356	114	709

Notes:

1. The above numbers are as of June 8, 2023.

2. Duplicates have been removed.

3. "Reporting" include articles in which thresholds were unclear

Supplementary Table S2. Mean intraclass correlation coefficients of microstate metrics for outside-EEG in resting-state and oddball tasks for each ICLabel threshold considered.

**Resting state (4 templates)**				
**Thresholds (Mean spatial correlation)***	**Duration**	**Occurrence**	**Coverage**	**Transition**
**.60** (.990)**	.782	.730	.677	.580
**.65 (.973)**	.679	.612	.445	.369
**.70 (.925)**	.475	.196	.166	.217
**.75 (.913)**	.666	.567	.412	.186
**.80 (.975)**	.732	.657	.574	.451
**Resting state (5 templates)**				
**Thresholds (Mean spatial correlation)**	**Duration**	**Occurrence**	**Coverage**	**Transition**
**.60 (.929)**	.653	.586	.452	.305
**.65 (.977)**	.770	.674	.627	.493
**.70 (.988)**	.798	.631	.604	.425
**.75 (.903)**	.703	.590	.494	.253
**.80 (.975)**	.664	.575	.522	.294
**Oddball (4 templates)**				
**Thresholds (Mean spatial correlation)**	**Duration**	**Occurrence**	**Coverage**	**Transition**
**.60 (.967)**	.668	.594	.482	.337
**.65 (.984)**	.762	.743	.677	.480
**.70 (.939)**	.672	.493	.346	.202
**.75 (.987)**	.765	.751	.654	.554
**.80** (.991)**	.785	.796	.710	.506
**Oddball (5 templates)**				
**Thresholds (Mean spatial correlation)**	**Duration**	**Occurrence**	**Coverage**	**Transition**
**.60 (.889)**	.639	.408	.276	.099
**.65 (.972)**	.732	.752	.673	.375
**.70 (.989)**	.797	.725	.712	.414
**.75 (.988)**	.808	.785	.745	.476
**.80 (.937)**	.644	.545	.386	.135

* A mean of spatial correlation coefficients between each pair of microstate templates.

** The ICLabel threshold selected in the main text.

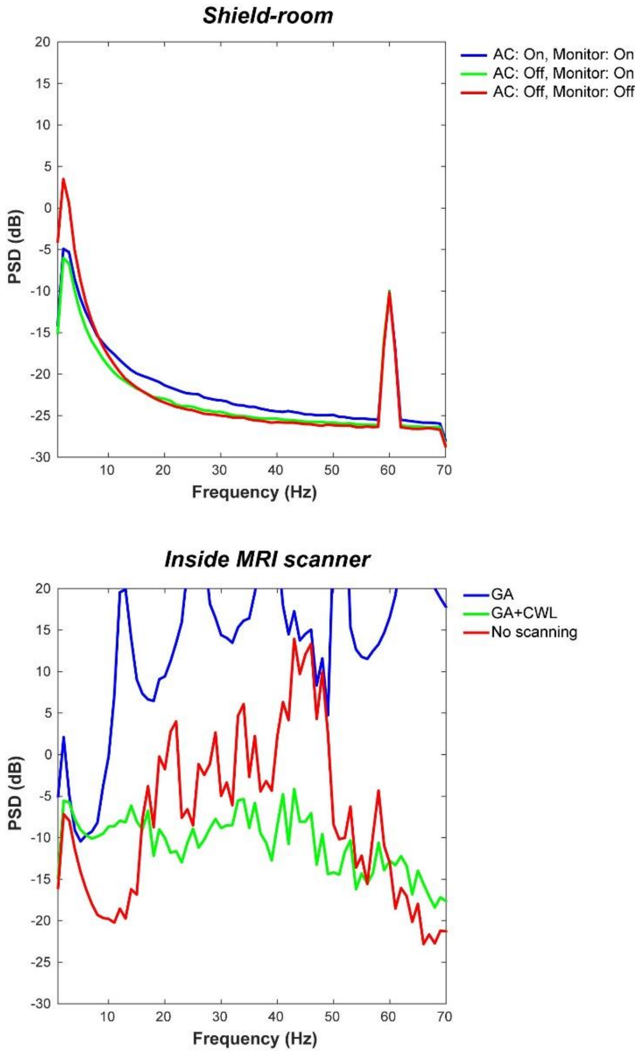

Supplementary Figure S1. PSD of "EEG" recorded for 5 min with a phantom (watermelon) in the shield room (top) and inside the MRI scanner (bottom). Ceiling lights remained on throughout in the shield room whereas a helium pump remained on throughout in the scanner. AC: air conditioner; GA: gradient artifact; CWL: carbon-wire loop

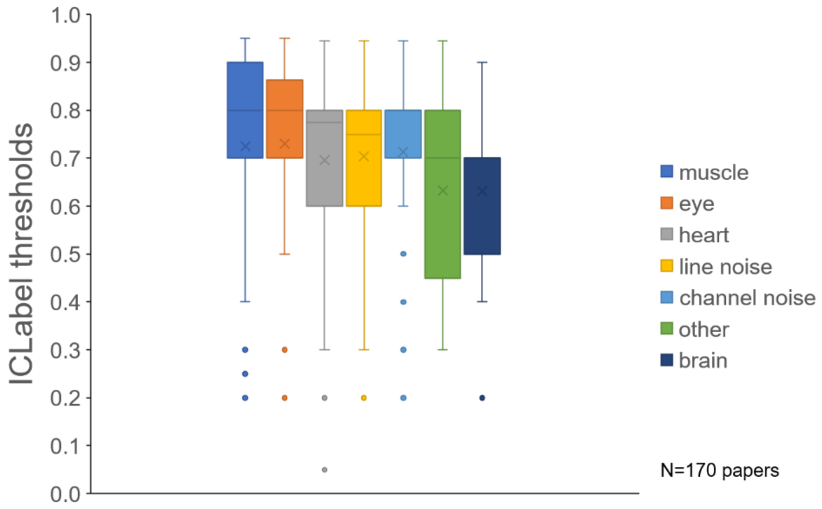

Supplementary Figure S2. A box-whisker plot summarizing ICLabel thresholds used in a set of 170 studies reporting the thresholds in a clear way. Cross marks represent means. Dots represent outliers.

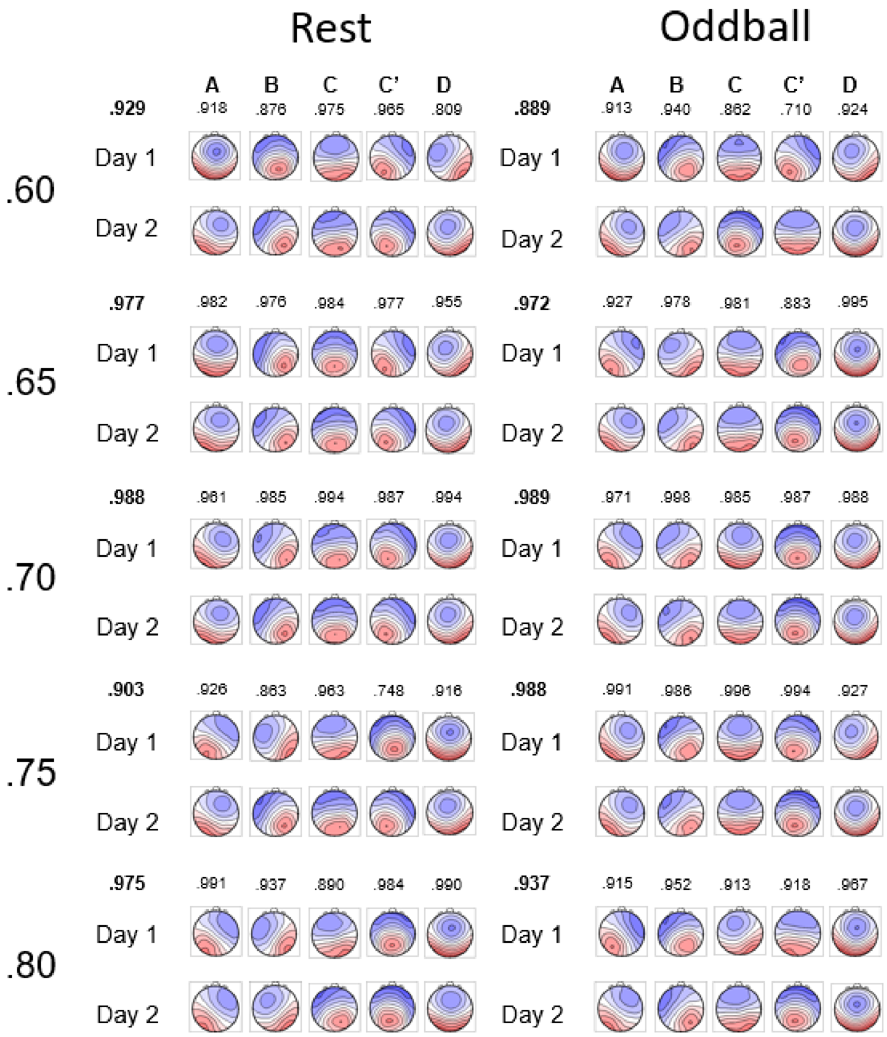

Supplementary Figure S3. Each set of five microstate templates for outside-EEG in resting-state (left) and oddball tasks (right). The values from .60 to .80 represent ICLabel thresholds. The value above a pair of the same type of template (e.g., A) indicates spatial correlation coefficient, with their mean in bold texts above Day 1.

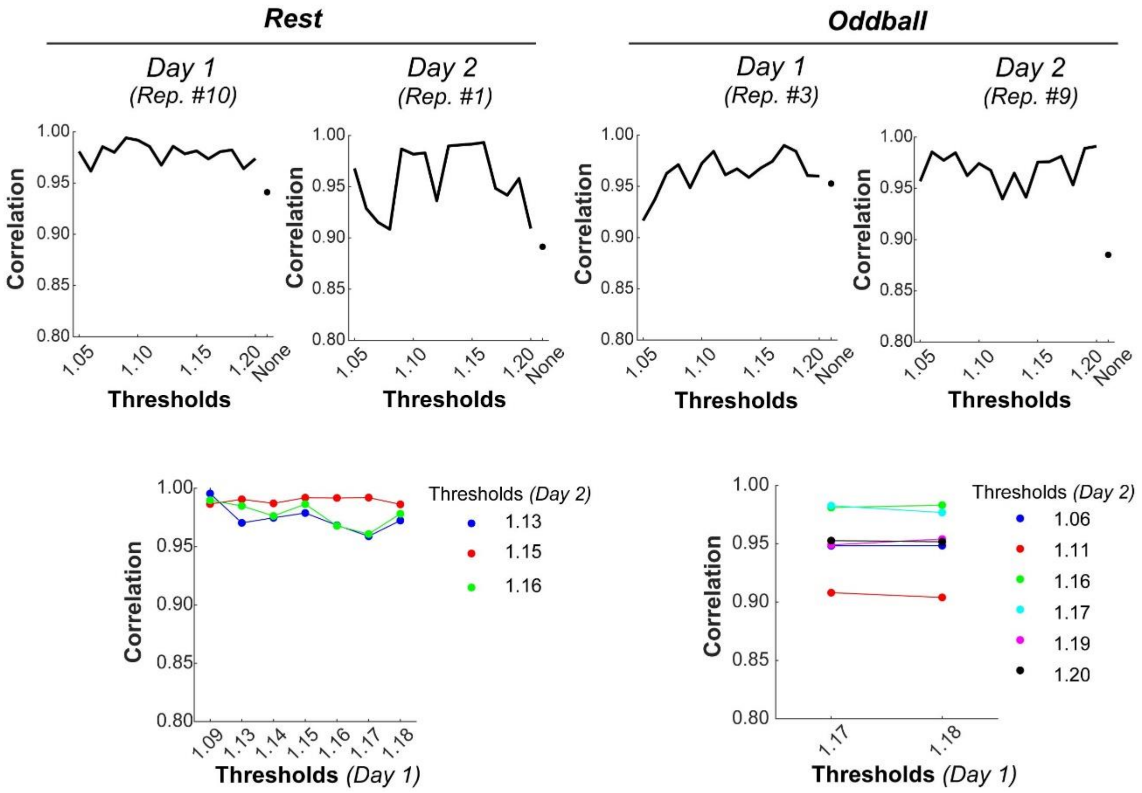

Supplementary Figure S4. Top panel: Spatial correlation coefficients for a pair of microstate templates of the same type (e.g., ms-A) between inside- and outside-EEG as a function of BCG thresholds on each recording day in resting-state (left) and oddball tasks (right). Bottom panel: Spatial correlation coefficients for a pair of inside-EEG on Days 1 and 2, with both being a selected replicate of the reduction in residual BCG artifacts, as a function of BCG thresholds on Day 1 in resting-state (left) and oddball tasks (right). Line with different colors represent BCG thresholds on Day 2. BCG: ballistocardiogram; EEG: electroencephalogram.

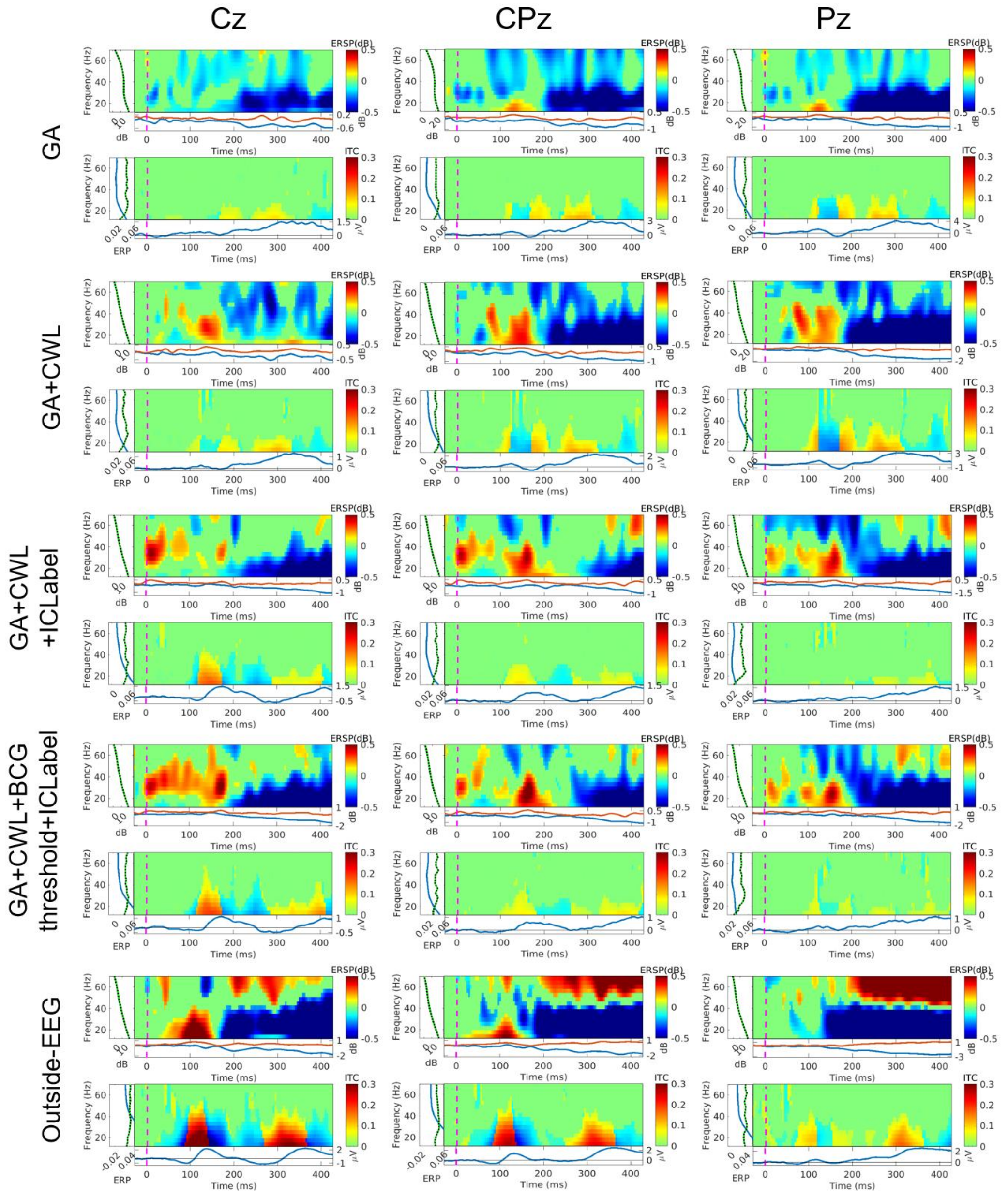

Supplementary Figure S5. Time-frequency analyses of ERPs across three selected posterior electrodes (Cz, CPz, and Pz) during trials with target stimuli in the oddball task. Top and bottom panels in each graph represent amplitude and inter-trial coherence, masked green with a threshold set by a two-tailed permutation probability significance level of .05. GA, CWL, and ICLabel respectively stand for GA correction, CWL regression, and artifact-component rejection with ICLabel. BCG threshold represents the final BCG threshold identified in Section 3.4. BCG: ballistocardiogram; CWL: carbon wire loop; GA: gradient artifact.

Descriptions of fMRI analyses (FiguresS6, S7, and S8)

We checked the quality of fMRI data recorded simultaneously with EEG to determine whether the data were typical or atypical. Here we concatenated fMRI data on Days 1 and 2 for each participant. Pipelines for preprocessing differed between resting-state and oddball data due to the nature of project underlying the present study. For resting state, we used fMRIPrep 23.0.2 (Esteban et al., 2019, 2020), which is based on Nipype 1.8.6 (Gorgolewski et al., 2011), with default parameters to preprocess structural, fieldmap and functional resting state scans. Using default parameters, we obtained functional scans in standard space (MNI152NLin2009cAsym; Fonov et al., 2009). The remaining processing steps were implemented in Python 3.9.0 and the nilearn 0.10.1 (https://github.com/nilearn/nilearn), nibabel 3.2.2 (Brett et al., 2022), and pybids 0.15.4 (Yarkoni et al., 2019) packages. The functional scan data were spatially smoothed (Gaussian kernel; 3mm FWHM) and bandpass filtered (0.01 to 0.1 Hz; Butterworth filter; 5th order; applied bi-directionally). Then, confounds, estimated by fMRIPrep, were regressed out. The considered confounds included 12 motion parameters (3D translation and rotation and their derivatives) and the first 6 principal components in CSF and white matter (Muschelli et al., 2014). We additionally scrubbed outlier volumes using framewise displacement (FD) (threshold=0.5) and std_dvars (threshold=3) metrics (Power et al., 2012). The residual data were submitted to group ICA (Varoquax et al., 2010). Finally, we visualized the identified component activation patterns and compared them to previously identified resting state networks (Smith et al., 2009).

For preprocessing and evaluating oddball data, we used SPM12 (Wellcome Trust Centre for Neuroimaging) following the protocol described in a previous study (Ogawa et al., 2018). The first 12 volumes were discarded to allow for T1 equilibration. The remaining data were corrected for slice timing and realigned to the mean image of that sequence to compensate for head motion. Next, the structural image was co-registered to the mean functional image and segmented into three tissue classes in the MNI space. The functional images were normalized and resampled in a 2 × 2 × 2 mm grid. Finally, they were spatially smoothed using an isotropic Gaussian kernel of 8 mm full-width at half maximum. To remove several sources of spurious variance along with their temporal derivatives, the linear regression was performed, including six motion parameters in addition to averaged signals over gray

matter, white matter, and cerebrospinal fluid. After this preprocessing, a general linear model (GLM) analysis was conducted using the onsets of target and normal stimuli as events while taking hemodynamic response function into account. The analysis consisted of a first level for individual data and a second level for group data. BrainNet Viewer 1.7 (Xia et al., 2013) was used for presenting results.

Results of the fMRI analyses

We checked the quality of fMRI data recorded simultaneously with EEG. Figure S6 shows results of a canonical ICA for BOLD signals in resting state. We compared our results to a study by Smith et al. (2009) who reported 10 well-matched pairs of brain networks between the BrainMap activation database with 29,671 participants (Fox & Lancaster, 2002; Laird et al., 2005) and their own resting-state dataset with 36 participants. Numbers in white rectangles in our Figure S2 corresponds to those in Figure 2 of the Smith et al. study, based on our visual assessment. We identified each of the 10 brain networks in resting state (see Supplementary Figure S7 for an entire set). For oddball, Figure S8 shows results of a GLM analysis of BOLD signals (target trials > normal trials). Red arrows correspond to the area commonly reported in the oddball literature (e.g., Huang et al., 2005) including SMG supramarginal gyrus on the left and right sides of the brain.

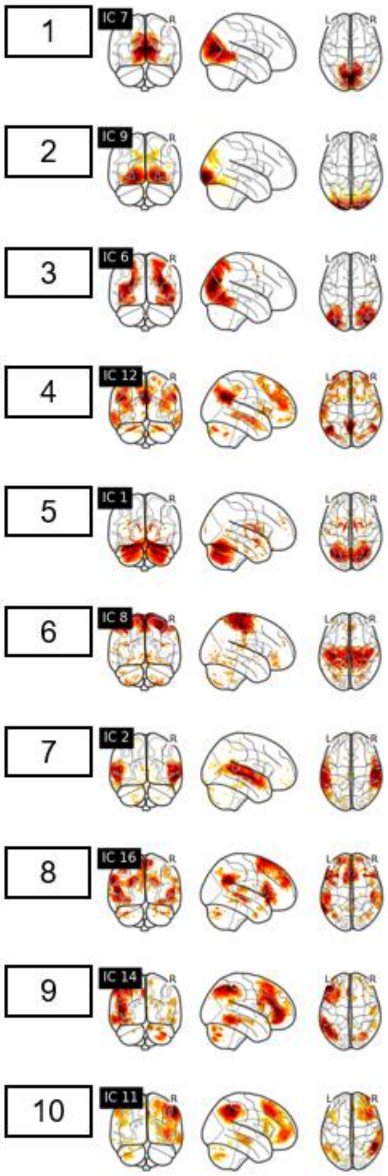

Supplementary Figure S6. Results of a canonical ICA on fMRI BOLD signals in resting state. Numbers in black rectangles are the order of components showed up in the present analysis whereas numbers in white rectangles are the component order in Figure 2 in Smith et al. (2009). ICA: independent component analysis; fMRI: functional MRI; BOLD: blood oxygen level dependent.

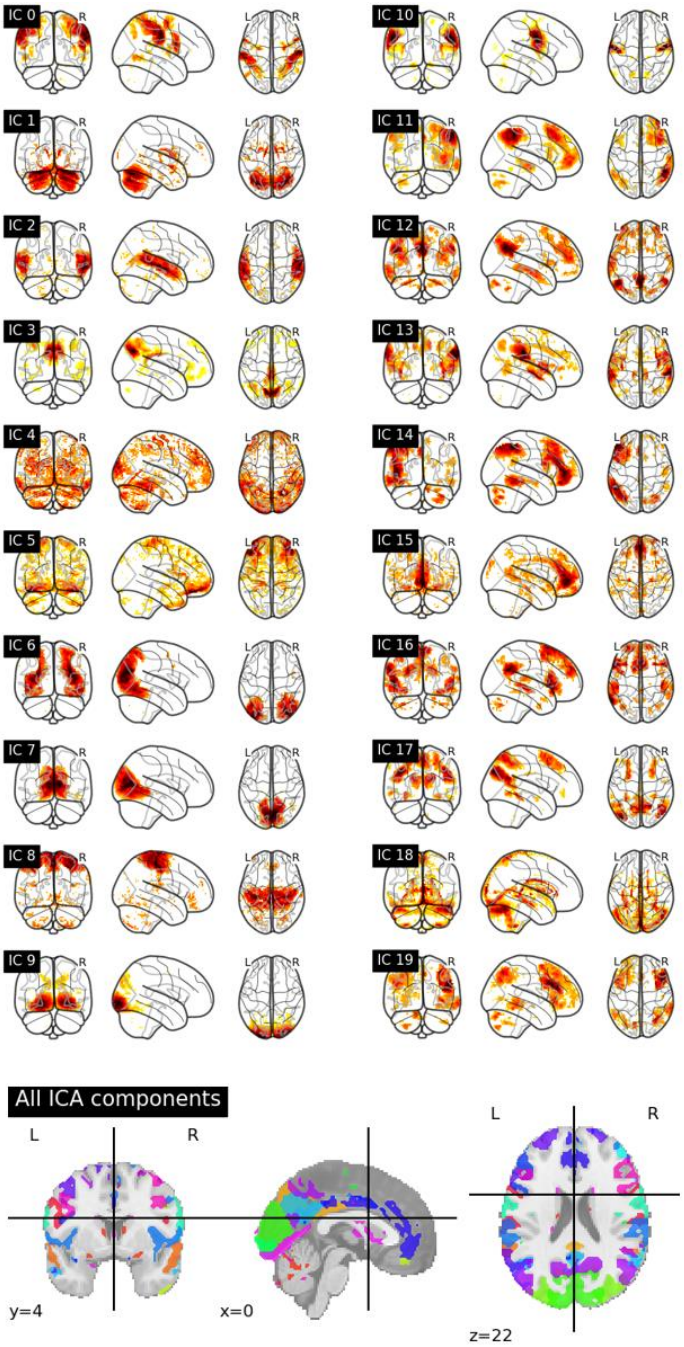

Supplementary Figure S7. An entire set of 20 components as a result of canonical ICA (top) and its summary on an atlas (bottom).

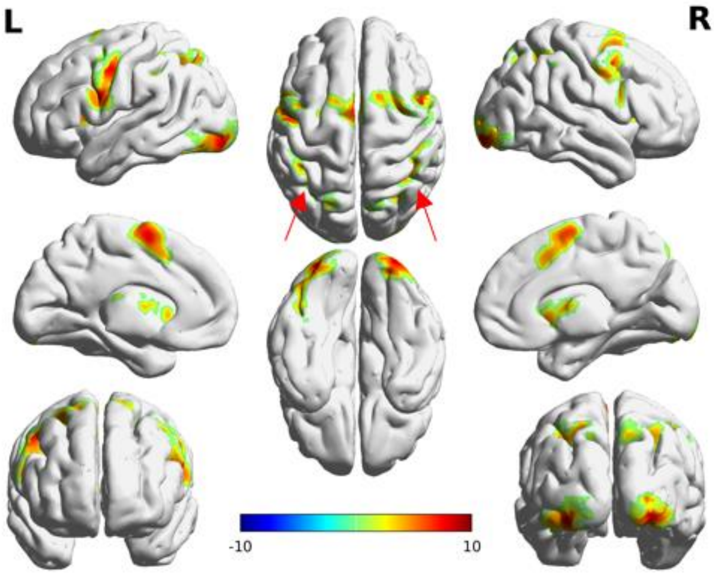

Supplementary Figure S8. Results of a GLM analysis of fMRI BOLD signals (target trials > normal trials) in the oddball task. Red arrows correspond to the area commonly reported recorded in the oddball literature. GLM outputs were sent to *BrainNet Viewer* for the graphical presentation here. BOLD: blood oxygen level dependent; fMRI: functional MRI; GLM: general linear model

References for Supplementary Materials

Brett, M., Markiewicz, C. J., Hanke, M., Côté, M.-A., Cipollini, B., McCarthy, P., Jarecka, D., Cheng, C. P., Halchenko, Y. O., Cottaar, M., Larson, E., Ghosh, S., Wassermann, D., Gerhard, S., Lee, G. R., Wang, H.-T., Kastman, E., Kaczmarzyk, J., Guidotti, R., Duek, O., et al. (2022). nipy/nibabel: 3.2.2 (3.2.2). Zenodo. https://doi.org/10.5281/zenodo.6617121

Esteban, O., Markiewicz, C. J., Blair, R. W., Moodie, C. A., Isik, A. I., Erramuzpe, A., Kent, J. D., Goncalves, M., DuPre, E., Snyder, M., Oya, H., Ghosh, S. S., Wright, J., Durnez, J., Poldrack, R. A., & Gorgolewski, K. J. (2019). fMRIPrep: a robust preprocessing pipeline for functional MRI. *Nature Methods, 16(1),* 111–116. https://doi.org/10.1038/s41592-018-0235-4

Esteban, O., Ciric, R., Finc, K., Blair, R. W., Markiewicz, C. J., Moodie, C. A., Kent, J. D., Goncalves, M., DuPre, E., Gomez, D. E. P., Ye, Z., Salo, T., Valabregue, R., Amlien, I. K., Liem, F., Jacoby, N., Stojić, H., Cieslak, M., Urchs, S., Halchenko, Y. O., et al. (2020). Analysis of task-based functional MRI data preprocessed with fMRIPrep. *Nature Protocols, 15(7),* 2186–2202. https://doi.org/10.1038/s41596-020-0327-3

Fonov, V. S., Evans, A. C., McKinstry, R. C., Almli, C. R., & Collins, D. L. (2009). Unbiased nonlinear average age-appropriate brain templates from birth to adulthood. *NeuroImage, 47(1)*, S102. https://doi.org/10.1016/S1053-8119(09)70884-5

Fox, P. T., & Lancaster, J. L. (2002). Opinion: Mapping context and content: The BrainMap model. Nature reviews. *Neuroscience, 3(4),* 319–321. https://doi.org/10.1038/nrn789

Gorgolewski, K., Burns, C. D., Madison, C., Clark, D., Halchenko, Y. O., Waskom, M. L., & Ghosh, S. S. (2011). Nipype: A flexible, lightweight and extensible neuroimaging data processing framework in python. *Frontiers in Neuroinformatics, 5,* 13. https://doi.org/10.3389/fninf.2011.00013

Laird, A. R., Lancaster, J. L., & Fox, P. T. (2005). BrainMap: The social evolution of a human brain mapping database. *Neuroinformatics, 3(1),* 65–78. https://doi.org/10.1385/ni:3:1:065

Muschelli, J., Nebel, M. B., Caffo, B. S., Barber, A. D., Pekar, J. J., & Mostofsky, S. H. (2014). Reduction of motion-related artifacts in resting state fMRI using aCompCor. *NeuroImage, 96*, 22–35.

https://doi.org/10.1016/j.neuroimage.2014.03.028

Ogawa, T., Aihara, T., Shimokawa, T., & Yamashita, O. (2018). Large-scale brain network associated with creative insight: combined voxel-based morphometry and resting-state functional connectivity analyses. *Scientific Reports, 8(1)*, 6477. https://doi.org/10.1038/s41598-018-24981-0

Power, J. D., Barnes, K. A., Snyder, A. Z., Schlaggar, B. L., & Petersen, S. E. (2012). Spurious but systematic correlations in functional connectivity MRI networks arise from subject motion. *NeuroImage, 59(3)*, 2142–2154. https://doi.org/10.1016/j.neuroimage.2011.10.018

Varoquaux, G., Sadaghiani, S., Pinel, P., Kleinschmidt, A., Poline, J. B., & Thirion, B. (2010). A group model for stable multi-subject ICA on fMRI datasets. *NeuroImage, 51(1),* 288–299. https://doi.org/10.1016/j.neuroimage.2010.02.010

Xia, M., Wang, J., & He, Y. (2013). BrainNet Viewer: A network visualization tool for human brain connectomics. *PloS one, 8(7),* e68910. https://doi.org/10.1371/journal.pone.0068910

Yarkoni et al., (2019). PyBIDS: Python tools for BIDS datasets. *Journal of Open Source Software, 4(40)*, 1294, https://doi.org/10.21105/joss.01294
